# Mechanistic Insights into Ni(II)-Catalyzed Nonalternating Ethylene–Carbon Monoxide Copolymerization

**DOI:** 10.1021/jacs.2c04563

**Published:** 2022-08-09

**Authors:** Maria Voccia, Lukas Odenwald, Maximilian Baur, Fei Lin, Laura Falivene, Stefan Mecking, Lucia Caporaso

**Affiliations:** † Department of Chemistry, University of Salerno, Via Giovanni Paolo II, 84084 Fisciano, Salerno, Italy; ‡ Chair of Chemical Materials Science, Department of Chemistry, University of Konstanz, 78464 Konstanz, Germany

## Abstract

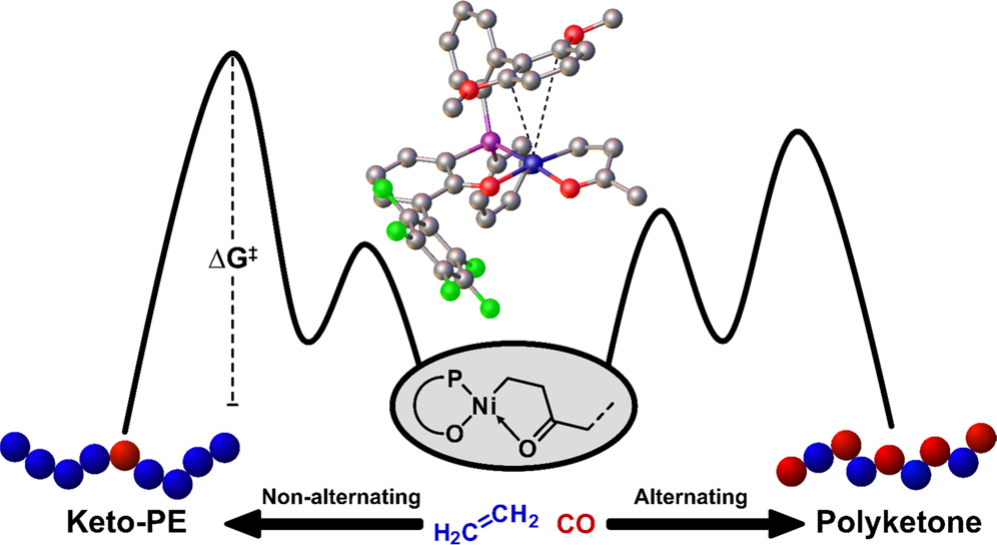

Polyethylene materials with in-chain-incorporated keto groups were recently enabled by nonalternating copolymerization of ethylene with carbon monoxide in the presence of Ni(II) phosphinephenolate catalysts. We elucidate the mechanism of this long-sought-for reaction by a combined theoretical DFT study of catalytically active species and the experimental study of polymer microstructures formed in pressure-reactor copolymerizations with different catalysts. The pathway leading to the desired nonalternating incorporation proceeds via the *cis*/*trans* isomerization of an alkyl-olefin intermediate as the rate-determining step. The formation of alternating motifs is determined by the barrier for the opening of the six-membered *C*,*O*-chelate by ethylene binding as the decisive step. An η^2^-coordination of a P-bound aromatic moiety axially oriented to the metal center is a crucial feature of these Ni(II) catalysts, which also modulates the competition between the two pathways. The conformational constraints imposed in a 2′,6′-dimethoxybiphenyl moiety overall result in a desirable combination of disfavoring ethylene coordination along the alternating incorporation pathway, which is primarily governed by electronics, while not overly penalizing the nonalternating chain growth, which is primarily governed by sterics.

## Introduction

An access to polyethylenes with in-chain keto units by incorporation of small amounts of carbon monoxide during ethylene polymerization has been long sought after. Among others, small amounts of such keto units can impart the material with desirable photodegradability to reduce the problematic environmental persistency of mismanaged polyethylene waste.^1^ Due to the strong relative binding and low insertion barriers of carbon monoxide relative to ethylene, catalytic copolymerizations usually afford strictly alternating copolymers.^2^ These are high melting materials with entirely different properties and application profiles than polyethylene. Note that copolymerizations of other functionalized vinyl monomers with CO are also found to occur by an alternating mechanism.^3,4^

The only known catalyst systems capable of introducing several consecutive ethylene units in addition to alternating motifs are neutral phosphinesulfonato Pd(II) complexes;^5^ these are recently complemented by cationic diphosphazane monoxide Pd(II) catalysts.^6^ However, either very high carbonyl contents resulting in material properties similar to alternating polyketones (*T*_m_ ∼ 200 °C) are obtained,^7^ or low-molecular-weight wax-like materials (*M*_n_ ≤ 3.000 g/mol) are formed, which impedes any study of material properties.^8−12^ Note this picture was altered only most recently by Nozaki et al., who succeeded in generating higher-molecular-weight linear polyethylene with isolated in-chain keto units employing metal carbonyls as a source of carbon monoxide in combination with advanced phosphinesulfonato Pd(II) catalysts.^13^ Considering the possible alternative catalysts, neutral Ni(II) catalysts have long been known to be capable of ethylene chain growth reactions,^14^ and recent developments pioneered by Shimizu et al. afforded catalysts that generate high-molecular-weight materials.^15−18^ Concerning the reactivity of neutral Ni(II) catalysts toward carbon monoxide, previous studies suggest that the phosphineenolato Ni(II) complexes investigated are sensitive for irreversible deactivation and form alternating polyketones at the most.^19^ With this background, it is all the more notable that the long-sought nonalternating copolymerization is enabled by advanced phosphinephenolato Ni(II) complexes. Copolymerization of ethylene at only 5 atm overall pressure with low concentrations of carbon monoxide yields polyethylenes with isolated keto groups. Among others, due to their high molecular weights (up to *M*_w_ 400.000 g/mol; *M*_n_ 200.000 g/mol), these polymers are processable and have mechanical properties that are at par with those of commercial high-density polyethylene (HDPE).^20^ At the same time, they are photodegradable. As an additional benefit, the catalysts are based on a non-noble earth-abundant metal.

These findings prompt the question of how nonalternating ethylene–CO chain growth is possible and occurs with these catalysts. We now report relevant pathways and barriers identified by extensive theoretical studies. These also provide the first insights into how the active sites’ catalytic properties are determined by their coordination environment in these reactions, which are correlated with the experimental observations of polymer microstructures formed in pressure-reactor copolymerizations.

## Results and Discussion

**Chart 1:**
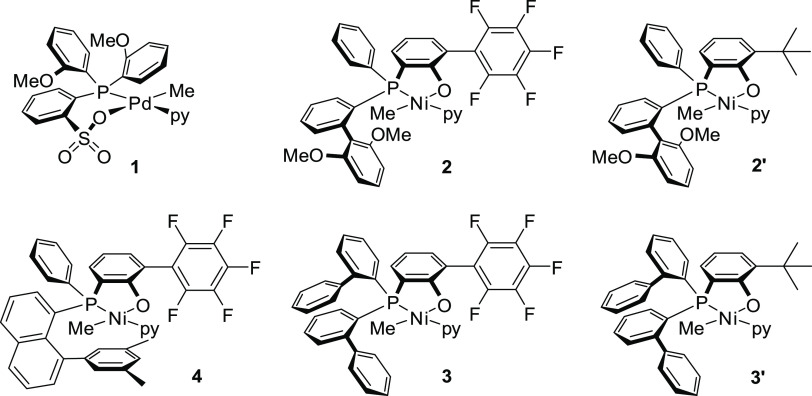
Ni(II) Phosphinephenolate Catalysts Studied, and Phosphinesulfonato Pd(II) Reference System

Several different instructive state-of-the-art phosphinephenolato Ni(II) catalysts with different substitution patterns and a phosphinesulfonato Pd(II) reference system were explored (Chart 1).

### Experimental Microstructures

To compare the polymer microstructures obtained with different catalysts, polymerization experiments with a short duration of 5 min and also otherwise identical conditions were performed (Table 1). For details of the procedure cf. the Supporting Information.

**Table 1:** Polymerization Results^a^

cat.	yield [mg]	TOF^b^	χ_NMR_^c^ [mol %]	microstr. I/NA/A^d^ [mol %]	insertions na/alt^e^
**1**	34	1.46	7.3	82:17:1	92:8
**2**	11	0.47	10.3	34:51:15	60:40
**2′**	25	1.07	8.4	56:39:5	75:25
**3**	37	1.59	10.8	19:51:30	45:55
**3′**	31	1.33	14.4	19:53:28	46:54
**4**	12	0.51	36.7	0:18:82	9:91

a Reaction conditions: 10 μmol catalyst precursor, 200 mL of toluene 90 °C, 0.02 bar ^13^CO, 5 min reaction time, 10 bar reaction pressure, and 1000 rpm (cf. SI for the polymerization procedure).

b TOF given in units of 10^3^ mol[C_2_H_4_] mol^–1^[Ni] h^–1^.

c Determined by ^1^H-NMR spectroscopy.

d I: isolated carbonyl, NA: nonalternating motifs, A: alternating motifs. Determined by ^13^C NMR spectroscopy (Figure S9) according to ref (20).

e Relative ratio of nonalternating and alternating carbon monoxide incorporation events, derived from the microstructure ((I + 0.5 NA)/(0.5 NA + A)).

Due to the limited reaction times, polymer yields and consequently also the conversion of monomers are low such that the polymerizations are performed under near steady-state conditions. Conditions in terms of temperature (90 °C) and monomer concentrations are chosen to facilitate the differentiation of the catalysts according to the polymer microstructures expected. All catalysts, including complexes **3** and **3′**, which have not been studied for CO copolymerization to date, yield copolymers comprising isolated keto groups, with the exception of **4**. However, the amount of isolated vs. adjacent nonalternating keto groups vs alternating motifs varies distinctly. Translated to the incorporation events during chain growth (Table 1), these microstructures correspond to a large range of strong preferences for nonalternating CO incorporation vs. alternating incorporation and vice versa.

### Theoretical Studies

**Figure 1:**
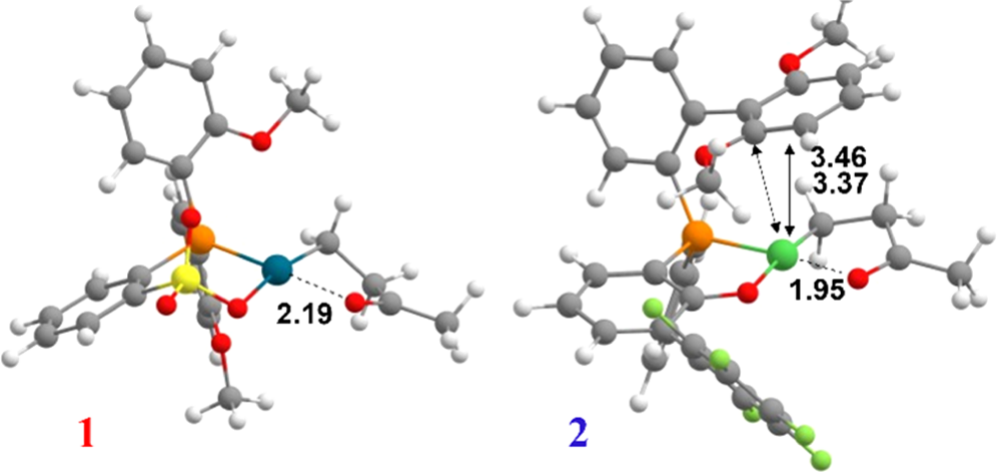
Geometries of the chelate **1-cycle5-T** intermediate for catalysts **1** (left) and **2** (right). Distances are in Å.

**Figure 2:**
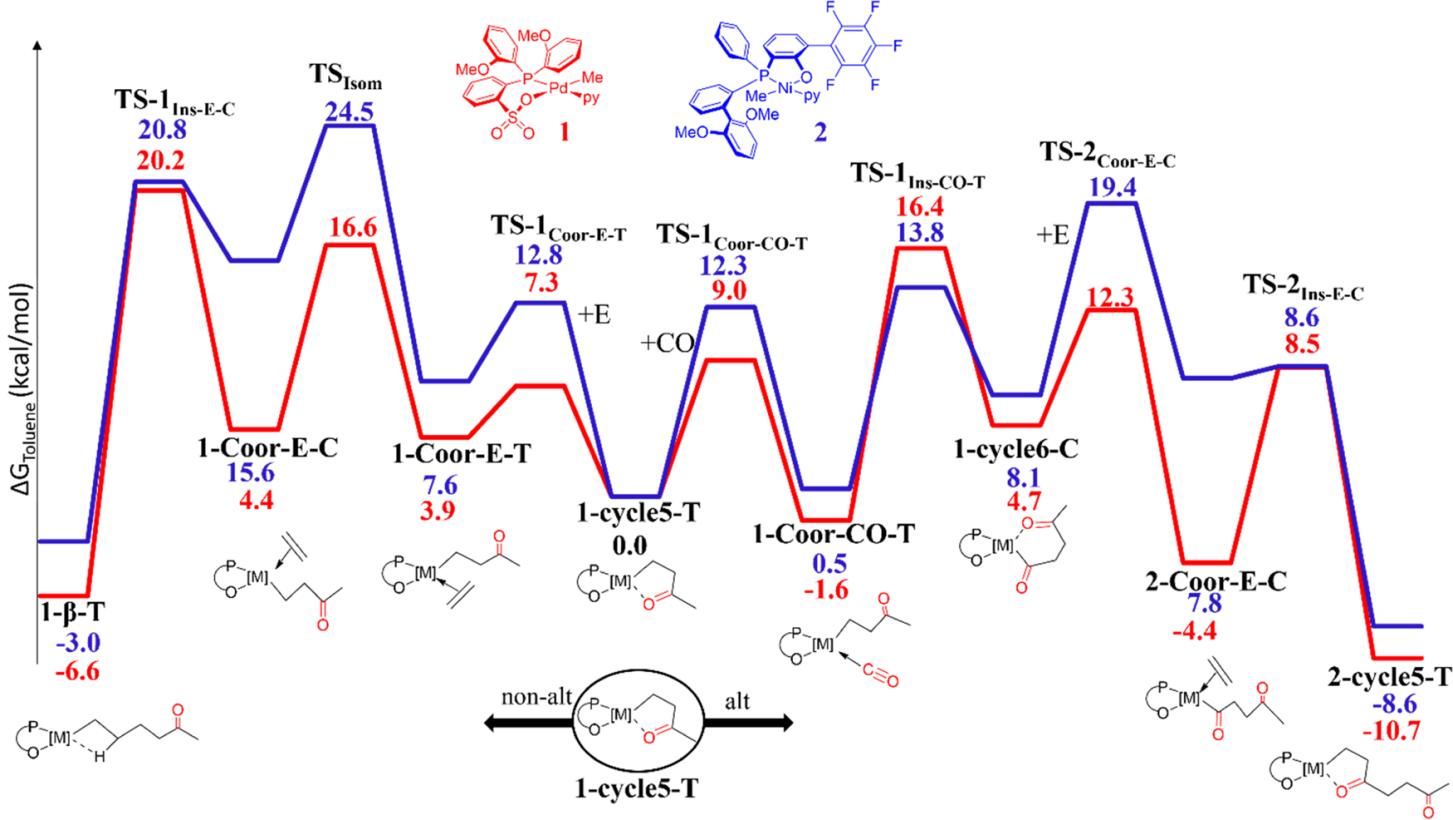
Free energies (Δ*G*_tol_ in kcal/mol) of the key steps for nonalternating and alternating carbon monoxide incorporation with catalysts **1** (red) and **2** (blue). The labels of the species in black (intermediates and TSs) refer to 1-* for species involved in monomer incorporation (E or CO) from **1-cycle5-T**, 2-* for the next (second) monomer (CO + E) incorporation.

The reaction pathways underlying the observed copolymerizations were explored by in-depth DFT studies. For all catalysts explored (Chart 1), the intermediate **1-cycle5-T** (cf. Figures 1 and 2) with the alkyl chain in the *trans* position to the oxygen atom models the growing polymer chain formed after the migratory insertion of an ethylene unit into the metal–acyl bond. This stable five-membered chelate is set as zero point energy for the entire ethylene and CO copolymerization pathway,^21^ in line with previous calculations reported by Ziegler et al.^22,23^ on the competition between the alternating and nonalternating E/CO copolymerization catalyzed by the reference Pd complex **1**. Here, we re-evaluate these mechanisms at a higher level of theory to use catalyst **1** as a reference for the nickel-based catalysts, taking into account a more complete reaction scenario; for more details on the other studied pathways, see Supporting Information.

Since **1-cycle5-T** is the starting point of both pathways giving access to the formation of nonalternating keto-polyethylene and alternating polyketone segments, respectively, we performed an in-depth structural analysis of this species to determine the stability and the strength of the metal···O interaction depending on the nature of the metal as well as on the chelating ligand structure.

As expected, for the palladium complex **1**, the Pd···O interaction is weaker compared to, e.g., nickel complex **2**, as reflected by a longer Pd···O distance in **1-cycle5-T** for **1** (see Figure 1) due to the greater electron density on the palladium center with respect to the nickel site.

It is worth noting that a η^2^ interaction of the aryl ring of the bis-phenyl moiety with the metal is observed for complex **2**, and this feature impacts the catalytic behavior (vide infra).

### Chain Growth Pathways

Starting from **1-cycle5-T**, we calculated all intermediates and transition states (TSs) along the catalytic pathways involved in the formation of alternating (**alt**) and nonalternating (**non-alt**) polymer motifs for complex **2** and for the reference complex **1** (see Figure 2). The reported free energies were obtained through solvent single point energy calculations on the BP86^24,25^ optimized geometries using the M06 functional and the triple-ζ TZVP^26−28^ with the Gaussian09 package;^29^ for more details, see the Supporting Information.

#### Nonalternating Chain Growth (**non-alt**)

This catalytic pathway is initiated by ethylene coordination to the alkyl–metal complex **1-cycle5-T**. This coordination occurs via **TS-1**_**Coor-E-T**_ by breaking the metal···O interaction and requires a free energy barrier of 7.3 and 12.8 kcal/mol for **1** and **2**, respectively, see Figure 2. The greater electron density present on the metal center for **1** clearly facilitates this step. For both complexes, the resulting **1-Coor-E-T** intermediate with the alkyl chain *trans* to the oxygen is higher in energy relative to **1-cycle5-T** + C_2_H_4_ considered at infinite distance, and this is more pronounced for **2** than for **1** (3.9 kcal/mol for **1** and 7.6 kcal/mol for **2**). This is likely due to the lower electron density present at the nickel center in the neutral (P,O)Ni complex, which causes a weaker binding of the ethylene as a result of a reduced back-donation from the metal to the olefin.

**Figure 3:**
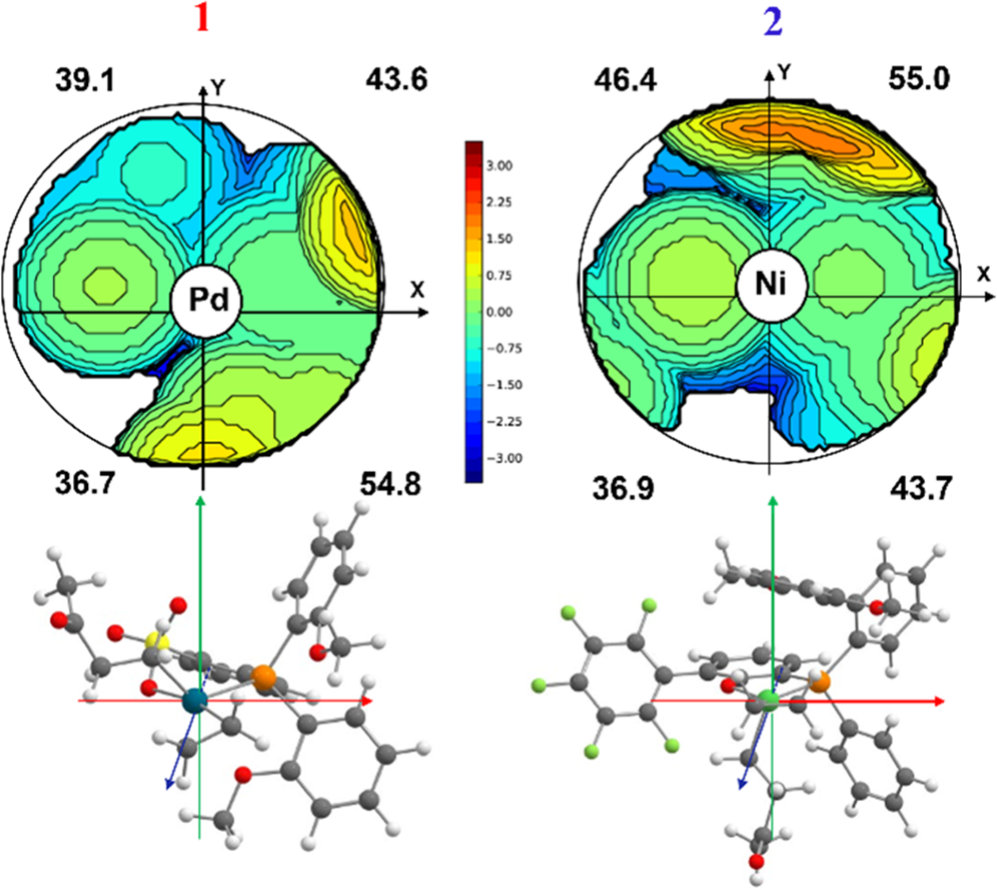
Topographic steric maps of the transition state **TS**_**Isom**_ for catalysts **1** (top left) and **2** (top right). The complexes are oriented as shown below (bottom left and right).^30^

From **1-Coor-E-T**, both complexes prefer to isomerize to the less-stable **1-Coor-E-C** intermediate and then insert ethylene. This pathway is preferred to direct ethylene insertion from **1-Coor-E-T**, which goes along with a significantly higher energy barrier (almost 10 kcal/mol higher with respect to the insertion from the isomer **1-Coor-E-C**; for details, see the Supporting Information). The isomerization step via the transition state **TS**_**Isom**_ requires a significantly higher energy barrier for the nickel complex compared to the palladium complex (16.6 and 24.5 kcal/mol for **1** and **2**, respectively) due to a much higher steric hindrance caused by the η^2^ coordination of the aryl ring to the metal in **2**, as evidenced by the topographic steric maps (compare NE and NW quadrants for **1** and **2** in Figure 3).

Once **1-Coor-E-C** is formed, the monomer insertion occurs via **TS-1**_**Ins-E-C**_ to yield the stable β-agostic complex, **2-β-T**. The overall free energy barrier from **1-cycle5-T** to **TS-1**_**Ins-E-C**_ amounts to 20.2 and 20.8 kcal/mol for **1** and **2**, respectively.

Notably, the monomer insertion barrier is similar for both complexes; however, for **1**, it represents the rate-determining step of the nonalternating pathway, while for **2**, it is lower than the *cis*/*trans* isomerization that becomes the rate-determining step for the nonalternating chain growth pathway due to steric impediments.

#### Alternating Chain Growth (**alt**)

Starting from **1-cycle5-T**, alternatively to ethylene coordination, CO coordination can take place, leading to the isoenergetic **1-Coor-CO-T** intermediate. The overall energy barrier for this process is, also in this case, higher for the nickel complex (12.3 kcal/mol for **2** vs 9.0 kcal/mol for **1**), in line with the greater strength of the Ni···O interaction with respect to the Pd···O one.

**Figure 4:**
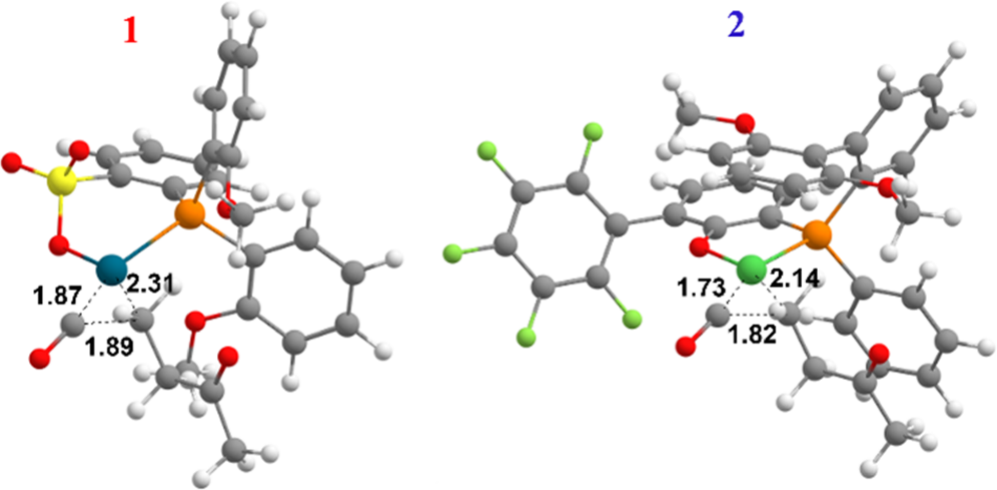
Geometry of **TS-1**_**Ins-CO-T**_ for catalysts **1** (left) and **2** (right). Distances are reported in Å.

From **1-Coor-CO-T**, CO insertion occurs via **TS-1**_**Ins-CO-T**_, yielding the six-membered chelate species, **1-cycle6-C**.^31^ The overall free energy barriers from **1-Coor-CO-T** to **TS-1**_**Ins-CO-T**_ amount to 18.0 and 13.8 kcal/mol for **1** and **2**, respectively. This difference is ascribed to a weaker σ-donation from the coordinated CO to a more electron-rich palladium center with respect to the nickel case, as confirmed by the increased metal–CO bond length in **TS-1**_**Ins-CO-T**_ for **1** with respect to **2**, see Figure 4.^32^

Notably, for **2**, the resulting chelate **1-cycle6-C** is unfavored by 8.1 kcal/mol, whereas for **1**, it is unfavored only by 4.7 kcal/mol. This difference is related again to the greater electron density on the Pd center that stabilizes the metal–acyl bond in the *trans* position to the phosphorus atom to a greater extent than that for **2**.

From **1-cycle6-C**, ethylene coordinates *trans* to the oxygen atom (**2-Coor-E-C**) through **TS-2**_**Coor-E-C**_ that is about 7 kcal/mol higher in energy for **2** than for **1** (compare barriers of 12.3 and 19.4 for **1** and **2**, respectively). This difference is related once again to the greater electron density present at the palladium center, which favors the opening of the chelate required for ethylene coordination.

For catalyst **2**, the resulting **2-Coor-E-C** intermediate is 12.2 kcal/mol less stable than the corresponding Pd intermediate (compare 7.8 kcal/mol for **2** and −4.4 kcal/mol for **1**, respectively). This is likely due to both the higher steric hindrance of the phosphine-bound aryl substituents and the reduced electron density on the metal center for **2**. From **2-Coor-E-C**, the following ethylene insertion occurs via **TS-2**_**Ins-E-C**_ (at 8.5 and 8.6 kcal/mol for **1** and **2**, respectively), yielding the stable five-membered chelate complex **2-cycle5-T** at −8.6 and −10.7 kcal/mol for **2** and **1**, respectively.

Notably, in the case of catalyst **1**, the rate-determining step is the CO insertion with an energy barrier of 18.0 kcal/mol, whereas in the case of catalyst **2**, the rate-determining step is ethylene coordination with an energy barrier of 19.4 kcal/mol.

#### Alternating vs Nonalternating Chain Growth for **1** and **2**

The pathways elucidated above underline that the five-membered chelate complex **1-cycle5-T** is the key intermediate of this copolymerization catalysis, opening the route to both the nonalternating (**non-alt**) and alternating chain growths (**alt**). The competition between these pathways determines the polymer microstructure.

Starting from **1-cycle5-T**, for both catalysts considered, both pathways are feasible and competitive with one another. Comparing the rate-determining energy barriers along the two pathways, i.e., ΔΔ*G*^‡^ (**non-alt**)–(**alt**) of 3.8 for **1** and 5.1 kcal/mol for **2**, it emerges that overall complex **2** is similar to complex **1** in the sense of featuring a ΔΔ*G*^‡^ (**non-alt**)–(**alt**) higher by only 1.3 kcal/mol.

This result agrees with the experimental finding of nonalternating keto groups and alternating motifs formed in pressure-reactor copolymerizations with these catalysts (Table 1). The slight preference for alternating incorporation found by theoretical methods is offset by the high ethylene/CO monomer ratio employed in the copolymerization experiments. The experimentally observed slightly higher portion of isolated keto units in the polymer with **1** compared to **2** agrees with the slightly lower ΔΔ*G*^‡^ (**non-alt**)–(**alt**) determined theoretically for **1**; for the detailed discussion on the comparison between the theoretical and the experimental ratio of non alternating propagation to alternating propagation segments, see SI.

The results outlined show the following: (I) Steric factors affect the nonalternating pathway. The η^2^ coordination of the aryl ring to the metal causes a greater steric hindrance for complex **2**, increasing the energy of the transition state with higher steric demand, i.e., the *cis*/*trans* isomerization. (II) Electronic factors affect the alternating pathway. The lower electron density at the Ni center favors the CO migratory insertion into the metal–alkyl bond and reinforces the Ni···O interaction, increasing the energetic barrier of ethylene coordination, which consequently becomes the decisive step.

### Impact of the Structure of Chelating Phosphinephenolates on Catalysis

#### Phosphine Donor Substituents

To elucidate the impact of the electronic and steric nature of chelating phosphinephenolate on the two competing pathways, catalysts **3** and **4** (Chart 1) were investigated. Since the ΔΔ*G*^‡^ (**non-alt**)–(**alt**) determined for **1** and **2** was found to correlate with the experimentally observed copolymer microstructures, we focused the calculations on the two crucial transition states.

Starting from **1-cycle5-T**, by comparing the rate-determining energy barriers along the two pathways, it emerges that ΔΔ*G*^‡^ (**non-alt**)–(**alt**) is 6.5 kcal/mol for **3** and 9.2 kcal/mol for **4**, which are 1.4 and 4.1 kcal/mol higher for **3** and **4**, respectively, compared to that for **2**. This trend qualitatively agrees with the experimentally observed microstructures (Table 1).

**Figure 5:**
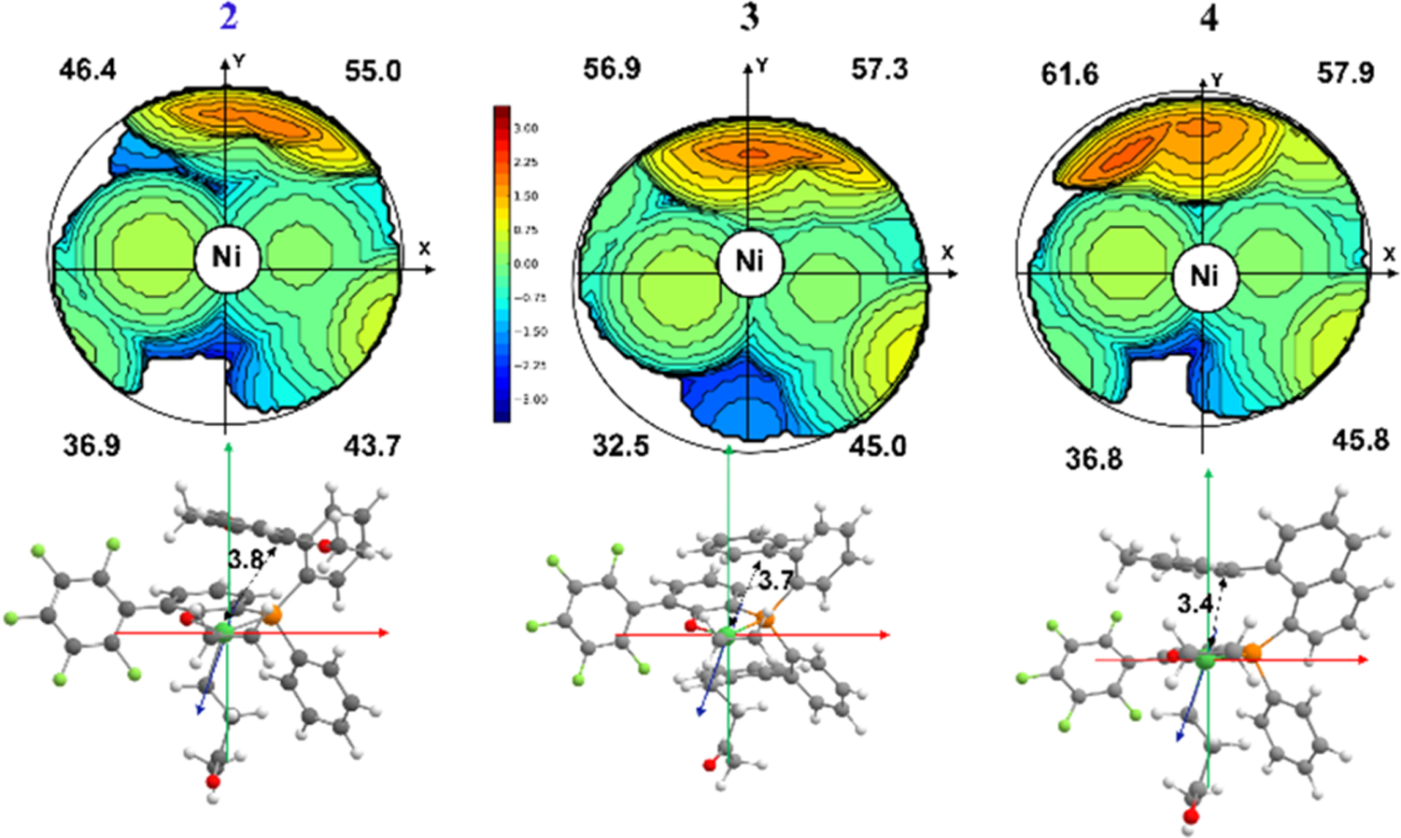
Topographic steric maps of transition state **TS**_**Isom**_ for catalysts **2** (left), **3** (in the center), and **4** (right). The Ni···C_ipso_ distances are reported in Å.

For **3**, the **non-alt** pathway suffers from the increased steric hindrance of the P-bound substituents, i.e., the additional aryl ring in the axial plane perpendicular to the coordination plane (compare SE quadrants in the steric maps of **TS**_**Isom**_ for **2** and **3** in Figure 5). Moreover, the absence of the OMe groups on the aryl moiety allows the ring to be in closer proximity to the Ni than in **2** (compare Ni–aryl distances in Figure 5). As a consequence, the energy barrier for the *cis*/*trans* isomerization increases. On the other hand, despite the shorter Ni···aryl ring distances for **3** than that for **2**, the lack of the electron-donating groups on the aryl ring of the bis-phenyl moiety reduces the electron density transferred to the metal for **3** compared to that for **2**. As result, for **3**, the Ni···O interaction in the chelate is stronger, increasing the energy barrier for ethylene coordination, i.e., **TS-2**_**Coor-E-C**_ (for the electronic analysis, see the Supporting Information). Overall, **3** favors the **non-alt** pathway to a lower extent with respect to **2**.

Moving to **4**, the aromatic ring on the naphthyl ligand interacts even more closely with Ni (see aryl–Ni distance in Figure 5), increasing both the steric hindrance around the catalytic center and the electron density on the metal due to the presence of the less encumbered and electron-donating methyl substituents on catalyst **4** compared to the larger methoxy groups on catalyst **2** (see electronic analysis in the Supporting Information). As a result, the Ni···O interaction in the chelate intermediate (i.e., **1-cycle5-T** and **1-cycle6-C**) weakens, lowering the ethylene coordination TS (**TS-2**_**Coor-E-C**_) to such an extent that the CO migratory insertion (**TS-1**_**Ins-CO-T**_) becomes the rate-determining step along the **alt** pathway. Moreover, for steric reasons (compare the Ni···C_ipso_ aryl ring distances reported in Figure 5 for catalysts **2** and **4**), the rate-determining *cis*/*trans* isomerization step along the **non-alt** pathway is even more penalized. Consequently, the ΔΔ*G*^‡^ (**non-alt**)–(**alt**) is higher for **4** than that for **3** and **2**, in line with the experimentally observed preference for alternating chain growth (Table 1).

#### Phenolate Substituents

As regards the impact of the substituent in the *o*-position on the phenolate moiety, for comparison to the pentafluorophenyl-substituted catalysts **2** and **3** their *tert*-butyl substituted analogues (**2′** and **3′**, Chart 1) were also explored.

**Figure 6:**
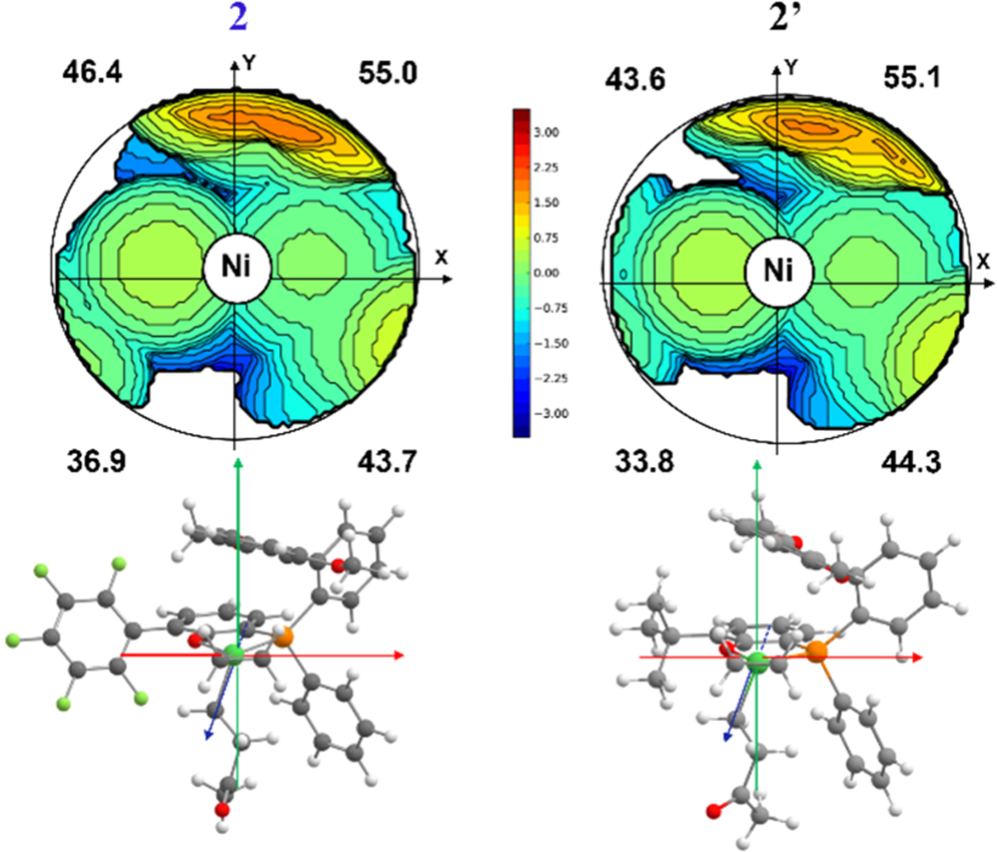
Topographic steric maps of **TS**_**Isom**_ for catalysts **2** (left) and **2′** (right).

From a steric point of view, **2′** and **3′** are similar to their C_6_F_5_ analogues **2** and **3** as suggested by the topographic steric maps and have similar values of the buried volume (%V_bur_) of the quadrants (see Figure 6 and the Supporting Information for **3** vs **3′**). Moreover, from an electronic analysis (see Supporting Information), the nature of the substituent does not alter the charge on the metal dramatically.

Indeed, the calculated ΔΔ*G*^‡^(**non-alt**)–(**alt**) of 5.7 kcal/mol for **2′** and 6.4 kcal/mol for **3′** are similar to those of 5.1 and 6.5 kcal/mol for the respective C_6_F_5_ analogues **2** and **3**, respectively. This is well in agreement with the experimentally observed smaller effect of the phenolate substitution compared to the phosphine substituents on the polymer microstructures (Table 1).

## Conclusions

The nonalternating copolymerization of ethylene with carbon monoxide to obtain polyethylene materials with in-chain ketone moieties was achieved recently by means of customized Ni(II) phosphinephenolate catalysts. Our combined theoretical and experimental study reveals the mechanism of chain growth of this long-sought-for reaction.

The rate-determining step of the pathway leading to the nonalternating incorporation of the two monomers is the *cis*/*trans* isomerization of the alkyl-olefin-intermediate. The formation of alternating motifs, instead, is determined by the opening of the six-membered chelate by ethylene coordination. The nature of the rate-determining step of either pathway differs from that of a phosphinesulfonate Pd(II) complex, which is studied here as a reference at the same level of theory.

The pathways and barriers identified for nonalternating vs. alternating incorporation of ethylene and carbon monoxide agree qualitatively with experimentally observed microstructures from pressure-reactor copolymerizations with catalysts varying in the phosphinephenolates’ structure.

In the Ni(II) phosphinephenolate catalysts studied, the desired nonalternating incorporation is the result of a favorable combination of electronic and steric factors. (1) A moderate steric hindrance of the phosphine moiety on the ligand facilitates the nonalternating path. (2) A balanced electronic donation to the electron-poor Ni center is desirable to avoid the formation of a too stable five-membered chelate resting state that would reduce the catalytic activity, while an increase in the stability of the six-membered chelate disfavors the undesired alternating path.

The aromatic rings on the phosphine moieties may provide a suitable electronic contribution when apically coordinated to the metal center, and easily clear out the catalytic site by moving away from the metal in the transition states with higher steric demand, i.e., the *cis*/*trans* isomerization and the ethylene coordination steps. In fact, the P-bound 2′,6′-dimethoxybiphenyl moiety in catalysts **2** and **2′** increases the energy barrier for ethylene coordination but does not impede the linear growth of the polyethylene chain.

We anticipate that these insights will also promote the discovery and the design of novel catalysts for this unique polymerization reaction, which provides in-chain-functionalized polyethylenes with a desirable property profile including lower environmental persistence.

## Supporting Information Available

Cartesian coordinates (XYZ)
Experimental procedures, characterization data, spectra, calculation details, alternative pathways investigated, steric and electronic analysis (PDF)

## Supplementary Material

ja2c04563_si_001.xyz
