# Supplementary material for: Mechanistic Insights into Ni(II)-Catalyzed Nonalternating Ethylene–Carbon Monoxide Copolymerization
Source: J Am Chem Soc. 2022 Aug 9;144(33):15111–7. doi: 10.1021/jacs.2c04563 (PMC9413223; doi:10.1021/jacs.2c04563)
Supplement: Supplementary file 2 — ja2c04563_si_002.pdf [file ja2c04563_si_002.pdf]

Supporting Information

Mechanistic Insights on Ni(II)-Catalyzed Non-Alternating Ethylene-Carbon Monoxide Copolymerization

M. Voccia,[a] L. Odenwald,[b] M. Baur,[b] F. Lin,[b] L. Falivene,[a] S. Mecking* [b], L. Caporaso* [a]

a) Department of Chemistry, University of Salerno, Via Giovanni Paolo II, 84084-Fisciano (SA), Italy.

b) Chair of Chemical Materials Science, Department of Chemistry, University of Konstanz, 78464 Konstanz, Germany.

Contents

1 General methods and materials 2
1.1 Solvents and reagents 2
1.2 Analytical methods and techniques 2
2 Synthesis and characterization of catalyst precursor **3** 3
3 Ethylene-CO copolymerizations and polymer analysis .... 10~~10~~
3.1 Procedure for ethylene-CO copolymerization .... 10~~10~~
3.2 Determination of CO incorporations from ^{1}H NMR spectra .... 10~~10~~
4 Computational details 14
5 Energetic profiles and Gibbs free energies. 15
5.1 Energetic profile with the alternative pathways for catalysts **1** and **2**. 15
5.2 Gibbs free energies of competitive species for catalysts **3**, **4**, **2’** and **3’** 16
5.3 Gibbs free energies of competitive species for catalyst **1** with different computational protocols 17
5.4 Determination of experimental and theoretical ratio of non-alternating propagation to alter nating propagation segments 17
6 Steric and electronic analysis .... 19~~19~~
6.1 Steric analysis .... 19~~19~~
6.2 Electronic analysis 20
7 Cartesian coordinates .... 21~~21~~
8 References .... 55~~55~~

1 General methods and materials

Unless noted otherwise, all manipulations of air and moisture sensitive materials were carried out under inert gas atmosphere using standard glovebox and Schlenk techniques.

1.1 Solvents and reagents

Solvents were dried and degassed using standard laboratory techniques. Oxygen was removed from ethyl acetate by freeze-pump-thaw degassing and storage over molecular sieves prior to use. Pentane and diethyl ether were dried and freed from oxygen with a MB-SPS-800 solvent purification system by MBRAUN and molecular sieves. Benzene was distilled from sodium. *n*-Butyllithium (*n*-BuLi; 2.5 M solution in *n*-hexane) and 3,4-dihydro-*2H*-pyran (≥97 %) were purchased from Sigma-Aldrich. [(tmeda)NiMe$_2$][1], 2-phenoxytetrahydro-2H-pyran[2], di([1,1'-biphenyl]-2-yl)chlorophosphane[3], and complex **1**[4], **2**[3], **2'**[5], **3'**[3] and **4**[6] were synthesized by reported procedures. Ethylene (grade 4.5) was supplied by Air Liquide and used as received. ^{13}CO with an isotopic purity of >99 % was purchased from Eurisotop. All other commercially available reagents and starting materials were supplied by Sigma Aldrich, Acros, ABCR or Activate Scientific. $CDCl_3$ was supplied by Sigma Aldrich, all other deuterated solvents by Eurisotop.

1.2 Analytical methods and techniques

NMR spectra were recorded on a Bruker Avance III HD 400 (^{1}H: 400.1 MHz, ^{13}C: 100.6 MHz, ^{19}F: 376.1 MHz), a Bruker Avance III 400 or a Bruker Avance III 600 spectrometer (^{1}H: 600 MHz, ^{13}C: 151 MHz). ^{1}H chemical shifts were referenced to the solvent's residual proton signals ($CDCl_3$: 7.26 ppm, $C_2D_2Cl_4$: 5.91 ppm, C_6D_6: 7.16 ppm). ^{13}C chemical shifts were referenced to the carbon signal of the deuterated solvent ($CDCl_3$: 77.16 ppm, $C_2D_2Cl_4$: 74.30 ppm, C_6D_6: 128.06 ppm). ^{19}F chemical shifts were referenced to external $BF_3 \cdot OEt_2$. Multiplicities are reported as follows: s (singlet), d (doublet), t (triplet), q (quartet), quint. (quintet), p (pentet), vm (virtual multiplet), m (multiplet), br. (broad). NMR spectra of polyethylenes were recorded with addition of 5 mg mL^{-1} of $Cr(acac)_3$ as paramagnetic relaxation agent.

2 Synthesis and characterization of catalyst precursor **3**

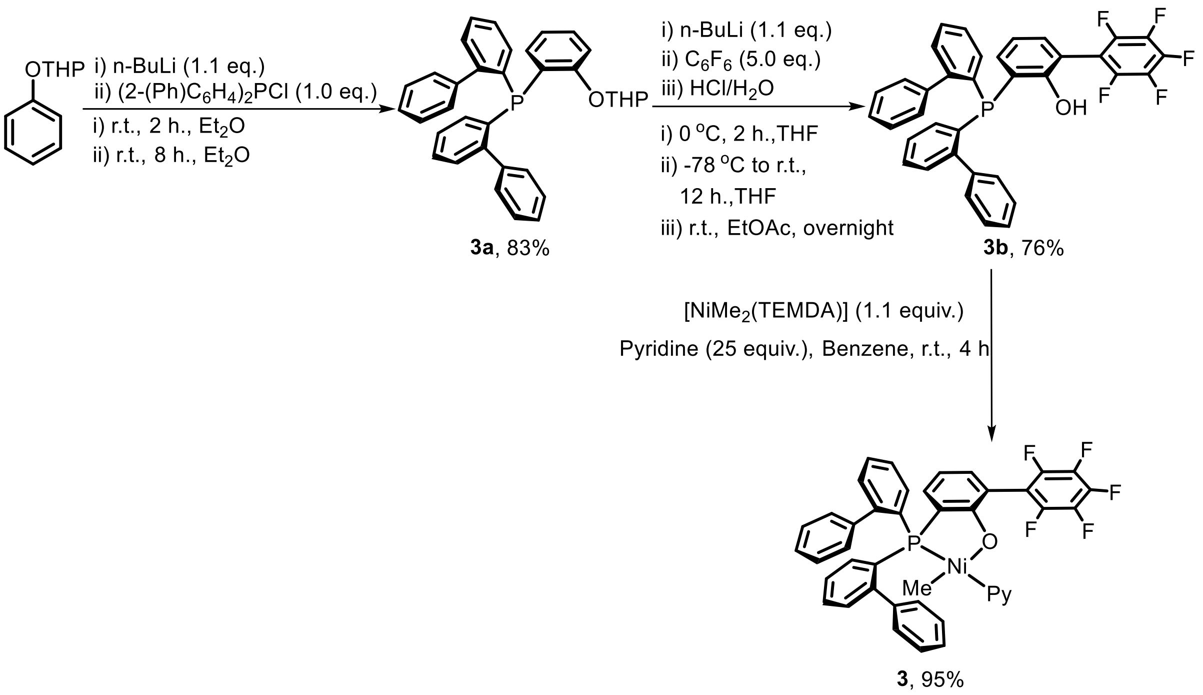

Scheme S1. Synthesis procedure for complex **3**.

(2-(Ph)C₆H₄)₂P-C₆H₄OTHP (3a): **3a** was prepared by modification of a reported procedure.[5] At room temperature n-BuLi (10 mL, 1.6 M in hexane, 16 mmol, 1.1 equiv) was added dropwise to a solution of 2-phenoxytetrahydro-2H-pyran (2.6 g, 14.5 mmol, 1.0 equiv.) in diethyl ether (30 mL). The reaction mixture was stirred for 2 hours, to yield a supension. A solution of (2-(Ph)C_6H_4)$_2$PCl (1.0 equiv.) in diethyl ether (30 mL) was added dropwise. After stirring for 8 h, all volatiles were removed under vacuum, and the residue was separated by column chromatography on silica using petrol ether/ethylene acetate = 20/1 as eluent to afford pure **3a** (6.2 g, 12.0 mmol, 83%).

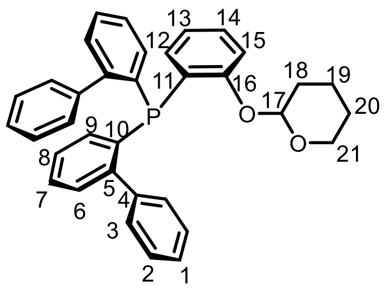

^{1}H NMR (400 MHz, benzene-d$_6$, 300 K) δ 7.47 – 7.37 (m, 2H, 8-H), 7.26 – 7.17 (m, 6H, 6-, 7-, and 9-H), 7.14 – 6.93 (m, 13H, 1-, 2-, 3-, 12-, 14- and 15-H), 6.83 – 6.74 (t, *J* = 7.6 Hz, 1H, 13-H), 5.09 (s, 1H, 17-H), 3.57 – 3.09 (m, 2H, 21-H), 1.37 – 0.80 (m, 6H, 18-, 19- and 20-H).

^{13}C {^{1}H} NMR (101 MHz, benzene-*d*$_6$, 300 K) δ 158.33 (d, *J* = 16.0 Hz, C16), 148.54 (t, *J* = 31.7 Hz,

C9), 142.26 (d, J = 6.5 Hz, C10), 142.16 (d, J = 6.4 Hz, C11), 135.03 (C14), 133.97 (d, J = 1.0 Hz, C8), 130.05 (d, J = 3.6 Hz, C6), 130.01 (d, J = 3.6 Hz, C4), 129.77 (d, J = 4.1 Hz, C3), 129.70 (C2), 129.66 (C1), 128.37 (d, J = 15.2 Hz, C5), 127.23 (d, J = 17.7 Hz, C12), 126.75 (d, J = 6.1 Hz, C15), 121.61 (C13), 114.11(C7), 95.74 (C17), 60.62 (C21), 29.77 (C18), 25.09 (C20), 17.58 (C19). The peaks for C_6F_5 are extremely broad due to multiple $^{x}J_{CF}$ couplings.

^{31}P {^{1}H} NMR (202 MHz, benzene-d_6, 300 K) δ -30.31.

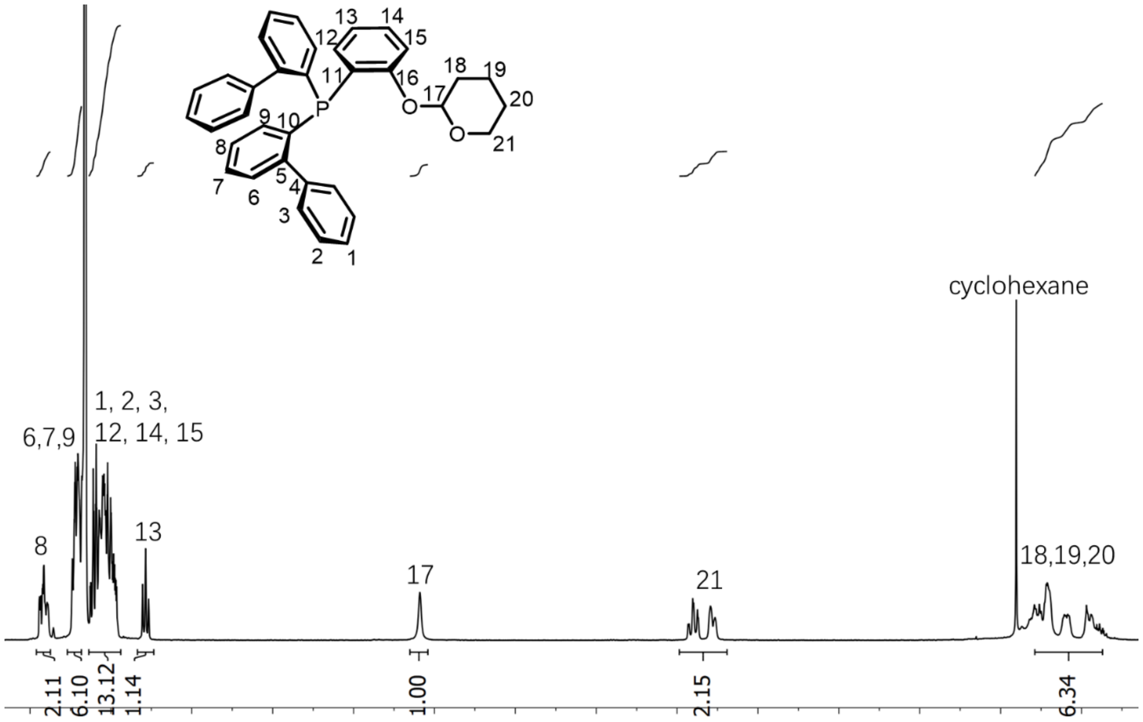

Figure S1. ^{1}H NMR spectrum of compound **3a** in C_6D_6 at 300 K.

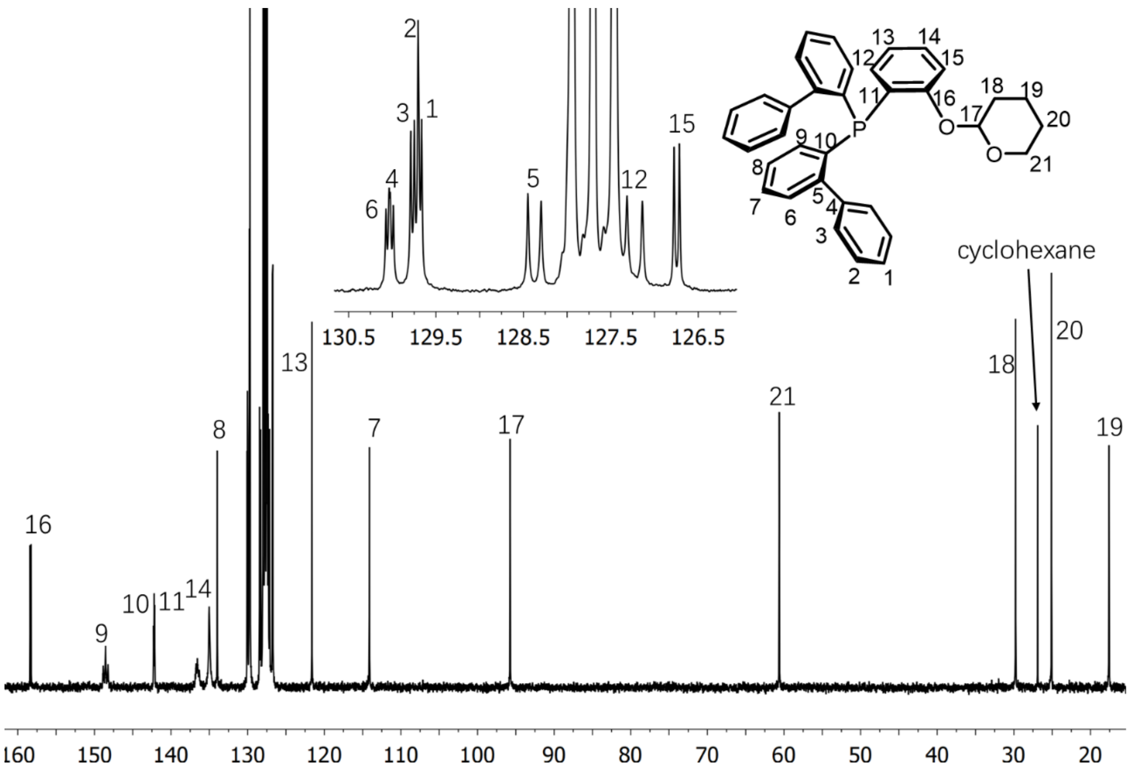

Figure S2. $^{13}C\{^1H\}$ NMR spectrum of compound **3a** in C_6D_6 at 300 K.

(2-(Ph)C_6H_4)$_2$P-6-C_6F_5-C_6H_3OH (3b): **3b** was prepared by modification of a reported procedure.[5] At 0 °C, *n*-BuLi (6.9 mL, 1.6 M in hexane, 11 mmol, 1.1 equiv.) was added dropwise to a solution of **3a** (5.1 g, 10 mmol, 1.0 equiv.) in THF (30 mL). The reaction mixture was warmed to room temperature and stirred for 2 hours to give a brown suspension. The mixture was cooled to -78 °C, and C_6F_6 (9.3 g, 50 mmol, 5.0 equiv.) was added dropwise at -78 °C. The mixture was warmed to room temperature slowly and stirred for 12 hours to afford a red solution. The mixture was concentrated in vacuum, the residue was dissolved in 20 mL of degassed ethyl acetate, and 2 mL of conc. HCl were added. The mixture was stirred at room temperature overnight and added slowly to a solution of 5 g $NaHCO_3$ in 60 mL water and stirred for 30 minutes. The organic phase was separated, and the aqueous phase was extracted with ethyl acetate (2 × 20 mL). The combined organic phase was concentrated in vacuo, and the residue was subjected to column chromatography on silica using petrol ether/ethyl acetate = 20/1 as eluent to give pure **3b** (4.5 g, 7.6 mmol, 76%).

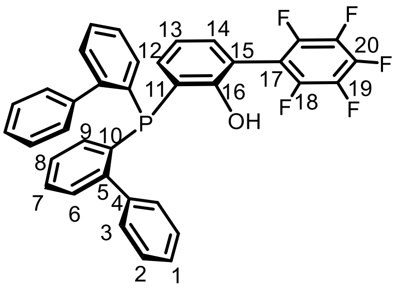

^{1}H NMR (400 MHz, benzene-d_6, 300 K) δ 7.35 – 7.30 (m, 2H, 8-H), 7.28 (ddd, J = 7.6, 4.2, 1.7 Hz, 1H, 14-H), 7.12 – 7.00 (m, 14H, 1-, 2-, 3-, 6-, 7-H and O*H*), 6.97 (td, J = 7.2, 1.4 Hz, 2H, 9-H), 6.72 (t, J = 7.6 Hz, 1H, 13-H), 6.54 (d, J = 9.6 Hz, 1H, 12-H).

$^{13}C\{^{1}H\}$ NMR (101MHz, benzene-d_6, 300 K) δ 157.37 (d, J = 22.0 Hz, C16), 147.83 (d, J = 27.5 Hz, C9), 141.41 (d, J = 6.2 Hz, C6), 137.55 (d, J = 1.7 Hz, C14), 133.73 (d, J = 5.2 Hz, C8), 133.68 (C11), 133.42 (C12), 130.17 (d, J = 5.1 Hz, C4), 129.37 (C7), 129.33 (C2), 129.03 (C10), 127.63 (C3), 127.17 (C1), 122.35 (d, J = 6.7 Hz, C15), 120.75 (d, J = 1.5 Hz, C13), 113.51 (C5), 112.42 (t, J = 19.7 Hz, C17). The peaks for C_6F_5 are extremely broad due to multiple $^{x}J_{CF}$ couplings.

$^{19}F\{^{1}H\}$ NMR (376 MHz, benzene-d_6, 300 K) δ -140.51 – -140.64 (m), -156.01 (t, J = 21.5 Hz), -163.39 (td, J = 23.4, 7.9 Hz).

$^{31}P\{^{1}H\}$ NMR (202 MHz, benzene-d_6, 300 K) δ -47.49.

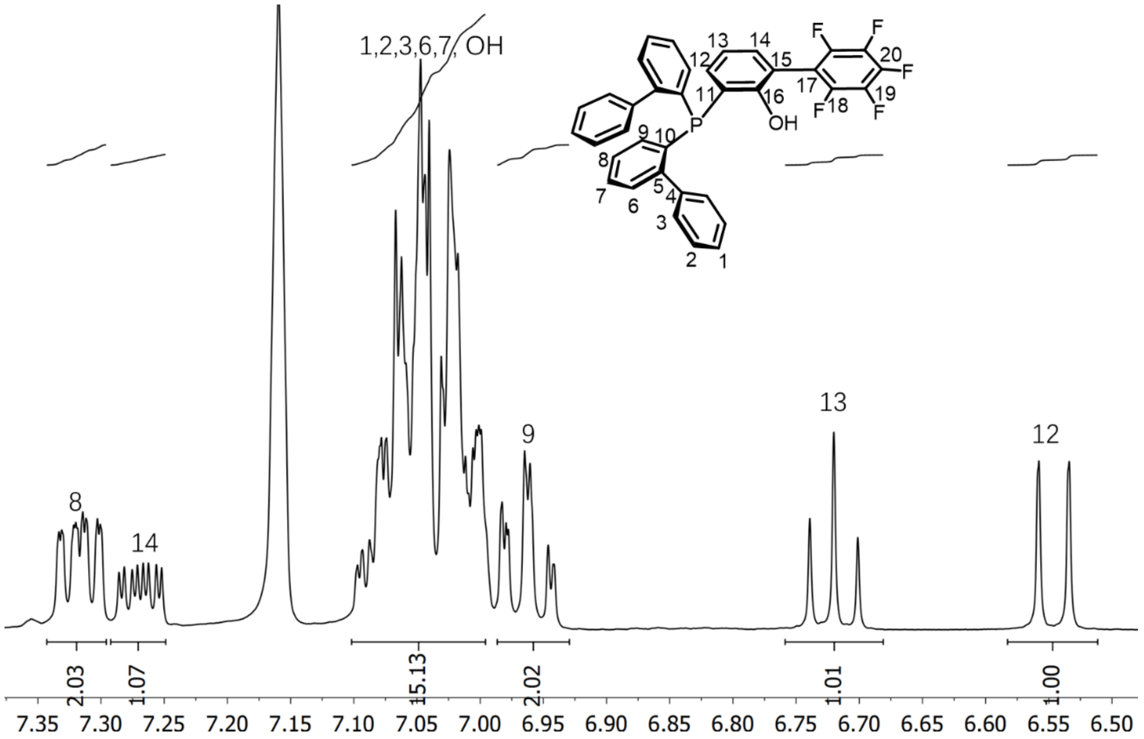

Figure S3. ^{1}H NMR spectrum of compound **3b** in C_6D_6 at 300 K.

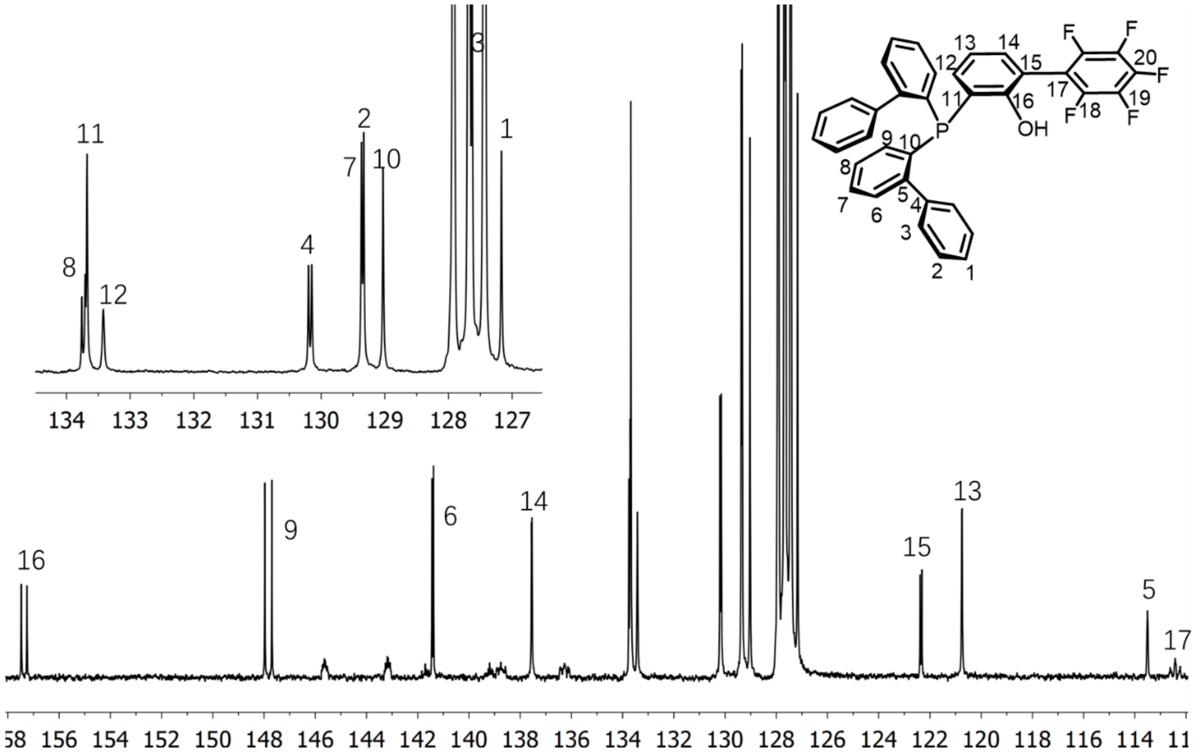

Figure S4. $^{13}C\{^1H\}$ NMR spectrum of compound **3b** in C_6D_6 at 300 K.

[2-{(2-Ph-C_6H_4)$_2$P}-6-C_6F_5-C_6H_3O]NiMe(pyridine) (3): To 23 mg [(tmeda)NiMe$_2$] (110 μmol, 1.1 equiv.) and 60 mg phosphinephenol **3b** (100 μmol, 1.0 equiv.), a solution of pyridine (25 equiv.) in 5 mL of benzene was added. Gas evolution (methane) was observed, and the reaction mixture turned yellow. After stirring for 4 hours at room temperature, volatiles were removed in vacuo. The residue was dissolved with 5 mL benzene, and any nickel black formed during the reaction was removed via centrifugation. The yellow solution was vitrified by cooling the flask with liquid nitrogen, and the solvent removed by freeze-drying to give the desired product **3** as a yellow powder (95 % yield, 95 μmol, 71 mg).

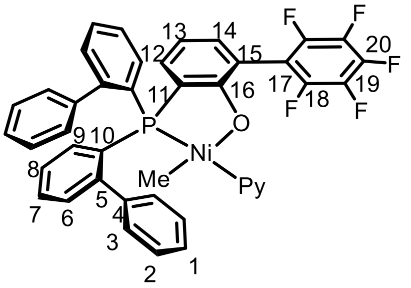

^{1}H NMR (400 MHz, benzene-d_6, 300 K) δ 8.30 (d, J = 5.5 Hz, 2H, 8-H), 7.84 (b, 2H, o-pyr), 7.60 – 7.45 (m, 2H, m-pyr), 7.14 – 6.88 (m, 9H), 6.77 (t, J = 7.6 Hz, 1H), 6.48 (t, J = 6.7 Hz, 2H), 6.30 (td, J = 7.5, 1.8 Hz, 1H), -1.08 (d, J = 5.2 Hz, 3H, Ni-CH_3).

$^{13}C\{^1H\}$ NMR (101 MHz, benzene-d_6, 300 K) δ 171.01 (d, J = 22.0 Hz, C16), 150.11 (o-pyr), 147.75

(d, J = 12.8 Hz, C9), 141.79 (C6), 136.27 (m-pyr), 134.29 (d, J = 56.3 Hz, C11), 133.61 (d, J = 2.6 Hz, C8), 132.39 (C15), 131.92 (d, J = 7.6 Hz, C12), 129.59 (C3), 129.26 (C1), 128.22 (C14), 127.02 (C2), 126.10 (d, J = 7.2 Hz, C4), 123.12 (d, J = 48.3 Hz, C10), 122.77 (p-pyr), 115.81 (t, J = 19.3 Hz, C17), 115.22 (d, J = 11.9 Hz, C5), 112.88 (d, J = 7.1 Hz, C13), -13.62 (d, J = 37.7 Hz, Ni-CH_3). C7 is overlapped by C_6D_6, the peaks for C_6F_5 are extremely broad due to multiple $^{x}J_{CF}$ couplings.

$^{31}P\{^1H\}$ NMR (162 MHz, benzene-d_6, 300 K) δ 20.14.

Elemental analysis for $C_{42}H_{29}F_5NOPNi$: found (calculated) [%]: C 66.53 (67.41), H 4.51 (3.91), N 1.73 (1.87).

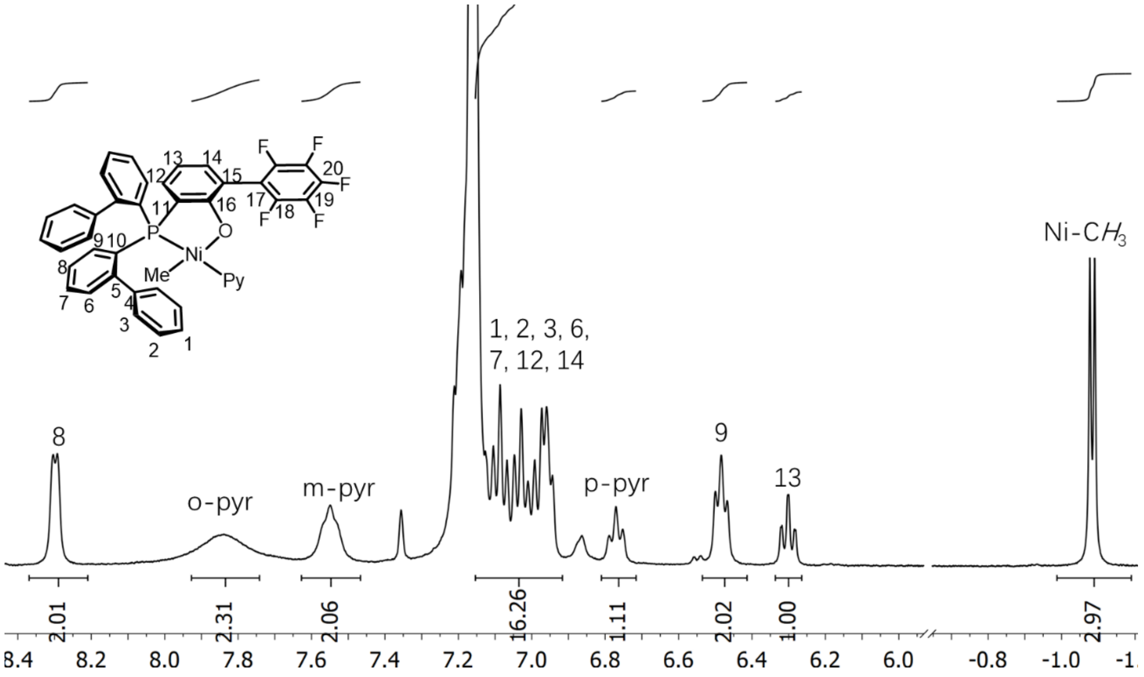

Figure S5. ^{1}H NMR spectrum of complex **3** in C_6D_6 at 300 K.

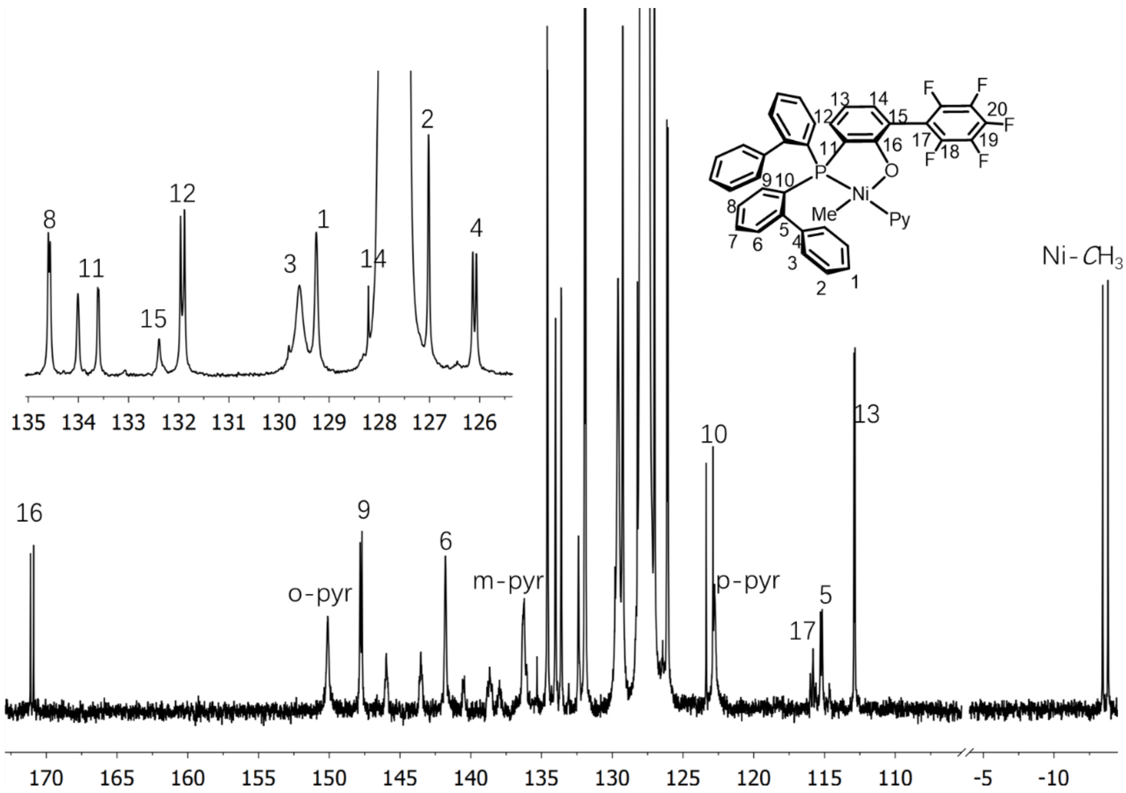

Figure S6. $^{13}C\{^{1}H\}$ NMR spectrum of complex **3** in C_6D_6 at 300 K.

3 Ethylene-CO copolymerizations and polymer analysis

3.1 Procedure for ethylene-CO copolymerization

Ethylene-carbon monoxide co-polymerizations were conducted in a *Büchi ecoclave* reactor with a 600 mL vessel. The reactor was equipped with a heating and cooling jacket connected to a thermostat, a mechanical stirrer (*Büchi Cyclone c075dc)*, a nitrogen/vacuum supply, a thermocouple dipping into the reaction mixture controlling the thermostat and a liquid dosing Pump (Knauer P 4.1s). A *Bronkhorst* MassFlow apparatus consisting of two flow meters (up to 20 g L^{-1} and 200 g L^{-1} ethylene), a pressure meter and a compressed air-driven badger valve was used to work under constant pressure. All gas valves and devices were connected to a *HiTec Zang LabBox* and operated by *HiTec Zang LabVision*® software (ver. 2.13). Prior to all polymerization experiments, the reactor was evacuated and heated (thermostat temperature: 90 °C). When the internal reactor temperature exceeded 60 °C, the reactor was flushed with nitrogen and evacuated three times. Then the temperature was adjusted to the desired reaction temperature. 200 mL of dry and degassed toluene were added, stirring was started with 100 rpm and the system was equilibrated for 5 min to reach the desired (internal) temperature. The system was pressurized with ^{13}C labeled carbon monoxide (starting from between 0.99 to 1.02 bar nitrogen pressure, adding 20 mbar of CO. This corresponds to ca. 8 to 9 mg of CO). The CO supply was disconnected and the system was pressurized to a total pressure of 10 bar using an ethylene mass flow regulator. The precatalyst was dissolved in 4 mL of toluene and added to the reactor via the liquid dosing pump with 10 mL per minute. The reaction time was started upon begin of addition. The tubing of the pump was flushed with toluene for 3 min to assure the complete addition of the precatalyst. The reaction was kept under constant pressure for 5 minutes (using ethylene mass-flow regulators), and then the pressure was released. To the reaction mixture methanol was added (~50 mL), followed by evaporation of the solvent. The residue was washed with 50 mL of methanol applying ultrasonication, centrifuged off and then dried at 60 °C/30 mbar overnight.

3.2 Determination of CO incorporations from ^{1}H NMR spectra

Compositions of ethylene-CO copolymers were determined from ^{1}H NMR spectra according to equation (1).

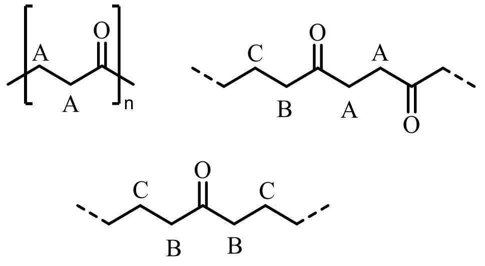

A: 2.55 - 2.84 ppm

B: 2.24 - 2.54 ppm

C: 1.47 - 1.65 ppm

D: 0.71 - 1.46 ppm

$$\chi_{CO} = 100 \cdot \frac{A + B}{2 \cdot A + 2 \cdot B + C + D} \quad (1)$$

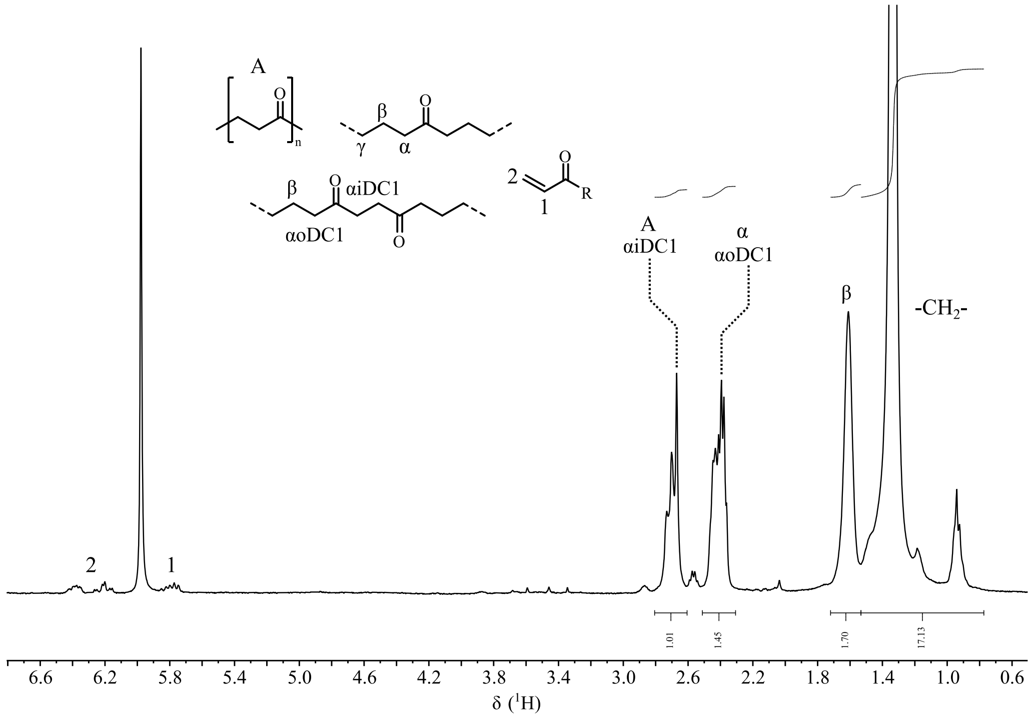

Figure S7. Exemplary ^{1}H NMR spectrum of an ethylene-CO copolymer produced with catalyst **2** with assignment of typical motifs.

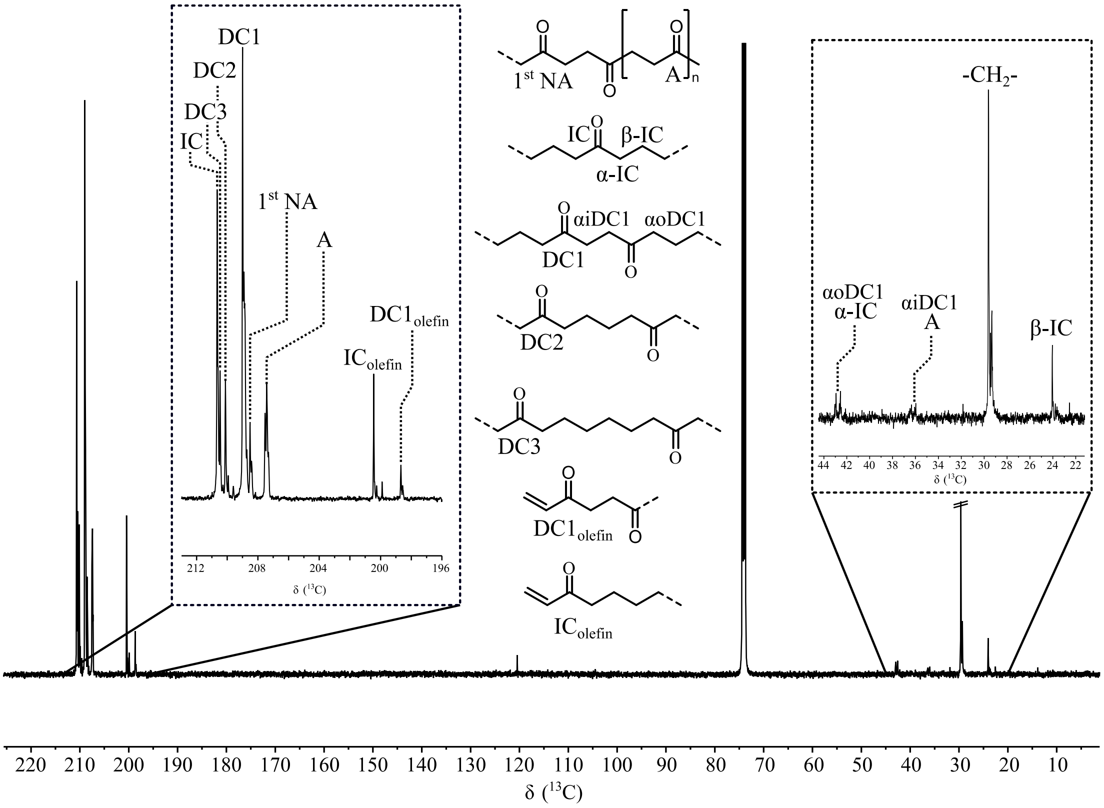

Figure S8. Exemplary ^{13}C NMR spectrum of an ethylene-CO copolymer produced with catalyst **2** with assignment of typical motifs.

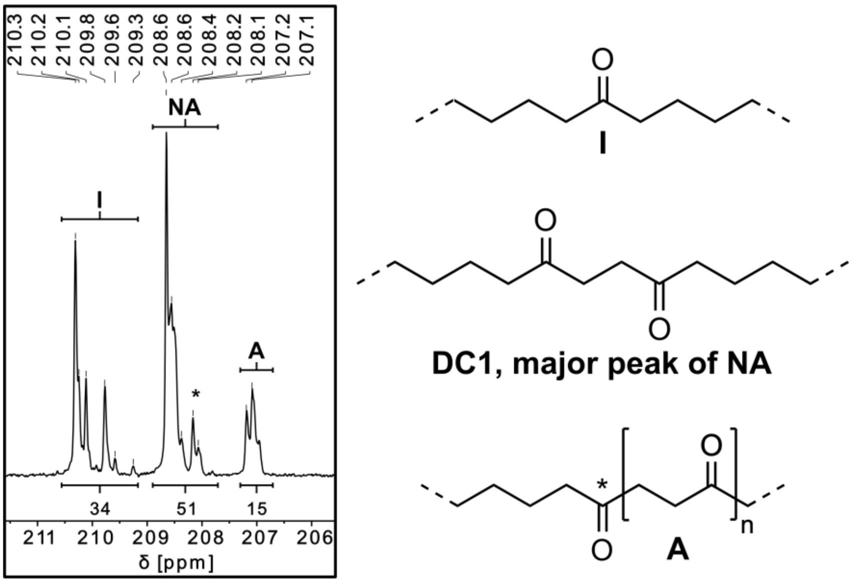

Figure S9. Exemplary $^{13}C\{^{1}H\}$ NMR spectrum of a copolymer (obtained with catalyst **2**, cf. Table 1, entry 2) with assignments of repeat unit motifs. In terms of CO incorporation events during polymerizaton, isolated motifs (I) correspond to an incorporation along the non-alternating pathway, alternating motifs (A) correspond to an incorporation *along the alternating pathway, and non-alternating motifs (NA) correspond to a* combination of an alt-pathway and a subsequent non-alt pathway. Thus, the relative ratio of non-alternating and alternating carbon monoxide incorporation events can be derived from the microstructure as (I + 0.5 NA) / (0.5 NA + A).

4 Computational details

We performed calculations of all important intermediates and transition states involved in the competitive linear chain growth alternating and non-alternating pathways during the ethylene and CO copolymerization (the **1-cycle5-T** intermediate was set as a reference point). All the DFT geometry optimizations were performed at the GGA BP86[7] level with the Gaussian09 package.[8] The electronic configuration of the systems was described with the 6-31G basis set for H, C, N, F, and O while for Ni the quasi relativistic LANL2DZ ECP effective core potential was adopted.[9] All geometries were characterized as minimum or transition state through frequency calculations. The geometry optimizations were performed without symmetry constraints. All transition-state structures were confirmed to connect corresponding reactants and products by intrinsic reaction coordinate (IRC) calculations.[10] The reported free energies were built through single point energy calculations on the (BP86/6-31G/LANL2DZ ECP) geometries using the M06 functional and the triple-ζ TZVP basis set on main group atoms while for Ni the quasi relativistic LANL2DZ ECP effective core potential was adopted.[9,11] Solvent effects were estimated with the PCM model using toluene as solvent.[12] To this (M06/TZVP/LANL2DZ ECP) electronic energy in solvent, thermal corrections were included from the gas-phase frequency calculations at the gas-phase level of theory (BP86/6-31G/LANL2DZ ECP). The percent buried volume calculations and the steric maps were performed with the SambVca 2.1 package.[13] The radius of the sphere was fixed in the origin of the metal center, while for the atoms, we adopted the Bondi radii scaled by 1.17, and a mesh of 0.1 Å was used to scan the sphere for buried voxels.

5 Energetic profiles and Gibbs free energies.

5.1 Energetic profile with the alternative pathways for catalysts **1** and **2.**

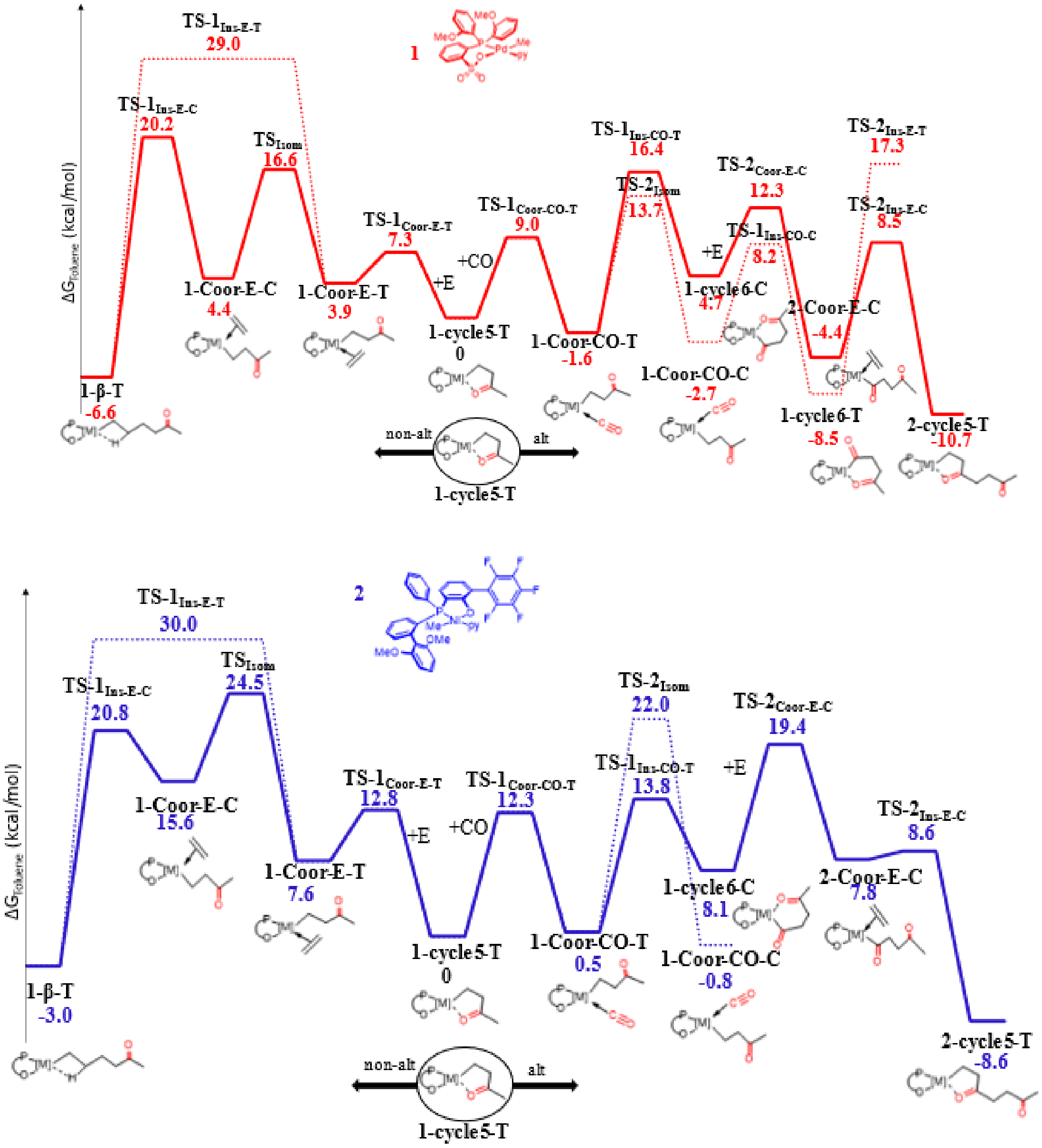

Figure S10. Gibbs free energies (ΔG_{Tol} in kcal/mol) of the competitive pathways for non-alternating and alternating carbon monoxide incorporation with catalysts **1** (red, top) and **2** (blue, bottom).

5.2 Gibbs free energies of competitive species for catalysts **3**, **4**, **2’** and **3’**.

Table S1: Gibbs free energies in Toluene (kcal/mol) of competitive species for catalysts **3** and **4**, with the same computational protocol (M06/ triple-ζ TZVP).

	Catalyst **3**	Catalyst **4**
1-cycle5-T	0.0	0.0
TS_{Isom}	27.1	23.8
$TS\text{-}2_{Coor\text{-}E\text{-}C}$	20.6	---
$TS\text{-}1_{Ins\text{-}CO\text{-}T}$	---	14.6
$\Delta\Delta G^{\ddagger}$ (**non-alt**)-(**alt**)	6.5	9.2

Table S2: Gibbs free energies in Toluene (kcal/mol) of competitive species for catalysts **2’** and **3’**, (M06/ triple-ζ TZVP).

	Catalyst **2’**	Catalyst **3’**
1-cycle5-T	0.0	0.0
TS_{Isom}	24.5	25.9
$TS\text{-}2_{Coor\text{-}E\text{-}C}$	18.8	19.5
$\Delta\Delta G^{\ddagger}$ (**non-alt**)-(**alt**)	5.7	6.4

5.3 Gibbs free energies of competitive species for catalyst **1** with different computational protocols.

Table S3: Gibbs free energies in Toluene (kcal/mol) of competitive species for catalyst **1** calculated with different computational methods. Solvent single point energy calculations (Toluene as solvent) on the BP86 optimized geometries using a) PBEO/D3 functional[14,15], b) B3PW91/D3[16] and c) SMD solvent model .[17]

Catalyst **1**	a) PBE0/D3	b) B3PW91/D3	c) SMD
1-cycle5-T	0.0	0.0	0.0
TS-1$_{\text{Ins-E-C}}$	19.7	20.2	20.1
TS-1$_{\text{Ins-CO-T}}$	14.6	15.0	13.2
ΔΔG‡ (**non-alt**)-(**alt**)	5.1	5.1	6.9

5.4 Comparison of propensity to non-alternating vs. alternating chain propagation determined by DFT methods and from experimentally observed microstructures.

In agreement with the equations reported by Ziegler[18] the theoretical ratio of non-alternating propagation to alternating propagation ($\boldsymbol{f_{na}}$) can be evaluated as:

$$f_{na} = \frac{r_{na}}{r_a} = \left(\frac{k'_{\text{C2H4}}}{k_{CO}}\frac{[C2H4]}{[CO]}\frac{K_1}{K_2}\right)\left(1 - \frac{k_{CO}^{-1}}{K_4 * k_{C2H4} * [C2H4] + k_{CO}^{-1}}\right)^{-1} = F_B \times F_{cor}$$

In detail, r_{na} is the probability to obtain non-alternating segments, whereas r_a is the probability to obtain alternating segments.

The equilibrium and kinetic constants of the equation refer to the following scheme:

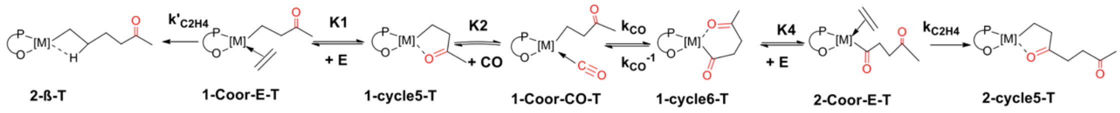

Since the CO pressure of 0.02 bar is estimated to correspond to a concentration of [CO] ≈ 1.7 × 10^{-4} mol L^{-1} (at 90°C in toluene)[19] and the ethylene (10 bar) concentration is estimated to [E] ≈ 0.67 mol L^{-1} (from data at 95 °C),[20] the calculated f_{na} value for catalyst **1** is 20.0.

It worth to note that the contribution of $F_{cor}$to f_{na} is negligible because k_{CO}^{-1} is << $K_4 * k_{C2H4}$ [C_2H_4] (indeed F_{cor} is 1).

In other words, the value of f_{na} corresponds to Brookhart's equation[21]:

$$f_{na} = \frac{r_{na}}{r_a} = \left(\frac{k'_{C2H4}}{k_{CO}}\frac{[C2H4]}{[CO]}\frac{K_1}{K_2}\right)$$

Moving to the Ni catalysts, from calculations it emerges that the $\mathbf{TS_{Isom}}$ shows the highest barrier along the non-alt path and the overcoming of the barrier that corresponds to the opening of the six membered chelate by ethylene is demanded for the formation of the alternating sequences (k''_{C2H4} << k_{C2H4} see the scheme below). For this reason, the f_{na} theoretical value can be calculated based on the following equation:

$$f_{na} = \frac{r_{na}}{r_a} = \left(\frac{k'_{C2H4}}{k_{CO}}\frac{[C2H4]}{[CO]}\frac{K_1}{K_2}\right)\left(1 - \frac{k_{CO}^{-1}}{k''_{C2H4} * [C2H4] + k_{CO}^{-1}}\right)^{-1} \cong \left(\frac{k'_{C2H4}}{k''_{C2H4}}\frac{1}{[CO]}\frac{K_1}{K'_2}\right)$$

where K'_2 is the equilibrium constant between of **1-cycle5-T** and **1-cycle6-T**.

This corresponds to the scheme:

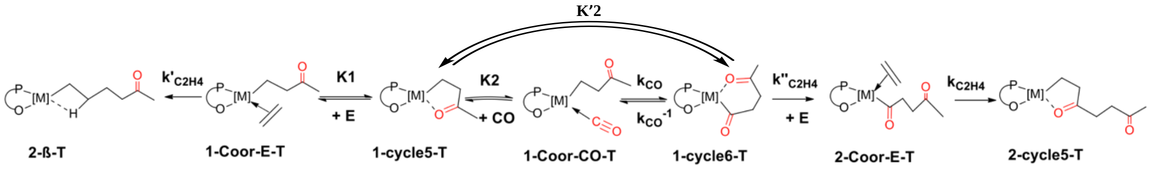

The f_{na} value obtained for catalyst **2**, **3** and **4** are 4.9 (mol $L^{-1})^{-1}$,0.69 (mol $L^{-1})^{-1}$and 0.15 (mol $L^{-1})^{-1}$ respectively.

Comparison of these theoretical value f_{na} to experimental values from microstructure analysis (Table 1) agrees reasonably (Table S4).

Table S4: Comparison of DFT results and experimental values derived from polymer microstructures of propensity for non-alternating vs. alternating CO incorporation.

Species	Experimentally determined f_{na}	Computed f_{na} [a](mol L^{-1}) $^{-1}$
Catalyst **1**	11.5	20.0
Catalyst **2**	1.5	(4.9) [a]
Catalyst **3**	0.8	(0.69) [a]
Catalyst **4**	0.1	(0.15) [a]

6 Steric and electronic analysis

6.1 Steric analysis

From a steric point of view, **2’** and **3’** are similar to their C_6F_5-analogues **2** and **3** as suggested by the topographic steric maps and the similar values of the buried volume (%Vbur) of the quadrants, (see Figure 6 for **2** vs. **2’** in the manuscript, and Figure S11 for **3** vs. **3'**).

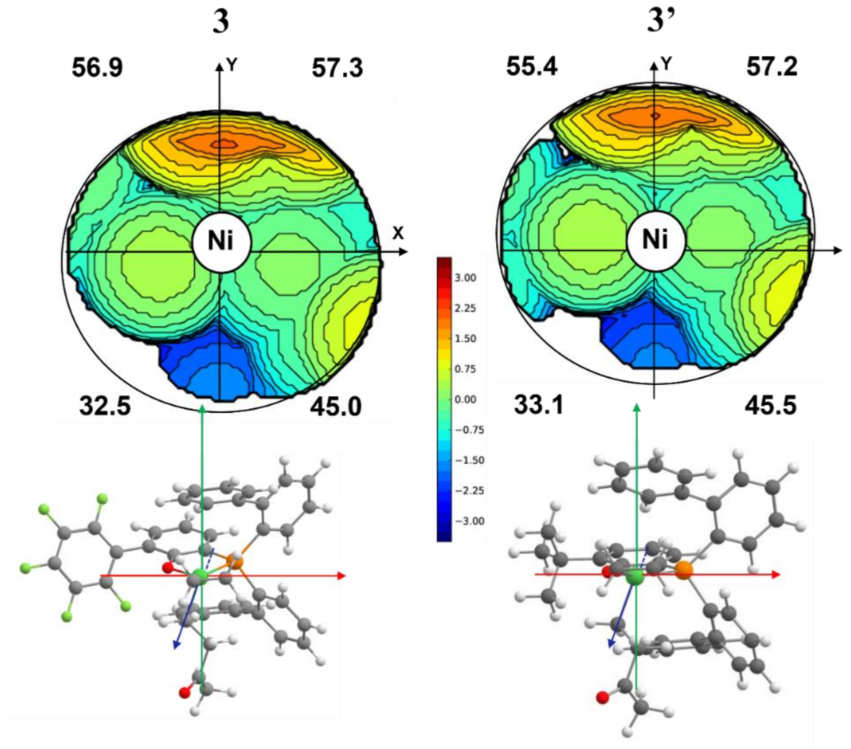

Figure S11. Topographic steric maps of the transition state **TS$_{Isom}$** for catalysts **3** (top left) and **3'** (top right). The complexes are oriented as shown below (bottom left and right).

6.2 Electronic analysis

Table S5. Natural bond orbital analysis on Nickel for **TS-2$_{Coor-E-C}$** on catalysts **2** and **3** catalysts and for **TS-1$_{Ins-CO-T}$** on catalysts **2** and **4**.

Charge on Ni atom	**2**	**3**	**4**
TS-2$_{Coor-E-C}$	0.19264	0.19407	
TS-1$_{Ins-CO-T}$	0.15441		0.08933

Table S6. Natural bond orbital analysis on Nickel for **TS-2$_{Coor-E-C}$** on catalysts **2'** and **3'**.

Charge on Ni atom	**2'**	**3'**
TS-2$_{Coor-E-C}$	0.18447	0.18728

7 Cartesian coordinates

Catalyst 1

Non-Alternating pathway

1-cycle5-T

Zero-point correction= 0.434963 (Hartree/Particle)
Thermal correction to Energy= 0.468927
Thermal correction to Enthalpy= 0.469871
Thermal correction to Gibbs Free Energy= 0.368309
Sum of electronic and zero-point Energies= -2246.878274
Sum of electronic and thermal Energies= -2246.844309
Sum of electronic and thermal Enthalpies= -2246.843365
Sum of electronic and thermal Free Energies= -2246.944927
E solvent= -2246.70981748

Pd	-1.332520000	-0.773301000	-0.514146000
P	0.536201000	0.366181000	0.087975000
C	1.522184000	1.209993000	-1.229496000
C	0.258086000	1.662192000	1.379782000
C	1.763038000	-0.760186000	0.914937000
C	-0.474471000	1.311423000	2.550293000
O	-0.881707000	0.008506000	2.622681000
C	-1.540978000	-0.438402000	3.810649000
H	-2.493245000	0.102560000	3.975898000
H	-0.891745000	-0.317394000	4.698786000
H	-1.746599000	-1.505862000	3.644631000
C	-0.723127000	2.272372000	3.549773000
H	-1.284454000	2.004471000	4.449264000
C	-0.236656000	3.581059000	3.395521000
H	-0.433021000	4.319805000	4.181110000
C	0.499725000	3.938109000	2.257974000
H	0.890473000	4.954765000	2.144509000
C	0.741616000	2.977671000	1.261067000
H	1.329559000	3.249021000	0.379095000
C	0.960581000	2.247900000	-2.023954000
O	-0.316324000	2.632576000	-1.700718000
C	-0.933520000	3.648295000	-2.494114000
H	-0.392146000	4.611142000	-2.413911000
H	-0.994850000	3.348887000	-3.557891000
H	-1.948473000	3.764376000	-2.085506000
C	1.711425000	2.840653000	-3.057646000
H	1.282812000	3.643528000	-3.663284000
C	3.016866000	2.395081000	-3.317419000
H	3.588666000	2.859213000	-4.129110000
C	3.580055000	1.366107000	-2.552603000
H	4.592746000	1.006428000	-2.760597000
C	2.830835000	0.779480000	-1.521661000
H	3.262987000	-0.041170000	-0.944059000
C	2.546032000	-0.266030000	1.980931000
H	2.430782000	0.776107000	2.296985000
C	3.470341000	-1.089929000	2.641900000
H	4.068502000	-0.680970000	3.464162000
C	3.622880000	-2.427449000	2.247206000
H	4.339553000	-3.077970000	2.761718000
C	2.857540000	-2.932535000	1.185579000
H	2.954496000	-3.969265000	0.847899000
C	1.934259000	-2.110367000	0.520159000
S	1.028317000	-2.844247000	-0.886875000
O	1.338583000	-1.948498000	-2.041987000
O	1.458899000	-4.262760000	-0.965134000
O	-0.451186000	-2.737027000	-0.437040000
C	-2.527655000	0.889392000	-0.596637000
C	-3.920213000	0.415218000	-0.124243000
H	-3.921116000	0.291992000	0.980351000
H	-4.737887000	1.133631000	-0.348498000
C	-4.222161000	-0.952461000	-0.683974000
H	-2.546727000	1.165891000	-1.667168000
H	-2.179749000	1.754154000	-0.012139000
O	-3.267188000	-1.719961000	-0.932323000
C	-5.638796000	-1.405434000	-0.938226000
H	-6.268940000	-1.228531000	-0.046778000
H	-6.075776000	-0.803226000	-1.758066000
H	-5.661971000	-2.470102000	-1.214953000

TS-$1_{Coor\text{-}E\text{-}T}$

Zero-point correction= 0.486139 (Hartree/Particle)
Thermal correction to Energy= 0.523721
Thermal correction to Enthalpy= 0.524665
Thermal correction to Gibbs Free Energy= 0.415373
Sum of electronic and zero-point Energies= -2325.407244
Sum of electronic and thermal Energies= -2325.369662
Sum of electronic and thermal Enthalpies= -2325.368717
Sum of electronic and thermal Free Energies= -2325.478010
E solvent= -2325.26243405

Pd	1.195715000	-0.838545000	0.427986000
P	-0.591970000	0.459248000	-0.101082000
C	-1.865043000	0.775542000	1.206416000
C	-0.184134000	2.146929000	-0.752020000
C	-1.569227000	-0.282812000	-1.498642000
C	0.721652000	2.272123000	-1.845943000
O	1.201203000	1.096138000	-2.360888000
C	1.969961000	1.143237000	-3.566751000
H	2.926549000	1.682935000	-3.420705000
H	1.401294000	1.622328000	-4.386172000
H	2.175519000	0.093439000	-3.821822000
C	1.064410000	3.542652000	-2.347708000
H	1.753716000	3.637187000	-3.191518000
C	0.512619000	4.696594000	-1.766769000
H	0.785643000	5.679795000	-2.166889000
C	-0.383151000	4.592438000	-0.694824000
H	-0.824003000	5.489983000	-0.248414000
C	-0.725089000	3.322642000	-0.199252000
H	-1.441171000	3.240834000	0.623997000
C	-1.506405000	1.355654000	2.455412000
O	-0.196546000	1.745584000	2.587194000
C	0.220841000	2.287564000	3.841984000
H	-0.305130000	3.234987000	4.070721000
H	0.056917000	1.568565000	4.667305000
H	1.298085000	2.483677000	3.733888000
C	-2.471382000	1.525267000	3.467154000
H	-2.196576000	1.972681000	4.426184000
C	-3.795187000	1.113350000	3.247339000
H	-4.535328000	1.244597000	4.044954000
C	-4.164392000	0.536498000	2.025857000
H	-5.192878000	0.203271000	1.854327000
C	-3.200300000	0.367678000	1.020691000
H	-3.486084000	-0.105859000	0.077947000
C	-2.148158000	0.566688000	-2.467300000
H	-2.029891000	1.651128000	-2.372587000
C	-2.876030000	0.045260000	-3.548017000
H	-3.318184000	0.728315000	-4.282366000
C	-3.034479000	-1.342055000	-3.680545000
H	-3.597679000	-1.757372000	-4.524193000

C	-2.474536000	-2.200552000	-2.723188000
H	-2.584563000	-3.287456000	-2.790518000
C	-1.750220000	-1.681969000	-1.637557000
S	-1.143562000	-2.893284000	-0.410883000
O	-1.788887000	-2.478358000	0.874419000
O	-1.483599000	-4.234755000	-0.948760000
O	0.388141000	-2.676831000	-0.402369000
C	2.300868000	0.749945000	1.104897000
C	3.579659000	0.776399000	0.247904000
H	3.324403000	1.125655000	-0.775142000
H	4.346771000	1.483144000	0.631626000
C	4.148538000	-0.613434000	0.073573000
H	2.523559000	0.481799000	2.154834000
H	1.789631000	1.722649000	1.080949000
O	3.372821000	-1.587592000	0.071395000
C	5.637469000	-0.815490000	-0.104759000
H	6.030792000	-0.142556000	-0.889625000
H	6.163072000	-0.546887000	0.832006000
H	5.859005000	-1.863045000	-0.359002000
C	1.849978000	-2.761633000	2.564958000
C	0.807904000	-1.967258000	2.896358000
H	1.724094000	-3.598417000	1.869561000
H	2.858282000	-2.597301000	2.965436000
H	-0.202313000	-2.157210000	2.512793000
H	0.928063000	-1.135006000	3.601902000

1-Coor-E-T

Zero-point correction= 0.487844 (Hartree/Particle)
Thermal correction to Energy= 0.525574
Thermal correction to Enthalpy= 0.526518
Thermal correction to Gibbs Free Energy= 0.415726
Sum of electronic and zero-point Energies= -2325.414618
Sum of electronic and thermal Energies= -2325.376888
Sum of electronic and thermal Enthalpies= -2325.375944
Sum of electronic and thermal Free Energies= -2325.486736
E solvent= -2325.26821645

Pd	-0.147328000	-1.601701000	-0.808324000
P	0.367740000	0.582349000	0.128301000
C	0.513828000	2.045290000	-0.998358000
C	-0.753892000	1.100929000	1.503970000
C	2.011637000	0.596538000	1.017372000
C	-1.020626000	0.135429000	2.516359000
O	-0.394749000	-1.070911000	2.347834000
C	-0.566148000	-2.081527000	3.346696000
H	-1.626834000	-2.386966000	3.433323000
H	-0.199267000	-1.734348000	4.331087000
H	0.039172000	-2.932401000	3.003221000
C	-1.864675000	0.447813000	3.599163000
H	-2.068506000	-0.292950000	4.377629000
C	-2.436513000	1.728672000	3.684210000
H	-3.089302000	1.966495000	4.531821000
C	-2.171163000	2.695686000	2.704968000
H	-2.609225000	3.696409000	2.780076000
C	-1.331915000	2.376953000	1.623369000
H	-1.109895000	3.135147000	0.866731000
C	-0.614341000	2.590643000	-1.674769000
O	-1.826847000	2.020561000	-1.388796000
C	-2.986251000	2.467366000	-2.111118000
H	-3.244293000	3.508950000	-1.839620000
H	-2.822918000	2.399476000	-3.203206000
H	-3.790045000	1.777582000	-1.813893000
C	-0.456852000	3.670666000	-2.565460000
H	-1.327683000	4.102214000	-3.065761000
C	0.821592000	4.191687000	-2.817289000
H	0.930575000	5.026172000	-3.519420000
C	1.945501000	3.646935000	-2.183776000
H	2.947031000	4.038487000	-2.388357000
C	1.785101000	2.583032000	-1.283187000
H	2.666839000	2.151318000	-0.803787000
C	2.190390000	1.492719000	2.094282000
H	1.368287000	2.153993000	2.386810000
C	3.403772000	1.552731000	2.796671000
H	3.514739000	2.262258000	3.624483000
C	4.464507000	0.708444000	2.436464000
H	5.413860000	0.746650000	2.982831000
C	4.307707000	-0.186600000	1.368694000
H	5.112725000	-0.860090000	1.058410000
C	3.095310000	-0.241885000	0.661609000
S	3.004015000	-1.412717000	-0.728234000
O	2.623579000	-0.589155000	-1.919212000
O	4.302450000	-2.131492000	-0.765253000
O	1.851026000	-2.357733000	-0.294896000
C	-2.121498000	-1.152353000	-1.246129000
C	-3.025570000	-1.394154000	-0.040200000
H	-2.979305000	-2.441645000	0.317377000
H	-2.705389000	-0.769231000	0.819661000
C	-4.503099000	-1.019883000	-0.274741000
H	-2.417090000	-1.804980000	-2.087888000
H	-2.165321000	-0.107941000	-1.576697000
O	-4.857962000	-0.176741000	-1.095301000
C	-5.519253000	-1.751160000	0.599133000
H	-5.566839000	-2.817087000	0.303946000
H	-5.213192000	-1.728914000	1.661797000
H	-6.516341000	-1.298818000	0.483971000
C	-0.028383000	-2.900123000	-2.600948000
C	-0.466445000	-3.684796000	-1.537534000
H	-0.727949000	-2.505635000	-3.346684000
H	1.037574000	-2.816632000	-2.838965000
H	-1.525994000	-3.942858000	-1.424289000
H	0.255009000	-4.248743000	-0.936166000

TS_{Isom}

Zero-point correction= 0.487002 (Hartree/Particle)
Thermal correction to Energy= 0.524038
Thermal correction to Enthalpy= 0.524982
Thermal correction to Gibbs Free Energy= 0.415843
Sum of electronic and zero-point Energies= -2325.398488
Sum of electronic and thermal Energies= -2325.361452
Sum of electronic and thermal Enthalpies= -2325.360508
Sum of electronic and thermal Free Energies= -2325.469647
E solvent= -2325.24806751

C	2.376486000	2.636215000	-0.149222000
C	1.124809000	2.000298000	0.000550000
C	-0.042290000	2.800684000	-0.074058000
C	0.048132000	4.185772000	-0.287986000
C	1.302438000	4.797547000	-0.428693000
C	2.468808000	4.021159000	-0.357521000
P	1.029628000	0.155232000	0.230946000
Pd	-1.095281000	-0.889702000	-0.873780000
O	-1.877724000	1.137611000	-1.079216000
S	-1.721603000	2.115838000	0.123303000
O	-1.708065000	1.386857000	1.433209000
C	2.547913000	-0.406866000	-0.664871000
C	2.458094000	-0.520167000	-2.079655000
C	3.556212000	-0.965827000	-2.837490000
C	4.754701000	-1.303643000	-2.184970000
C	4.861294000	-1.202296000	-0.790965000
C	3.758907000	-0.756047000	-0.041377000

O	1.244545000	-0.160243000	-2.619459000
C	1.117215000	-0.114266000	-4.045658000
C	1.437957000	-0.075772000	2.014534000
C	1.586040000	-1.392737000	2.532959000
C	1.804837000	-1.598504000	3.908110000
C	1.856370000	-0.496765000	4.778498000
C	1.693437000	0.804852000	4.287422000
C	1.484383000	1.006356000	2.913643000
O	1.513748000	-2.407658000	1.608650000
C	1.671359000	-3.748816000	2.078752000
H	-0.880806000	4.761845000	-0.340048000
H	1.366029000	5.879069000	-0.594229000
H	3.454010000	4.489378000	-0.464404000
H	3.290605000	2.034098000	-0.105227000
H	1.928016000	-2.608933000	4.307248000
H	2.020662000	-0.668740000	5.848320000
H	1.722867000	1.663624000	4.965928000
H	1.344774000	2.021288000	2.530365000
H	3.837905000	-0.668312000	1.047908000
H	5.797550000	-1.463218000	-0.286033000
H	5.608019000	-1.647400000	-2.780593000
H	3.487524000	-1.048010000	-3.925906000
C	-0.483939000	-2.915487000	-1.084277000
H	0.099735000	0.257068000	-4.235054000
H	1.855752000	0.581956000	-4.485448000
H	1.241156000	-1.117585000	-4.497400000
H	1.583962000	-4.388416000	1.187879000
H	2.665332000	-3.899904000	2.542684000
H	0.882413000	-4.019011000	2.806993000
H	-0.750632000	-3.489663000	-0.188670000
C	-1.462387000	-2.638131000	-2.072065000
H	0.584829000	-2.904568000	-1.329435000
H	-2.487302000	-3.010905000	-1.970934000
H	-1.163979000	-2.369141000	-3.093387000
C	-2.902511000	-1.345580000	0.051575000
H	-2.895072000	-2.425205000	0.274434000
H	-2.676873000	-0.742329000	0.948423000
O	-2.662752000	3.255269000	-0.019904000
C	-4.145692000	-0.893436000	-0.689244000
H	-3.993772000	0.129999000	-1.075775000
H	-4.401457000	-1.576353000	-1.516639000
C	-5.341941000	-0.891753000	0.305568000
O	-6.112810000	-1.842583000	0.363949000
C	-5.477188000	0.346509000	1.176733000
H	-4.500364000	0.661668000	1.587490000
H	-5.830805000	1.190171000	0.552720000
H	-6.202155000	0.168098000	1.986042000

1-Coor-E-C

Zero-point correction= 0.487244 (Hartree/Particle)
Thermal correction to Energy= 0.525127
Thermal correction to Enthalpy= 0.526071
Thermal correction to Gibbs Free Energy= 0.412367
Sum of electronic and zero-point Energies= -2325.412595
Sum of electronic and thermal Energies= -2325.374712
Sum of electronic and thermal Enthalpies= -2325.373768
Sum of electronic and thermal Free Energies= -2325.487471
E solvent= -2325.26401593

Pd	1.334455000	-0.696186000	0.196158000
P	-1.092168000	0.090941000	-0.070457000
C	-2.017927000	-0.067924000	-1.665437000
C	-2.230864000	-0.652463000	1.189093000
C	-1.259069000	1.905350000	0.323587000
C	-1.769428000	-0.704087000	2.535568000
O	-0.548012000	-0.125918000	2.755760000
C	-0.018415000	-0.126928000	4.085754000
H	0.128744000	-1.157878000	4.460763000
H	-0.679359000	0.428646000	4.777990000
H	0.952842000	0.382872000	4.012384000
C	-2.555658000	-1.299219000	3.540757000
H	-2.199684000	-1.340336000	4.573922000
C	-3.813124000	-1.833620000	3.213274000
H	-4.420519000	-2.292046000	4.002065000
C	-4.291520000	-1.775808000	1.897071000
H	-5.277449000	-2.181332000	1.646114000
C	-3.498757000	-1.187149000	0.896521000
H	-3.875790000	-1.130736000	-0.129978000
C	-2.253090000	-1.351212000	-2.230664000
O	-1.816042000	-2.424856000	-1.484550000
C	-2.106461000	-3.739892000	-1.969750000
H	-3.197792000	-3.902309000	-2.056987000
H	-1.627754000	-3.926052000	-2.950407000
H	-1.689429000	-4.430294000	-1.221741000
C	-2.905826000	-1.481339000	-3.470551000
H	-3.097976000	-2.469336000	-3.897397000
C	-3.301447000	-0.331587000	-4.173886000
H	-3.800661000	-0.443078000	-5.143052000
C	-3.053978000	0.941456000	-3.645786000
H	-3.349925000	1.840101000	-4.196682000
C	-2.416674000	1.064337000	-2.400853000
H	-2.217337000	2.059504000	-1.993900000
C	-2.436554000	2.399726000	0.924000000
H	-3.257857000	1.709467000	1.144845000
C	-2.570861000	3.760519000	1.242706000
H	-3.498865000	4.119742000	1.701943000
C	-1.521976000	4.652784000	0.973078000
H	-1.620997000	5.715406000	1.222239000
C	-0.340806000	4.181006000	0.381388000
H	0.497812000	4.847849000	0.157818000
C	-0.212386000	2.821099000	0.059804000
S	1.339033000	2.281966000	-0.715880000
O	0.934646000	1.514604000	-1.934568000
O	2.188565000	3.485630000	-0.886220000
O	1.953526000	1.339611000	0.373835000
C	3.318757000	-1.086075000	0.664692000
C	4.257570000	-0.591976000	-0.424034000
H	4.025315000	-1.055059000	-1.407732000
H	4.139222000	0.496194000	-0.577932000
C	5.741208000	-0.909945000	-0.148266000
H	3.462722000	-2.157734000	0.882203000
H	3.429710000	-0.502493000	1.596229000
O	6.097855000	-1.734287000	0.688615000
C	6.747038000	-0.130299000	-0.992182000
H	6.453003000	-0.115104000	-2.057811000
H	6.767998000	0.924055000	-0.655565000
H	7.752786000	-0.564501000	-0.880092000
C	1.275628000	-2.666571000	-0.679799000
C	0.781467000	-2.745688000	0.631289000
H	0.585625000	-2.583543000	-1.525426000
H	2.300889000	-2.974100000	-0.913519000
H	-0.297348000	-2.752107000	0.821517000
H	1.419735000	-3.102058000	1.448672000

TS-$1_{Ins\text{-}E\text{-}C}$

Zero-point correction= 0.487988 (Hartree/Particle)
Thermal correction to Energy= 0.524788
Thermal correction to Enthalpy= 0.525732
Thermal correction to Gibbs Free Energy= 0.416664

Sum of electronic and zero-point Energies= -2325.390878
Sum of electronic and thermal Energies= -2325.354078
Sum of electronic and thermal Enthalpies= -2325.353134
Sum of electronic and thermal Free Energies= -2325.462202
E solvent= -2325.24320459

Pd	-1.101563000	-0.119328000	-1.017966000
P	0.881760000	-0.061947000	0.208895000
C	0.856459000	-0.814026000	1.903413000
C	2.345602000	-0.807962000	-0.641142000
C	1.486897000	1.669770000	0.542934000
C	2.565413000	-0.472704000	-2.007864000
O	1.680282000	0.420571000	-2.544628000
C	1.854781000	0.826717000	-3.905408000
H	1.766265000	-0.032243000	-4.598264000
H	2.833838000	1.321321000	-4.053149000
H	1.044760000	1.544246000	-4.099943000
C	3.648167000	-1.032835000	-2.714010000
H	3.813865000	-0.781272000	-3.765219000
C	4.529420000	-1.908032000	-2.058483000
H	5.371792000	-2.333979000	-2.615551000
C	4.343858000	-2.226017000	-0.705969000
H	5.040671000	-2.896216000	-0.191565000
C	3.255271000	-1.675002000	-0.008971000
H	3.113912000	-1.913382000	1.049916000
C	0.700616000	-2.215516000	2.100317000
O	0.666926000	-2.988292000	0.965108000
C	0.558992000	-4.406604000	1.119758000
H	1.419738000	-4.817172000	1.682039000
H	-0.381787000	-4.688689000	1.630260000
H	0.559157000	-4.815370000	0.098452000
C	0.608159000	-2.748295000	3.400826000
H	0.501401000	-3.825827000	3.551657000
C	0.642930000	-1.892912000	4.513356000
H	0.562070000	-2.320145000	5.519392000
C	0.773601000	-0.510042000	4.337784000
H	0.788623000	0.163508000	5.200605000
C	0.877000000	0.016831000	3.041229000
H	0.959174000	1.098380000	2.908586000
C	2.874326000	1.893322000	0.681823000
H	3.566839000	1.050729000	0.584594000
C	3.382578000	3.175577000	0.940020000
H	4.464035000	3.317575000	1.046791000
C	2.507001000	4.264806000	1.058778000
H	2.896157000	5.270160000	1.256337000
C	1.125851000	4.064554000	0.921946000
H	0.414239000	4.892129000	1.002896000
C	0.615969000	2.780590000	0.668162000
S	-1.196922000	2.636410000	0.529802000
O	-1.609220000	1.655948000	1.586533000
O	-1.738993000	4.015970000	0.623503000
O	-1.388872000	2.053573000	-0.894571000
C	-3.191537000	0.072679000	-1.975290000
C	-4.014775000	0.291745000	-0.709537000
H	-3.421625000	0.746521000	0.107959000
H	-4.786200000	1.063745000	-0.941058000
C	-4.784294000	-0.907323000	-0.161874000
H	-3.797981000	-0.395829000	-2.760881000
H	-2.774510000	1.014480000	-2.367222000
O	-4.886572000	-1.977691000	-0.764993000
C	-5.432145000	-0.700850000	1.201810000
H	-4.641449000	-0.653451000	1.975071000
H	-5.973907000	0.261424000	1.250002000
H	-6.114931000	-1.534189000	1.428267000
C	-0.982070000	-2.123138000	-1.521378000
C	-2.166911000	-1.793929000	-2.267600000
H	-0.035079000	-2.266408000	-2.060152000
H	-1.090535000	-2.736993000	-0.620390000
H	-2.060924000	-1.626944000	-3.346650000
H	-3.116731000	-2.239435000	-1.952038000

1-β-T

Zero-point correction= 0.488575 (Hartree/Particle)
Thermal correction to Energy= 0.525982
Thermal correction to Enthalpy= 0.526926
Thermal correction to Gibbs Free Energy= 0.415040
Sum of electronic and zero-point Energies= -2325.427286
Sum of electronic and thermal Energies= -2325.389879
Sum of electronic and thermal Enthalpies= -2325.388935
Sum of electronic and thermal Free Energies= -2325.500821
E solvent= -2325.28430473

Pd	0.610706000	0.401351000	-1.210252000
P	-1.016030000	-0.240292000	0.210239000
C	-2.574368000	-0.925712000	-0.499152000
C	-0.466237000	-1.447247000	1.492623000
C	-1.575173000	1.219721000	1.215402000
C	0.756370000	-1.200534000	2.184214000
O	1.387519000	-0.029171000	1.878045000
C	2.569837000	0.331514000	2.611453000
H	3.417781000	-0.335546000	2.370224000
H	2.375285000	0.328255000	3.700370000
H	2.808278000	1.353351000	2.282872000
C	1.234800000	-2.129626000	3.129702000
H	2.182357000	-1.953430000	3.646114000
C	0.490152000	-3.285892000	3.411501000
H	0.871176000	-3.998622000	4.151880000
C	-0.729024000	-3.526712000	2.762036000
H	-1.315992000	-4.421658000	2.993943000
C	-1.195388000	-2.609423000	1.806429000
H	-2.148591000	-2.790367000	1.300253000
C	-2.578817000	-2.145314000	-1.232855000
O	-1.381800000	-2.811631000	-1.302089000
C	-1.339160000	-4.043994000	-2.026772000
H	-2.008866000	-4.801907000	-1.576220000
H	-1.611987000	-3.898236000	-3.089573000
H	-0.297060000	-4.389619000	-1.958260000
C	-3.767622000	-2.615914000	-1.823078000
H	-3.775399000	-3.556836000	-2.379579000
C	-4.950188000	-1.868357000	-1.706144000
H	-5.866859000	-2.241709000	-2.176590000
C	-4.957288000	-0.656165000	-1.004547000
H	-5.874086000	-0.063505000	-0.924510000
C	-3.773515000	-0.191633000	-0.412241000
H	-3.775069000	0.766249000	0.113804000
C	-2.005039000	0.996957000	2.542394000
H	-2.003548000	-0.021337000	2.945749000
C	-2.431498000	2.060017000	3.353114000
H	-2.763851000	1.859606000	4.378012000
C	-2.427300000	3.368839000	2.848991000
H	-2.753466000	4.205350000	3.477543000
C	-2.004272000	3.607575000	1.533043000
H	-1.989890000	4.616120000	1.108075000
C	-1.581563000	2.546448000	0.716023000
S	-1.108552000	2.976581000	-1.000203000
O	-1.992584000	2.136683000	-1.864415000
O	-1.224550000	4.452065000	-1.106986000
O	0.379288000	2.530172000	-1.069430000
C	3.809476000	-0.859287000	-1.063458000
C	5.009224000	0.045813000	-1.367193000
H	5.546647000	-0.268844000	-2.284966000
H	4.665721000	1.082504000	-1.578431000
C	6.009662000	0.145423000	-0.208637000

H	4.147761000	-1.909381000	-0.975123000
H	3.401230000	-0.583770000	-0.075922000
O	5.746421000	-0.271665000	0.917796000
C	7.348918000	0.802551000	-0.524985000
H	7.948471000	0.134318000	-1.172974000
H	7.209305000	1.745844000	-1.084626000
H	7.906092000	0.993996000	0.404836000
C	1.377934000	-1.432326000	-1.646101000
C	2.675680000	-0.781860000	-2.101546000
H	1.472438000	-2.099622000	-0.775408000
H	0.774779000	-1.893357000	-2.443424000
H	2.991749000	-1.148476000	-3.100807000
H	2.482448000	0.326752000	-2.320653000

TS-1$_{Ins\text{-}E\text{-}T}$

Zero-point correction= 0.487435 (Hartree/Particle)
Thermal correction to Energy= 0.524389
Thermal correction to Enthalpy= 0.525333
Thermal correction to Gibbs Free Energy= 0.417222
Sum of electronic and zero-point Energies= -2325.373269
Sum of electronic and thermal Energies= -2325.336314
Sum of electronic and thermal Enthalpies= -2325.335370
Sum of electronic and thermal Free Energies= -2325.443482
E solvent= -2325.22979894

Pd	0.114999000	-1.430790000	-1.133527000
P	-0.392874000	0.584705000	0.184337000
C	0.925214000	1.218590000	1.325604000
C	-0.956189000	2.075246000	-0.764558000
C	-1.843697000	0.363275000	1.341278000
C	-1.880530000	1.860269000	-1.824593000
O	-2.268650000	0.562991000	-1.998511000
C	-3.247552000	0.256971000	-2.994685000
H	-2.876557000	0.492667000	-4.011178000
H	-4.191277000	0.805155000	-2.809802000
H	-3.422130000	-0.824097000	-2.899148000
C	-2.347165000	2.940861000	-2.597856000
H	-3.051551000	2.771987000	-3.417182000
C	-1.909813000	4.243222000	-2.305971000
H	-2.279653000	5.079434000	-2.910492000
C	-1.017265000	4.475946000	-1.250356000
H	-0.686360000	5.493447000	-1.016331000
C	-0.547484000	3.392191000	-0.488418000
H	0.144946000	3.574262000	0.339851000
C	2.108221000	1.818270000	0.804026000
O	2.125826000	2.021803000	-0.554145000
C	3.307517000	2.589538000	-1.143477000
H	3.516612000	3.592042000	-0.724518000
H	4.176135000	1.920835000	-1.006101000
H	3.076571000	2.684626000	-2.214814000
C	3.166755000	2.178449000	1.660801000
H	4.071281000	2.640852000	1.257399000
C	3.066626000	1.927580000	3.039825000
H	3.897232000	2.211009000	3.696410000
C	1.920288000	1.318811000	3.568318000
H	1.841754000	1.115192000	4.641338000
C	0.862831000	0.970304000	2.711777000
H	-0.028578000	0.489810000	3.125969000
C	-2.409803000	1.515168000	1.931866000
H	-1.984761000	2.498267000	1.701959000
C	-3.508141000	1.427123000	2.799442000
H	-3.920985000	2.338415000	3.246795000
C	-4.076367000	0.175295000	3.080808000
H	-4.940835000	0.096491000	3.749885000
C	-3.539418000	-0.979411000	2.495360000
H	-3.969016000	-1.968675000	2.680886000
C	-2.430354000	-0.890975000	1.636347000
S	-1.810191000	-2.462904000	0.953547000
O	-0.422147000	-2.628143000	1.489824000
O	-2.816416000	-3.499154000	1.293571000
O	-1.810150000	-2.188602000	-0.585719000
C	2.359257000	-1.063403000	-1.482160000
C	2.956733000	-1.646940000	-0.207100000
H	2.293677000	-1.507185000	0.668531000
H	3.074604000	-2.749744000	-0.282453000
C	4.345118000	-1.079786000	0.128544000
H	2.039849000	-0.014423000	-1.380827000
H	3.108529000	-1.038412000	-2.285711000
O	4.965436000	-0.351341000	-0.646342000
C	4.903172000	-1.457478000	1.494771000
H	4.395409000	-0.843683000	2.263969000
H	4.702882000	-2.515087000	1.743381000
H	5.984355000	-1.251796000	1.533024000
C	0.303082000	-3.086307000	-2.370573000
C	1.582081000	-2.532059000	-2.700578000
H	0.245175000	-3.976844000	-1.731713000
H	-0.538190000	-2.948670000	-3.063232000
H	2.466373000	-3.129142000	-2.446053000
H	1.670865000	-1.989420000	-3.649500000

Alternating pathway

TS-1$_{Coor\text{-}CO\text{-}T}$

Zero-point correction= 0.440961 (Hartree/Particle)
Thermal correction to Energy= 0.477703
Thermal correction to Enthalpy= 0.478647
Thermal correction to Gibbs Free Energy= 0.371341
Sum of electronic and zero-point Energies= -2360.183397
Sum of electronic and thermal Energies= -2360.146655
Sum of electronic and thermal Enthalpies= -2360.145711
Sum of electronic and thermal Free Energies= -2360.253017
E solvent= -2360.00746352

Pd	-1.477603000	-0.172352000	0.130714000
P	0.693181000	0.204102000	-0.377969000
C	1.458528000	-0.980714000	-1.574368000
C	1.051124000	1.860938000	-1.117717000
C	1.802566000	0.063090000	1.099613000
C	0.845410000	3.047657000	-0.353788000
O	0.474025000	2.862661000	0.948265000
C	0.379919000	4.012474000	1.796946000
H	-0.414874000	4.702973000	1.454973000
H	1.344472000	4.552788000	1.844337000
H	0.123603000	3.620982000	2.791074000
C	1.030372000	4.314196000	-0.942967000
H	0.888579000	5.223698000	-0.352839000
C	1.388653000	4.413046000	-2.297040000
H	1.523998000	5.404917000	-2.743250000
C	1.567056000	3.258484000	-3.070100000
H	1.841390000	3.331856000	-4.127551000
C	1.397129000	1.996412000	-2.477182000
H	1.544427000	1.094115000	-3.078594000
C	0.737458000	-2.057119000	-2.158547000
O	-0.610609000	-2.111652000	-1.955734000
C	-1.232058000	-3.408892000	-1.944144000
H	-1.326342000	-3.824121000	-2.966237000
H	-0.668862000	-4.093170000	-1.288166000
H	-2.234901000	-3.242589000	-1.523218000
C	1.421276000	-3.010427000	-2.944184000
H	0.869583000	-3.848508000	-3.379160000

C	2.796962000	-2.888144000	-3.172501000
H	3.307797000	-3.638334000	-3.787123000
C	3.518147000	-1.819421000	-2.617445000
H	4.595829000	-1.723820000	-2.785870000
C	2.846165000	-0.885329000	-1.818638000
H	3.414895000	-0.074447000	-1.350194000
C	2.909018000	0.927137000	1.240353000
H	3.091924000	1.698782000	0.484054000
C	3.776921000	0.816254000	2.337340000
H	4.632663000	1.495734000	2.422851000
C	3.542360000	-0.159316000	3.317561000
H	4.212626000	-0.249248000	4.180080000
C	2.444187000	-1.023256000	3.193750000
H	2.230612000	-1.792099000	3.942809000
C	1.576678000	-0.920155000	2.094656000
S	0.212548000	-2.139559000	2.010566000
O	0.448138000	-2.890594000	0.739226000
O	0.263628000	-2.904326000	3.281437000
O	-1.062735000	-1.260942000	1.952771000
C	-2.157088000	0.799017000	-1.547073000
C	-3.609304000	1.207286000	-1.225819000
H	-3.594969000	2.078072000	-0.536702000
H	-4.191836000	1.521685000	-2.117185000
C	-4.315160000	0.122772000	-0.454733000
H	-2.115680000	0.033729000	-2.343641000
H	-1.561237000	1.674150000	-1.850914000
O	-3.641170000	-0.630789000	0.281731000
C	-5.812803000	-0.044632000	-0.535296000
H	-6.316065000	0.921560000	-0.343979000
H	-6.097719000	-0.350661000	-1.560281000
H	-6.159799000	-0.800255000	0.185325000
C	-2.297938000	1.726216000	1.945658000
O	-1.766571000	1.582689000	2.961184000

1-Coor-CO-T

Zero-point correction= 0.442007 (Hartree/Particle)
Thermal correction to Energy= 0.479198
Thermal correction to Enthalpy= 0.480142
Thermal correction to Gibbs Free Energy= 0.369789
Sum of electronic and zero-point Energies= -2360.197167
Sum of electronic and thermal Energies= -2360.159976
Sum of electronic and thermal Enthalpies= -2360.159031
Sum of electronic and thermal Free Energies= -2360.269385
E solvent= -2360.02267069

Pd	0.921923000	-1.287540000	-0.132096000
P	-0.556953000	0.569153000	0.255828000
C	-1.721623000	0.488871000	1.685215000
C	0.364062000	2.150737000	0.490063000
C	-1.674635000	0.921534000	-1.184650000
C	1.214645000	2.589735000	-0.567098000
O	1.205437000	1.801420000	-1.683477000
C	2.006971000	2.185472000	-2.805597000
H	3.081161000	2.221711000	-2.540023000
H	1.691718000	3.168471000	-3.204136000
H	1.839731000	1.406204000	-3.562651000
C	1.992627000	3.754451000	-0.421410000
H	2.629217000	4.102370000	-1.239746000
C	1.949669000	4.472313000	0.786315000
H	2.559648000	5.376697000	0.891408000
C	1.140961000	4.039037000	1.845521000
H	1.112761000	4.596806000	2.787314000
C	0.353292000	2.884622000	1.690970000
H	-0.288405000	2.551425000	2.513167000
C	-1.765414000	-0.640781000	2.548156000
O	-0.799584000	-1.586977000	2.391723000
C	-1.082230000	-2.922731000	2.836953000
H	-1.061968000	-2.995474000	3.941567000
H	-2.055438000	-3.262341000	2.442853000
H	-0.278780000	-3.543598000	2.414735000
C	-2.772393000	-0.729980000	3.533696000
H	-2.817988000	-1.605526000	4.187090000
C	-3.708748000	0.300349000	3.683784000
H	-4.481046000	0.213834000	4.456695000
C	-3.665415000	1.430767000	2.852753000
H	-4.401653000	2.233765000	2.961785000
C	-2.682157000	1.508888000	1.857440000
H	-2.674821000	2.368552000	1.178509000
C	-2.021635000	2.248873000	-1.513042000
H	-1.600026000	3.077741000	-0.932988000
C	-2.891225000	2.523997000	-2.580222000
H	-3.152070000	3.562885000	-2.812279000
C	-3.418916000	1.472115000	-3.344270000
H	-4.097289000	1.681091000	-4.179212000
C	-3.071405000	0.147384000	-3.040900000
H	-3.455646000	-0.696498000	-3.622561000
C	-2.206636000	-0.128471000	-1.970775000
S	-1.795080000	-1.872413000	-1.648106000
O	-2.140526000	-2.095717000	-0.208881000
O	-2.505892000	-2.676684000	-2.671326000
O	-0.257966000	-1.901109000	-1.884805000
C	2.099372000	-0.740107000	1.517523000
C	3.301164000	0.134242000	1.187694000
H	3.022633000	1.053797000	0.637135000
H	3.743546000	0.518081000	2.138572000
C	4.457397000	-0.558087000	0.470749000
H	2.421259000	-1.693169000	1.967998000
C	2.089580000	-2.770819000	-0.550336000
H	1.420263000	-0.230413000	2.217271000
O	4.507753000	-1.778798000	0.329059000
C	5.571171000	0.345169000	-0.053566000
H	5.229890000	0.837003000	-0.985431000
H	5.817898000	1.149762000	0.662647000
H	6.467971000	-0.251594000	-0.281053000
O	2.596235000	-3.741999000	-0.917267000

TS-1$_{Ins\text{-}CO\text{-}T}$

Zero-point correction= 0.441912 (Hartree/Particle)
Thermal correction to Energy= 0.478438
Thermal correction to Enthalpy= 0.479382
Thermal correction to Gibbs Free Energy= 0.370286
Sum of electronic and zero-point Energies= -2360.176045
Sum of electronic and thermal Energies= -2360.139519
Sum of electronic and thermal Enthalpies= -2360.138575
Sum of electronic and thermal Free Energies= -2360.247671
E solvent= -2359.99462473

Pd	-0.403863000	-1.467704000	-0.827887000
P	0.658052000	0.552972000	0.165756000
C	0.986944000	2.034662000	-0.881997000
C	-0.291140000	1.215264000	1.609675000
C	2.307379000	0.151197000	0.915849000
C	-0.747863000	0.287601000	2.588348000
O	-0.385855000	-1.015984000	2.369461000
C	-0.736152000	-2.001332000	3.346468000
H	-1.834464000	-2.092150000	3.456399000
H	-0.286520000	-1.766009000	4.329773000
H	-0.324078000	-2.946942000	2.965908000
C	-1.507821000	0.724479000	3.689746000
H	-1.850315000	0.013349000	4.446810000

C	-1.818753000	2.089221000	3.819928000
H	-2.409356000	2.422388000	4.681018000
C	-1.377731000	3.015936000	2.865702000
H	-1.617403000	4.079147000	2.972030000
C	-0.616549000	2.573753000	1.769591000
H	-0.260620000	3.295444000	1.026765000
C	-0.073103000	2.611534000	-1.634692000
O	-1.315620000	2.052799000	-1.456424000
C	-2.415739000	2.604593000	-2.199922000
H	-2.585073000	3.663128000	-1.925455000
H	-2.234239000	2.529258000	-3.288726000
H	-3.292116000	2.000727000	-1.921241000
C	0.173907000	3.703621000	-2.488475000
H	-0.639376000	4.152730000	-3.064071000
C	1.476274000	4.214376000	-2.610032000
H	1.657018000	5.059708000	-3.283679000
C	2.533171000	3.648291000	-1.885913000
H	3.550877000	4.038764000	-1.987716000
C	2.282299000	2.564222000	-1.030292000
H	3.108994000	2.112370000	-0.474566000
C	2.755806000	0.828489000	2.069508000
H	2.128425000	1.610895000	2.510735000
C	3.992183000	0.514443000	2.656078000
H	4.323738000	1.059700000	3.547067000
C	4.797432000	-0.491414000	2.100137000
H	5.762793000	-0.741186000	2.554780000
C	4.365531000	-1.180823000	0.956806000
H	4.969059000	-1.971313000	0.499791000
C	3.131835000	-0.862536000	0.368979000
S	2.640291000	-1.790396000	-1.118620000
O	2.257624000	-0.754604000	-2.126401000
O	3.746634000	-2.725038000	-1.431005000
O	1.383830000	-2.590717000	-0.626698000
C	-2.640922000	-1.049174000	-1.225759000
C	-3.667308000	-1.300662000	-0.125222000
H	-4.048429000	-2.341250000	-0.151479000
H	-3.203035000	-1.178953000	0.873797000
C	-4.871344000	-0.347071000	-0.192116000
H	-3.055622000	-1.128494000	-2.242383000
C	-1.708479000	-2.688734000	-1.379321000
H	-2.208369000	-0.034321000	-1.148748000
O	-4.915497000	0.606588000	-0.965458000
C	-6.021739000	-0.661851000	0.757186000
H	-5.655444000	-0.837007000	1.785427000
H	-6.749954000	0.163371000	0.751856000
H	-6.527602000	-1.592233000	0.435203000
O	-2.284284000	-3.684729000	-1.640533000

1-cycle6-C

Zero-point correction= 0.443897 (Hartree/Particle)
Thermal correction to Energy= 0.480371
Thermal correction to Enthalpy= 0.481315
Thermal correction to Gibbs Free Energy= 0.371508
Sum of electronic and zero-point Energies= -2360.194759
Sum of electronic and thermal Energies= -2360.158286
Sum of electronic and thermal Enthalpies= -2360.157342
Sum of electronic and thermal Free Energies= -2360.267149
E solvent= -2360.0144873

Pd	-1.294008000	-1.022874000	-0.225105000
P	0.783209000	0.459673000	0.061472000
C	1.550535000	1.457191000	-1.294094000
C	0.590795000	1.682941000	1.439217000
C	2.198435000	-0.589934000	0.654484000
C	-0.004181000	1.226697000	2.649644000
O	-0.284615000	-0.113347000	2.689553000
C	-0.908232000	-0.650641000	3.858678000
H	-1.883164000	-0.163832000	4.056180000
H	-0.256281000	-0.540818000	4.746676000
H	-1.064032000	-1.717185000	3.641310000
C	-0.254872000	2.120233000	3.707896000
H	-0.705142000	1.766466000	4.639881000
C	0.088067000	3.476403000	3.566705000
H	-0.106899000	4.165834000	4.396438000
C	0.678062000	3.942901000	2.384327000
H	0.951938000	4.998241000	2.279065000
C	0.925754000	3.044048000	1.331365000
H	1.393116000	3.402389000	0.407799000
C	0.723813000	2.314960000	-2.068208000
O	-0.588130000	2.393203000	-1.665350000
C	-1.471778000	3.197910000	-2.442972000
H	-1.170908000	4.264232000	-2.431992000
H	-1.526888000	2.845359000	-3.491419000
H	-2.458959000	3.095550000	-1.968105000
C	1.253285000	3.032330000	-3.157523000
H	0.617423000	3.694375000	-3.751679000
C	2.611079000	2.891190000	-3.488979000
H	3.013906000	3.449021000	-4.342164000
C	3.439186000	2.044091000	-2.742119000
H	4.495615000	1.925374000	-3.004541000
C	2.904408000	1.333704000	-1.654885000
H	3.548049000	0.659227000	-1.082510000
C	3.111713000	-0.108009000	1.616004000
H	2.994975000	0.910845000	2.001973000
C	4.163680000	-0.912482000	2.082851000
H	4.863622000	-0.512725000	2.825726000
C	4.316558000	-2.220089000	1.597076000
H	5.135457000	-2.852554000	1.958621000
C	3.416148000	-2.720040000	0.644207000
H	3.504860000	-3.735315000	0.245171000
C	2.367012000	-1.913077000	0.178113000
S	1.242585000	-2.635705000	-1.061073000
O	1.152507000	-1.631461000	-2.162139000
O	1.760400000	-3.990461000	-1.370479000
O	-0.111095000	-2.761931000	-0.275855000
C	-2.857374000	-2.232108000	-0.305953000
C	-3.934670000	-1.702906000	-1.280266000
H	-3.451515000	-1.359102000	-2.211886000
H	-4.626520000	-2.530545000	-1.517469000
C	-4.722944000	-0.551662000	-0.628629000
H	-5.248501000	-0.919441000	0.278655000
C	-3.898038000	0.651081000	-0.197185000
O	-2.657405000	0.632746000	-0.087146000
O	-3.015834000	-3.221556000	0.375683000
H	-5.525067000	-0.195084000	-1.303553000
C	-4.640329000	1.930650000	0.121572000
H	-5.083108000	2.349339000	-0.802886000
H	-5.482575000	1.729971000	0.809777000
H	-3.957840000	2.668921000	0.568727000

TS-2$_{Coor\text{-}E\text{-}C}$

Zero-point correction= 0.494603 (Hartree/Particle)
Thermal correction to Energy= 0.535139
Thermal correction to Enthalpy= 0.536084
Thermal correction to Gibbs Free Energy= 0.417309
Sum of electronic and zero-point Energies= -2438.721939
Sum of electronic and thermal Energies= -2438.681403
Sum of electronic and thermal Enthalpies= -2438.680459
Sum of electronic and thermal Free Energies= -2438.799233
E solvent= -2438.56532471

Pd	1.268870000	-0.440201000	-0.782503000
P	-0.855860000	0.292363000	0.329557000
C	-0.568589000	1.575257000	1.630636000
C	-2.274309000	0.937856000	-0.668242000
C	-1.711810000	-1.080361000	1.262541000
C	-2.673006000	0.134400000	-1.772878000
O	-1.987467000	-1.037236000	-1.893617000
C	-2.247365000	-1.891743000	-3.009661000
H	-2.052992000	-1.368056000	-3.965205000
H	-3.289872000	-2.264760000	-2.994773000
H	-1.546250000	-2.729955000	-2.892410000
C	-3.708854000	0.556291000	-2.627953000
H	-4.013159000	-0.061449000	-3.477895000
C	-4.359260000	1.776540000	-2.375140000
H	-5.166718000	2.099569000	-3.042521000
C	-3.988835000	2.571936000	-1.281338000
H	-4.504942000	3.517446000	-1.082769000
C	-2.949000000	2.147118000	-0.434301000
H	-2.660126000	2.763979000	0.423372000
C	-0.058701000	2.845170000	1.234735000
O	0.032731000	3.049145000	-0.119116000
C	0.542400000	4.302788000	-0.576007000
H	-0.102579000	5.143312000	-0.253690000
H	1.575539000	4.474709000	-0.216468000
H	0.542089000	4.241196000	-1.674372000
C	0.301758000	3.806451000	2.198810000
H	0.672136000	4.788973000	1.892677000
C	0.178493000	3.503656000	3.565890000
H	0.461334000	4.258644000	4.308586000
C	-0.298958000	2.251887000	3.974304000
H	-0.389346000	2.010959000	5.038738000
C	-0.667451000	1.300203000	3.007821000
H	-1.035700000	0.320902000	3.328561000
C	-2.989050000	-0.811919000	1.805481000
H	-3.417799000	0.188822000	1.680410000
C	-3.720778000	-1.795662000	2.485677000
H	-4.705782000	-1.552452000	2.900179000
C	-3.189886000	-3.087422000	2.618068000
H	-3.755453000	-3.869627000	3.137305000
C	-1.932317000	-3.381546000	2.073928000
H	-1.500904000	-4.384475000	2.146387000
C	-1.190815000	-2.390406000	1.405783000
S	0.450800000	-2.928789000	0.797590000
O	1.449837000	-2.174483000	1.630123000
O	0.465325000	-4.410513000	0.905087000
O	0.483616000	-2.494368000	-0.698583000
C	2.920977000	-1.224268000	-1.505509000
C	3.933705000	-1.490621000	-0.385578000
H	3.390886000	-1.951850000	0.461027000
H	4.698684000	-2.191182000	-0.767146000
C	4.630268000	-0.191264000	0.076138000
H	5.272090000	0.203470000	-0.741936000
C	3.735482000	0.973799000	0.487124000
O	2.589734000	1.161630000	0.039964000
O	3.099756000	-1.446720000	-2.684636000
H	5.319592000	-0.416228000	0.910620000
C	4.313323000	1.966206000	1.472013000
H	4.404923000	1.475061000	2.460376000
H	5.331984000	2.275313000	1.173039000
H	3.656208000	2.843101000	1.568998000
C	1.165730000	1.338303000	-3.314240000
C	-0.059951000	1.399717000	-3.865027000
H	1.434890000	1.959094000	-2.450949000
H	1.945603000	0.680880000	-3.713115000
H	-0.849003000	2.043572000	-3.460473000
H	-0.325752000	0.791626000	-4.738420000

2-Coor-E-C

Zero-point correction= 0.496995 (Hartree/Particle)
Thermal correction to Energy= 0.536934
Thermal correction to Enthalpy= 0.537879
Thermal correction to Gibbs Free Energy= 0.421437
Sum of electronic and zero-point Energies= -2438.756016
Sum of electronic and thermal Energies= -2438.716077
Sum of electronic and thermal Enthalpies= -2438.715133
Sum of electronic and thermal Free Energies= -2438.831575
E solvent= -2438.59613946

Pd	1.233246000	-0.112838000	0.076237000
P	-1.357749000	-0.073093000	-0.037863000
C	-2.339222000	-0.540471000	-1.533899000
C	-2.099495000	-1.086275000	1.322526000
C	-2.000147000	1.626748000	0.371963000
C	-1.479998000	-1.011711000	2.602933000
O	-0.443857000	-0.120550000	2.700579000
C	0.286967000	-0.049875000	3.930822000
H	0.711395000	-1.034775000	4.202576000
H	-0.355030000	0.319131000	4.753426000
H	1.102782000	0.662445000	3.744082000
C	-1.941967000	-1.807093000	3.668918000
H	-1.466433000	-1.749851000	4.651953000
C	-3.031648000	-2.671619000	3.469008000
H	-3.387206000	-3.284377000	4.305380000
C	-3.661757000	-2.748690000	2.219325000
H	-4.515795000	-3.417467000	2.067846000
C	-3.191678000	-1.957139000	1.156952000
H	-3.682681000	-2.012284000	0.179259000
C	-2.189911000	-1.837489000	-2.097335000
O	-1.355088000	-2.694662000	-1.414864000
C	-1.209388000	-4.026888000	-1.917117000
H	-2.177196000	-4.564171000	-1.916060000
H	-0.787932000	-4.030249000	-2.940643000
H	-0.509219000	-4.526841000	-1.231614000
C	-2.877347000	-2.192002000	-3.273071000
H	-2.772256000	-3.193186000	-3.699621000
C	-3.697167000	-1.247416000	-3.912746000
H	-4.221165000	-1.530582000	-4.832711000
C	-3.837505000	0.042165000	-3.385370000
H	-4.466769000	0.784008000	-3.887834000
C	-3.160134000	0.385591000	-2.204434000
H	-3.263246000	1.395568000	-1.797563000
C	-3.198054000	1.784372000	1.101247000
H	-3.760041000	0.895659000	1.408536000
C	-3.678950000	3.059640000	1.438648000
H	-4.615093000	3.154182000	2.000740000
C	-2.963667000	4.204402000	1.055600000
H	-3.333992000	5.202100000	1.317617000
C	-1.768314000	4.070211000	0.333702000
H	-1.184690000	4.941042000	0.019076000
C	-1.291831000	2.794201000	-0.003832000
S	0.264341000	2.701479000	-0.940717000
O	-0.009774000	1.789457000	-2.092784000
O	0.686881000	4.094883000	-1.227040000
O	1.242851000	2.048217000	0.092357000
C	3.185021000	0.109930000	0.517187000
C	3.997697000	0.864729000	-0.530082000
H	3.705647000	0.501196000	-1.529811000
H	3.647851000	1.913044000	-0.470391000
C	5.508879000	0.751376000	-0.303943000
H	5.782685000	1.082916000	0.715913000
C	6.045442000	-0.667581000	-0.517514000

O	5.329475000	-1.582165000	-0.922178000
O	3.607757000	-0.255656000	1.596017000
H	6.048003000	1.429061000	-0.996499000
C	7.517830000	-0.893204000	-0.196270000
H	8.154318000	-0.106042000	-0.640309000
H	7.665290000	-0.843381000	0.899817000
H	7.835379000	-1.884184000	-0.554982000
C	1.312621000	-2.280682000	0.429964000
C	1.834034000	-2.030898000	-0.844033000
H	2.916670000	-2.013794000	-1.019772000
H	1.190085000	-2.099157000	-1.728307000
H	0.272875000	-2.599165000	0.558450000
H	1.985413000	-2.429738000	1.282364000

TS-2$_{Ins-E-C}$

Zero-point correction= 0.496724 (Hartree/Particle)
Thermal correction to Energy= 0.535680
Thermal correction to Enthalpy= 0.536624
Thermal correction to Gibbs Free Energy= 0.422349
Sum of electronic and zero-point Energies= -2438.734222
Sum of electronic and thermal Energies= -2438.695266
Sum of electronic and thermal Enthalpies= -2438.694321
Sum of electronic and thermal Free Energies= -2438.808597
E solvent= -2438.57656923

Pd	-0.923462000	-0.194702000	-0.603461000
P	1.288623000	-0.062634000	0.137537000
C	1.639053000	-0.660944000	1.851287000
C	2.503403000	-0.929205000	-0.950968000
C	1.946177000	1.677859000	0.147064000
C	2.377817000	-0.764852000	-2.360481000
O	1.372707000	0.065467000	-2.770804000
C	1.195917000	0.293797000	-4.173046000
H	0.965709000	-0.647164000	-4.708818000
H	2.094149000	0.763186000	-4.617577000
H	0.341633000	0.981645000	-4.248405000
C	3.265229000	-1.423391000	-3.234592000
H	3.169027000	-1.301721000	-4.317022000
C	4.288692000	-2.230051000	-2.711174000
H	4.976953000	-2.733048000	-3.400166000
C	4.437422000	-2.386386000	-1.325972000
H	5.243486000	-3.006449000	-0.919606000
C	3.544415000	-1.736643000	-0.457569000
H	3.660808000	-1.849866000	0.625267000
C	1.493683000	-2.033174000	2.202348000
O	1.174180000	-2.890411000	1.177668000
C	1.078884000	-4.286344000	1.474652000
H	2.037592000	-4.682943000	1.860995000
H	0.275171000	-4.492276000	2.207733000
H	0.838372000	-4.775298000	0.519048000
C	1.691803000	-2.453062000	3.531899000
H	1.591849000	-3.508108000	3.800581000
C	2.010495000	-1.511877000	4.524004000
H	2.154263000	-1.851263000	5.556142000
C	2.137529000	-0.155646000	4.199373000
H	2.374239000	0.583297000	4.971542000
C	1.949899000	0.258901000	2.871966000
H	2.032925000	1.320253000	2.624350000
C	3.325733000	1.890751000	-0.066180000
H	3.983552000	1.031091000	-0.232304000
C	3.867454000	3.185240000	-0.073608000
H	4.942445000	3.321368000	-0.237664000
C	3.032295000	4.294322000	0.126668000
H	3.446992000	5.308821000	0.118028000
C	1.658923000	4.103159000	0.336977000
H	0.978054000	4.946119000	0.491049000
C	1.115582000	2.808115000	0.349396000
S	-0.681886000	2.674531000	0.636582000
O	-0.834019000	1.778941000	1.831617000
O	-1.192019000	4.064084000	0.753743000
O	-1.190924000	1.990296000	-0.655642000
C	-3.640383000	-0.014371000	0.573527000
C	-5.127373000	0.355008000	0.664704000
H	-5.395264000	0.607980000	1.709868000
H	-5.335312000	1.265970000	0.071563000
C	-6.047980000	-0.768396000	0.184628000
H	-3.014127000	0.761316000	1.057920000
C	-3.172460000	-0.118033000	-0.877489000
H	-3.434115000	-0.962194000	1.098392000
O	-5.606313000	-1.859520000	-0.177852000
C	-7.541373000	-0.469572000	0.166682000
H	-7.874949000	-0.028319000	1.123794000
H	-7.757492000	0.277682000	-0.620891000
H	-8.108879000	-1.388712000	-0.044668000
O	-3.666687000	0.463423000	-1.819044000
C	-2.420088000	-1.777469000	-1.359417000
C	-1.147811000	-2.228013000	-0.789584000
H	-2.478404000	-1.712864000	-2.453505000
H	-3.336010000	-2.203025000	-0.908783000
H	-1.158728000	-2.716648000	0.192198000
H	-0.386900000	-2.622118000	-1.476290000

2-cycle5-T

Zero-point correction= 0.499222 (Hartree/Particle)
Thermal correction to Energy= 0.537994
Thermal correction to Enthalpy= 0.538938
Thermal correction to Gibbs Free Energy= 0.424054
Sum of electronic and zero-point Energies= -2438.767516
Sum of electronic and thermal Energies= -2438.728744
Sum of electronic and thermal Enthalpies= -2438.727800
Sum of electronic and thermal Free Energies= -2438.842683
E solvent= -2438.60864489

Pd	0.849045000	0.165248000	-0.076414000
P	-1.394369000	-0.144840000	0.052147000
C	-2.361183000	-0.496292000	-1.486848000
C	-1.887307000	-1.480398000	1.229645000
C	-2.203728000	1.369220000	0.759859000
C	-1.304540000	-1.495700000	2.530496000
O	-0.468760000	-0.452461000	2.815473000
C	0.164493000	-0.411035000	4.097816000
H	0.797395000	-1.303931000	4.264326000
H	-0.581822000	-0.330244000	4.911240000
H	0.793822000	0.490453000	4.082025000
C	-1.613825000	-2.529564000	3.435913000
H	-1.167260000	-2.543970000	4.433908000
C	-2.510934000	-3.542339000	3.058943000
H	-2.746834000	-4.339369000	3.773453000
C	-3.106162000	-3.532249000	1.790139000
H	-3.814566000	-4.315720000	1.501242000
C	-2.790473000	-2.503323000	0.886991000
H	-3.260748000	-2.487213000	-0.101285000
C	-2.064665000	-1.610632000	-2.319611000
O	-1.064577000	-2.446757000	-1.887698000
C	-0.728768000	-3.568570000	-2.707119000
H	-1.583440000	-4.264385000	-2.815003000
H	-0.386825000	-3.249294000	-3.710243000
H	0.092638000	-4.081096000	-2.183855000
C	-2.792567000	-1.827487000	-3.505883000
H	-2.568402000	-2.685177000	-4.145603000

C	-3.810629000	-0.935608000	-3.876635000
H	-4.364657000	-1.112740000	-4.805544000
C	-4.111108000	0.169984000	-3.071745000
H	-4.896203000	0.875377000	-3.361976000
C	-3.384207000	0.383411000	-1.891294000
H	-3.604289000	1.259875000	-1.276683000
C	-3.270789000	1.229020000	1.673332000
H	-3.622464000	0.227790000	1.944481000
C	-3.888305000	2.355144000	2.239982000
H	-4.717093000	2.220846000	2.944404000
C	-3.443585000	3.641939000	1.901968000
H	-3.919495000	4.525185000	2.343439000
C	-2.386549000	3.798612000	0.992648000
H	-2.016086000	4.787508000	0.704485000
C	-1.767412000	2.675152000	0.423018000
S	-0.433241000	2.983416000	-0.789333000
O	-0.867936000	2.259919000	-2.022455000
O	-0.272688000	4.457653000	-0.864053000
O	0.801436000	2.324475000	-0.123893000
C	5.031127000	-1.049392000	-0.561497000
C	5.837887000	0.251747000	-0.608051000
H	5.969278000	0.623064000	-1.642034000
H	5.274993000	1.053011000	-0.086115000
C	7.201854000	0.121708000	0.080019000
H	5.257303000	-1.714719000	-1.417579000
C	3.535136000	-0.869533000	-0.467744000
H	5.358549000	-1.627822000	0.331715000
O	7.475894000	-0.841676000	0.792815000
C	8.185044000	1.260497000	-0.152346000
H	8.537650000	1.240704000	-1.201622000
H	7.699871000	2.242198000	0.000119000
H	9.050130000	1.157199000	0.520031000
O	3.043128000	0.250534000	-0.203184000
C	1.299390000	-1.821416000	0.132487000
C	2.599189000	-2.035524000	-0.672081000
H	1.484383000	-1.942114000	1.217807000
H	0.509417000	-2.516746000	-0.180274000
H	3.108365000	-2.999169000	-0.453299000
H	2.371408000	-2.050687000	-1.759845000

TS-2$_{Isom}$

Zero-point correction= 0.442034 (Hartree/Particle)
Thermal correction to Energy= 0.478476
Thermal correction to Enthalpy= 0.479420
Thermal correction to Gibbs Free Energy= 0.371109
Sum of electronic and zero-point Energies= -2360.183699
Sum of electronic and thermal Energies= -2360.147257
Sum of electronic and thermal Enthalpies= -2360.146313
Sum of electronic and thermal Free Energies= -2360.254624
E solvent= -2359.99968217

C	2.428848000	2.620567000	-0.102198000
C	1.158855000	2.019192000	0.038212000
C	0.014124000	2.842027000	-0.094599000
C	0.142172000	4.217645000	-0.349439000
C	1.414094000	4.795718000	-0.472258000
C	2.560482000	3.995909000	-0.347662000
P	1.010293000	0.181328000	0.303430000
Pd	-1.141232000	-0.893307000	-0.931110000
O	-1.838088000	1.162836000	-1.073701000
S	-1.688543000	2.206450000	0.076311000
O	-1.744795000	1.561314000	1.427995000
C	2.332496000	-0.428997000	-0.843557000
C	1.975300000	-0.559721000	-2.210535000
C	2.891960000	-1.033630000	-3.161993000
C	4.188186000	-1.389078000	-2.746889000
C	4.560291000	-1.278527000	-1.400396000
C	3.632017000	-0.801209000	-0.458161000
O	0.675000000	-0.184700000	-2.519730000
C	0.297698000	-0.066329000	-3.903459000
C	1.704837000	-0.082186000	1.987673000
C	1.844856000	-1.420636000	2.453898000
C	2.253155000	-1.679531000	3.775157000
C	2.506919000	-0.607111000	4.647211000
C	2.356865000	0.715825000	4.210991000
C	1.956723000	0.970190000	2.888698000
O	1.563341000	-2.394734000	1.528516000
C	1.642348000	-3.760972000	1.945331000
H	-0.770189000	4.813643000	-0.447635000
H	1.507434000	5.869743000	-0.669269000
H	3.558527000	4.438011000	-0.445864000
H	3.326438000	1.996862000	-0.024742000
H	2.369339000	-2.707135000	4.130544000
H	2.819589000	-0.818140000	5.676168000
H	2.545946000	1.551040000	4.893313000
H	1.829772000	2.003405000	2.551114000
H	3.917361000	-0.713691000	0.596397000
H	5.569316000	-1.560132000	-1.080593000
H	4.903470000	-1.759532000	-3.489703000
H	2.608980000	-1.133465000	-4.213687000
C	-1.106456000	-2.715204000	-1.343998000
H	-0.705540000	0.382431000	-3.889933000
H	1.001486000	0.599282000	-4.434508000
H	0.266143000	-1.056220000	-4.395737000
H	1.350702000	-4.355221000	1.066965000
H	2.672668000	-4.030723000	2.247956000
H	0.947186000	-3.968954000	2.781274000
C	-2.754259000	-1.248887000	0.371542000
H	-2.679444000	-2.273321000	0.764748000
H	-2.434035000	-0.491711000	1.107848000
O	-2.594083000	3.355638000	-0.170941000
C	-4.080881000	-0.953290000	-0.293937000
H	-4.025581000	0.007937000	-0.834989000
H	-4.381944000	-1.760525000	-0.981651000
C	-5.174258000	-0.848649000	0.817021000
O	-5.870487000	-1.815316000	1.098941000
C	-5.297028000	0.502644000	1.498726000
H	-4.304002000	0.902967000	1.776544000
H	-5.730716000	1.230974000	0.786214000
H	-5.950263000	0.427173000	2.381707000
O	-1.009608000	-3.855069000	-1.569347000

1-Coor-CO-C

Zero-point correction= 0.442567 (Hartree/Particle)
Thermal correction to Energy= 0.479365
Thermal correction to Enthalpy= 0.480309
Thermal correction to Gibbs Free Energy= 0.371478
Sum of electronic and zero-point Energies= -2360.208626
Sum of electronic and thermal Energies= -2360.171828
Sum of electronic and thermal Enthalpies= -2360.170884
Sum of electronic and thermal Free Energies= -2360.279715
E solvent= -2360.02621874

Pd	-1.456858000	1.111501000	-0.096662000
P	0.746690000	-0.003826000	-0.166438000
C	0.811529000	-1.414212000	-1.347721000
C	2.074240000	1.160359000	-0.690883000
C	1.260940000	-0.628218000	1.501957000
C	2.238271000	2.362543000	0.054247000
O	1.456503000	2.462907000	1.172831000

C	1.486986000	3.682700000	1.921489000
H	1.207654000	4.547806000	1.290230000
H	2.485868000	3.855622000	2.366201000
H	0.744282000	3.552048000	2.721590000
C	3.148627000	3.348247000	-0.371158000
H	3.280442000	4.267892000	0.205590000
C	3.887330000	3.148281000	-1.549416000
H	4.591064000	3.922352000	-1.876092000
C	3.722155000	1.979907000	-2.305973000
H	4.290306000	1.831890000	-3.230287000
C	2.817664000	0.995542000	-1.874049000
H	2.675018000	0.090750000	-2.472735000
C	1.933414000	-2.279611000	-1.492527000
O	3.028202000	-1.990540000	-0.720853000
C	4.144530000	-2.882588000	-0.772635000
H	3.852768000	-3.910753000	-0.485997000
H	4.607087000	-2.897015000	-1.778605000
H	4.868639000	-2.490545000	-0.042943000
C	1.885956000	-3.353415000	-2.403352000
H	2.744246000	-4.021016000	-2.517923000
C	0.730168000	-3.570683000	-3.170403000
H	0.708195000	-4.410899000	-3.873973000
C	-0.383248000	-2.731152000	-3.037170000
H	-1.288495000	-2.904169000	-3.627504000
C	-0.338038000	-1.667345000	-2.123489000
H	-1.219052000	-1.030792000	-1.992070000
C	2.613500000	-0.588591000	1.898598000
H	3.361145000	-0.205834000	1.197291000
C	3.007431000	-1.033158000	3.169129000
H	4.065746000	-0.996639000	3.452467000
C	2.049539000	-1.521576000	4.071175000
H	2.351749000	-1.873297000	5.064113000
C	0.696367000	-1.545146000	3.705011000
H	-0.078303000	-1.896166000	4.393576000
C	0.302474000	-1.095269000	2.434455000
S	-1.485136000	-1.096361000	2.087339000
O	-1.687212000	-1.864777000	0.819340000
O	-2.163897000	-1.577793000	3.313962000
O	-1.793272000	0.427148000	1.873245000
C	-3.415778000	1.909363000	0.080180000
C	-4.362856000	0.719817000	0.109018000
H	-3.355432000	2.420151000	1.054802000
H	-3.654705000	2.625095000	-0.719965000
H	-5.419623000	1.073066000	0.170333000
C	-4.303120000	-0.210807000	-1.107630000
H	-4.203774000	0.117709000	1.022238000
O	-3.733539000	0.100973000	-2.155167000
C	-4.958559000	-1.571126000	-0.929150000
H	-5.130253000	-2.050281000	-1.905611000
H	-4.261529000	-2.195252000	-0.335779000
H	-5.903081000	-1.504800000	-0.359778000
C	-1.391037000	1.907513000	-1.779272000
O	-1.318484000	2.420202000	-2.817876000

TS-1$_{\text{Ins-CO-C}}$

Zero-point correction= 0.441861 (Hartree/Particle)
Thermal correction to Energy= 0.478477
Thermal correction to Enthalpy= 0.479421
Thermal correction to Gibbs Free Energy= 0.368749
Sum of electronic and zero-point Energies= -2360.191268
Sum of electronic and thermal Energies= -2360.154652
Sum of electronic and thermal Enthalpies= -2360.153708
Sum of electronic and thermal Free Energies= -2360.264380
E solvent= -2360.00601038

Pd	-1.199146000	-0.040788000	-0.478209000
P	1.075327000	-0.076087000	0.117486000
C	1.472514000	-0.594991000	1.837594000
C	2.094811000	-1.156576000	-0.972076000
C	1.874066000	1.589020000	-0.061607000
C	1.906199000	-1.063687000	-2.379981000
O	1.010448000	-0.116360000	-2.793386000
C	0.750482000	0.014182000	-4.195777000
H	0.347756000	-0.925785000	-4.618598000
H	1.665138000	0.307298000	-4.745495000
H	-0.002165000	0.811236000	-4.279157000
C	2.628243000	-1.903912000	-3.249110000
H	2.482968000	-1.840049000	-4.330955000
C	3.547245000	-2.826061000	-2.721732000
H	4.105579000	-3.473508000	-3.407499000
C	3.755574000	-2.917770000	-1.338441000
H	4.478265000	-3.632208000	-0.930721000
C	3.027238000	-2.084236000	-0.473625000
H	3.184808000	-2.150123000	0.608037000
C	1.057332000	-1.872695000	2.307214000
O	0.432475000	-2.670808000	1.387544000
C	-0.020528000	-3.962555000	1.808937000
H	0.828694000	-4.606557000	2.108144000
H	-0.736696000	-3.882273000	2.648733000
H	-0.528072000	-4.391216000	0.934000000
C	1.310302000	-2.253532000	3.639481000
H	1.003264000	-3.238121000	4.002040000
C	1.956291000	-1.361235000	4.510031000
H	2.142780000	-1.667578000	5.545687000
C	2.354435000	-0.093324000	4.065927000
H	2.847779000	0.606726000	4.747660000
C	2.108116000	0.282220000	2.736523000
H	2.404806000	1.276807000	2.392062000
C	3.237983000	1.673214000	-0.414333000
H	3.810118000	0.752623000	-0.573525000
C	3.868555000	2.918503000	-0.564778000
H	4.930190000	2.958889000	-0.833275000
C	3.138969000	4.101409000	-0.372653000
H	3.625160000	5.076312000	-0.492022000
C	1.779930000	4.036785000	-0.029411000
H	1.181340000	4.940723000	0.121361000
C	1.149751000	2.792705000	0.125310000
S	-0.616595000	2.801084000	0.578522000
O	-0.719933000	1.937309000	1.797866000
O	-1.027331000	4.221354000	0.684975000
O	-1.282096000	2.108745000	-0.647988000
C	-3.383221000	-0.533525000	-1.022127000
C	-4.277964000	-0.403319000	0.205492000
H	-4.204825000	-1.299364000	0.857565000
H	-3.972279000	0.450058000	0.839285000
C	-5.766637000	-0.264427000	-0.172032000
H	-3.766712000	-1.260669000	-1.752562000
C	-1.904029000	-1.747382000	-0.602439000
H	-3.270420000	0.426998000	-1.559111000
O	-6.178632000	-0.546890000	-1.292797000
C	-6.686901000	0.240021000	0.932411000
H	-6.485965000	-0.272508000	1.891193000
H	-6.496836000	1.317370000	1.102440000
H	-7.739460000	0.102902000	0.640556000
O	-2.086359000	-2.913448000	-0.577419000

1-cycle6-T

Zero-point correction= 0.444620 (Hartree/Particle)
Thermal correction to Energy= 0.480786
Thermal correction to Enthalpy= 0.481730

Thermal correction to Gibbs Free Energy= 0.374442
Sum of electronic and zero-point Energies= -2360.215276
Sum of electronic and thermal Energies= -2360.179110
Sum of electronic and thermal Enthalpies= -2360.178166
Sum of electronic and thermal Free Energies= -2360.285454
E solvent= -2360.03838294

Pd	-1.357163000	0.520858000	-0.186000000
P	0.821018000	-0.210836000	0.019760000
C	1.351128000	-0.821456000	1.679148000
C	1.285596000	-1.550304000	-1.155465000
C	2.035661000	1.139828000	-0.371745000
C	0.869628000	-1.425292000	-2.510797000
O	0.235314000	-0.258187000	-2.826415000
C	-0.291283000	-0.100202000	-4.147453000
H	-1.027201000	-0.892447000	-4.381391000
H	0.516644000	-0.105896000	-4.904361000
H	-0.787542000	0.881057000	-4.146819000
C	1.146296000	-2.450990000	-3.435743000
H	0.818515000	-2.362521000	-4.475066000
C	1.856909000	-3.588423000	-3.021981000
H	2.072165000	-4.377881000	-3.751364000
C	2.295912000	-3.714578000	-1.696057000
H	2.862149000	-4.596750000	-1.378955000
C	2.003707000	-2.697776000	-0.772743000
H	2.344134000	-2.791359000	0.263918000
C	0.746081000	-1.974368000	2.251982000
O	-0.208526000	-2.591532000	1.482742000
C	-0.690019000	-3.874427000	1.888642000
H	0.141651000	-4.595955000	2.002232000
H	-1.256865000	-3.817445000	2.838510000
H	-1.355921000	-4.199856000	1.075296000
C	1.136855000	-2.429383000	3.525328000
H	0.679722000	-3.323751000	3.958018000
C	2.109778000	-1.721516000	4.249376000
H	2.402265000	-2.079061000	5.243303000
C	2.694443000	-0.567359000	3.711788000
H	3.443215000	-0.006202000	4.279993000
C	2.311495000	-0.124474000	2.436380000
H	2.759501000	0.782675000	2.022607000
C	3.261572000	0.801699000	-0.985227000
H	3.476991000	-0.246344000	-1.218671000
C	4.207331000	1.789583000	-1.301848000
H	5.153071000	1.501222000	-1.774602000
C	3.937759000	3.135678000	-1.013344000
H	4.670745000	3.912069000	-1.261322000
C	2.723923000	3.489061000	-0.405161000
H	2.479542000	4.529236000	-0.167068000
C	1.777188000	2.503438000	-0.085367000
S	0.231502000	3.056712000	0.715352000
O	0.137060000	2.235307000	1.965956000
O	0.338194000	4.530384000	0.863266000
O	-0.842413000	2.657066000	-0.321526000
C	-3.211376000	-1.290745000	1.028377000
C	-4.557052000	-0.740686000	0.516609000
H	-4.937346000	-1.389593000	-0.301253000
H	-5.328447000	-0.780094000	1.309403000
C	-4.514795000	0.673770000	-0.049915000
H	-2.854902000	-0.697710000	1.888114000
C	-2.156104000	-1.273343000	-0.108511000
H	-3.354671000	-2.338178000	1.349525000
O	-3.465256000	1.234529000	-0.401125000
C	-5.831373000	1.404683000	-0.197547000
H	-6.610173000	0.742508000	-0.617625000
H	-6.182043000	1.718681000	0.804922000
H	-5.705259000	2.298813000	-0.825970000
O	-2.070037000	-2.185575000	-0.911616000

TS-2$_{Ins\text{-}E\text{-}T}$

Zero-point correction= 0.496356 (Hartree/Particle)
Thermal correction to Energy= 0.535223
Thermal correction to Enthalpy= 0.536167
Thermal correction to Gibbs Free Energy= 0.422577
Sum of electronic and zero-point Energies= -2438.720057
Sum of electronic and thermal Energies= -2438.681190
Sum of electronic and thermal Enthalpies= -2438.680246
Sum of electronic and thermal Free Energies= -2438.793835
E solvent= -2438.56256113

Pd	0.511905000	-1.440441000	0.381833000
P	-0.906587000	0.509922000	0.015602000
C	-1.818775000	1.112957000	1.508020000
C	-0.160295000	2.057378000	-0.679762000
C	-2.280857000	0.146375000	-1.183660000
C	0.498206000	1.998404000	-1.939483000
O	0.482227000	0.768370000	-2.555799000
C	0.904081000	0.690109000	-3.921347000
H	1.983978000	0.912457000	-4.029488000
H	0.321970000	1.383157000	-4.557665000
H	0.710352000	-0.347949000	-4.229121000
C	1.102093000	3.143626000	-2.489719000
H	1.594593000	3.098476000	-3.465378000
C	1.070794000	4.355433000	-1.777835000
H	1.544894000	5.243186000	-2.212009000
C	0.437353000	4.430055000	-0.530722000
H	0.407729000	5.375204000	0.021588000
C	-0.175667000	3.284878000	0.006358000
H	-0.690602000	3.346331000	0.970494000
C	-1.109431000	1.272864000	2.729324000
O	0.233119000	1.013114000	2.668163000
C	1.018125000	1.213980000	3.847514000
H	0.943338000	2.259459000	4.205035000
H	0.708609000	0.526862000	4.658879000
H	2.049546000	1.000892000	3.535269000
C	-1.780615000	1.691321000	3.894885000
H	-1.236171000	1.810288000	4.835520000
C	-3.160606000	1.946654000	3.849273000
H	-3.675057000	2.266120000	4.762923000
C	-3.874712000	1.790243000	2.654118000
H	-4.952171000	1.981884000	2.619966000
C	-3.201051000	1.372096000	1.495124000
H	-3.761309000	1.235186000	0.565364000
C	-2.781870000	1.157255000	-2.032014000
H	-2.359967000	2.166323000	-1.968020000
C	-3.811549000	0.894418000	-2.949170000
H	-4.186352000	1.701367000	-3.589366000
C	-4.357935000	-0.394584000	-3.038404000
H	-5.161577000	-0.608158000	-3.752379000
C	-3.874381000	-1.414154000	-2.205384000
H	-4.278476000	-2.430356000	-2.248132000
C	-2.847765000	-1.149195000	-1.284774000
S	-2.320011000	-2.536945000	-0.224937000
O	-2.440105000	-2.049773000	1.180362000
O	-3.118047000	-3.716594000	-0.641530000
O	-0.822391000	-2.748908000	-0.635558000
C	2.584771000	-0.457912000	0.574408000
C	3.142070000	-0.672476000	-0.842416000
H	3.271878000	-1.749727000	-1.042122000
H	2.369773000	-0.303924000	-1.543828000
C	4.455715000	0.096406000	-1.063243000
H	4.327056000	1.147049000	-0.735506000

C	5.643643000	-0.489162000	-0.299558000
O	5.512244000	-1.416204000	0.501001000
O	2.745227000	0.586412000	1.193592000
H	4.710980000	0.142293000	-2.140345000
C	7.007296000	0.129856000	-0.576051000
H	7.303558000	-0.053868000	-1.626473000
H	6.973203000	1.227089000	-0.442606000
H	7.762424000	-0.305365000	0.095807000
C	2.483565000	-1.920821000	1.534491000
C	1.683555000	-3.029193000	0.999306000
H	2.071862000	-3.604604000	0.147348000
H	1.049510000	-3.605489000	1.685352000
H	2.278600000	-1.617080000	2.569828000
H	3.576451000	-2.026708000	1.352212000

Catalyst 2

Non-Alternating pathway

1-cycle5-T

Zero-point correction= 0.540684 (Hartree/Particle)
Thermal correction to Energy= 0.585937
Thermal correction to Enthalpy= 0.586881
Thermal correction to Gibbs Free Energy= 0.459027
Sum of electronic and zero-point Energies= -2698.990221
Sum of electronic and thermal Energies= -2698.944968
Sum of electronic and thermal Enthalpies= -2698.944024
Sum of electronic and thermal Free Energies= -2699.071878
E solvent= -2698.83554202

C	-2.101547000	2.467860000	1.185885000
C	-1.262542000	3.487643000	1.684168000
H	-0.223633000	3.547932000	1.341972000
C	-1.756950000	4.424805000	2.605202000
H	-1.096216000	5.214991000	2.980207000
C	-3.090225000	4.354855000	3.040957000
H	-3.474258000	5.088861000	3.758617000
C	-3.930931000	3.341466000	2.550362000
H	-4.974258000	3.283141000	2.882027000
C	-3.441513000	2.401428000	1.630673000
C	-2.714247000	0.968727000	-1.224158000
C	-3.350888000	2.148740000	-1.678547000
H	-3.107807000	3.106257000	-1.205165000
C	-4.303052000	2.116639000	-2.704783000
H	-4.777184000	3.045930000	-3.040316000
C	-4.644103000	0.887754000	-3.289939000
H	-5.388082000	0.843039000	-4.093605000
C	-4.031036000	-0.287471000	-2.840818000
H	-4.298910000	-1.248943000	-3.291859000
C	-3.059003000	-0.278838000	-1.813179000
C	-2.477648000	-1.602754000	-1.421140000
C	-3.301744000	-2.587989000	-0.819305000
C	-2.814054000	-3.883633000	-0.547026000
H	-3.456373000	-4.645419000	-0.098005000
C	-1.485124000	-4.191852000	-0.879108000
H	-1.101465000	-5.198529000	-0.676044000
C	-0.642061000	-3.245006000	-1.472167000
H	0.386868000	-3.510056000	-1.723831000
C	-1.143101000	-1.954269000	-1.753619000
C	0.080284000	1.858438000	-0.702039000
C	0.151494000	2.955552000	-1.577613000
H	-0.763167000	3.487868000	-1.865248000
C	1.390606000	3.362708000	-2.092897000
H	1.456949000	4.218450000	-2.772589000
C	2.553680000	2.663927000	-1.717609000
H	3.525158000	2.985057000	-2.111119000
C	2.514257000	1.565962000	-0.841521000
C	1.246872000	1.112606000	-0.325246000
C	-0.439588000	-2.541917000	2.931850000
Ni	-0.539164000	-0.446095000	1.201394000
O	1.166670000	0.043729000	0.434012000
P	-1.412210000	1.158562000	0.078144000
H	-4.108776000	1.622105000	1.245472000
C	0.107470000	-3.597868000	3.857809000
C	-1.911185000	-2.340786000	2.715264000
C	-2.153362000	-0.919921000	2.160656000
H	-2.199299000	-3.093186000	1.947439000
H	-2.494119000	-2.599067000	3.626162000
H	-3.099287000	-0.875733000	1.595071000
H	-2.196499000	-0.176101000	2.981778000
O	0.347857000	-1.799740000	2.294660000
O	-4.582436000	-2.178451000	-0.518936000
C	-5.473672000	-3.147444000	0.033176000
H	-5.616462000	-4.007968000	-0.649678000
H	-5.118956000	-3.518457000	1.015020000
H	-6.432862000	-2.624507000	0.167865000
O	-0.426238000	-0.981351000	-2.389145000
C	0.951407000	-1.255601000	-2.685190000
H	1.510720000	-1.486956000	-1.761155000
H	1.043125000	-2.086211000	-3.412005000
H	1.348154000	-0.330136000	-3.125576000
C	3.750683000	0.831552000	-0.472863000
C	4.109333000	0.605678000	0.873107000
C	4.620515000	0.307908000	-1.451041000
C	5.259613000	-0.113928000	1.227397000
C	5.783784000	-0.402962000	-1.122705000
C	6.102994000	-0.617387000	0.225804000
F	4.332838000	0.459988000	-2.767042000
F	6.585902000	-0.894696000	-2.091083000
F	7.217015000	-1.299693000	0.557540000
F	5.574545000	-0.307170000	2.525222000
F	3.359209000	1.110576000	1.870588000
H	-0.356726000	-4.578950000	3.645680000
H	1.201684000	-3.668932000	3.763540000
H	-0.153473000	-3.344526000	4.903613000

TS-$1_{Coor-E-T}$

Zero-point correction= 0.592522 (Hartree/Particle)
Thermal correction to Energy= 0.640958
Thermal correction to Enthalpy= 0.641903
Thermal correction to Gibbs Free Energy= 0.508909
Sum of electronic and zero-point Energies= -2777.513222
Sum of electronic and thermal Energies= -2777.464786
Sum of electronic and thermal Enthalpies= -2777.463842
Sum of electronic and thermal Free Energies= -2777.596836
E solvent= -2777.38216119

C	-1.991621000	2.759531000	0.600450000
C	-1.210908000	3.934394000	0.558083000
H	-0.249856000	3.926568000	0.032378000
C	-1.661997000	5.113528000	1.175435000
H	-1.044291000	6.017982000	1.128689000
C	-2.896001000	5.136519000	1.843509000
H	-3.246714000	6.056909000	2.324172000
C	-3.682200000	3.971695000	1.888696000
H	-4.650412000	3.980973000	2.402980000
C	-3.235264000	2.793255000	1.273268000
C	-2.731968000	0.761504000	-1.327274000
C	-3.332865000	1.874592000	-1.966914000
H	-3.048654000	2.885947000	-1.658986000
C	-4.292968000	1.722070000	-2.974454000
H	-4.729924000	2.607154000	-3.450664000

C	-4.685828000	0.432455000	-3.359497000
H	-5.433882000	0.288139000	-4.147515000
C	-4.116791000	-0.676197000	-2.725115000
H	-4.426275000	-1.685561000	-3.016434000
C	-3.139399000	-0.547818000	-1.708184000
C	-2.642235000	-1.835759000	-1.125123000
C	-3.551350000	-2.700956000	-0.460182000
C	-3.162405000	-3.991176000	-0.040098000
H	-3.868090000	-4.658850000	0.460198000
C	-1.845530000	-4.413939000	-0.280057000
H	-1.537507000	-5.416715000	0.038674000
C	-0.919818000	-3.586336000	-0.925416000
H	0.098009000	-3.940090000	-1.102401000
C	-1.324420000	-2.305232000	-1.364806000
C	0.097623000	1.584374000	-1.120648000
C	0.121349000	2.356554000	-2.295650000
H	-0.810771000	2.775664000	-2.693226000
C	1.330249000	2.577569000	-2.971503000
H	1.355303000	3.183513000	-3.883055000
C	2.514369000	2.010240000	-2.464859000
H	3.461213000	2.179707000	-2.990743000
C	2.524066000	1.228291000	-1.297537000
C	1.287059000	0.972180000	-0.599393000
C	-0.611827000	-2.463418000	2.804959000
Ni	-0.430134000	-0.065112000	1.400505000
O	1.252508000	0.179567000	0.443009000
P	-1.369153000	1.164400000	-0.114717000
H	-3.864791000	1.896782000	1.306838000
C	-0.264585000	-3.750849000	3.514373000
C	-2.023789000	-1.951381000	2.757333000
C	-2.060612000	-0.450756000	2.402163000
H	-2.507550000	-2.547049000	1.952623000
H	-2.576904000	-2.210789000	3.686445000
H	-3.019654000	-0.204526000	1.917203000
H	-1.957379000	0.180594000	3.304784000
O	0.274776000	-1.808583000	2.212259000
O	-4.811292000	-2.185363000	-0.253023000
C	-5.787386000	-3.039803000	0.342419000
H	-5.951732000	-3.955569000	-0.258818000
H	-5.505757000	-3.329502000	1.374251000
H	-6.716597000	-2.450490000	0.372695000
O	-0.525105000	-1.460749000	-2.078431000
C	0.836601000	-1.856931000	-2.298404000
H	1.361286000	-2.001718000	-1.336973000
H	0.888050000	-2.778983000	-2.909632000
H	1.302401000	-1.023835000	-2.842639000
C	3.774544000	0.592289000	-0.812116000
C	4.229906000	0.745038000	0.513610000
C	4.561880000	-0.221904000	-1.652727000
C	5.390191000	0.115145000	0.985159000
C	5.734027000	-0.852168000	-1.211215000
C	6.149363000	-0.684179000	0.117618000
F	4.178306000	-0.446229000	-2.933518000
F	6.452984000	-1.630414000	-2.047456000
F	7.272490000	-1.284737000	0.557356000
F	5.793924000	0.289078000	2.260794000
F	3.563799000	1.541766000	1.375921000
H	-0.932686000	-4.565128000	3.176982000
H	0.784824000	-4.025868000	3.328259000
H	-0.425832000	-3.638199000	4.603804000
C	0.374131000	1.826165000	2.899620000
C	0.952460000	0.748131000	3.480408000
H	-0.571611000	2.247486000	3.258760000
H	0.885388000	2.375885000	2.103208000
H	0.483041000	0.227389000	4.323608000
H	1.921454000	0.374872000	3.137182000

1-Coor-E-T

Zero-point correction= 0.592992 (Hartree/Particle)
Thermal correction to Energy= 0.642221
Thermal correction to Enthalpy= 0.643166
Thermal correction to Gibbs Free Energy= 0.506215
Sum of electronic and zero-point Energies= -2777.518178
Sum of electronic and thermal Energies= -2777.468949
Sum of electronic and thermal Enthalpies= -2777.468004
Sum of electronic and thermal Free Energies= -2777.604955
E solvent= -2777.38775911

C	-2.198966000	-2.038768000	1.360527000
C	-1.527278000	-3.257830000	1.600892000
H	-0.433442000	-3.270117000	1.659406000
C	-2.253774000	-4.445430000	1.780198000
H	-1.718766000	-5.382329000	1.973524000
C	-3.657253000	-4.434140000	1.720585000
H	-4.222294000	-5.361716000	1.866642000
C	-4.333370000	-3.226420000	1.478992000
H	-5.428728000	-3.206519000	1.439014000
C	-3.611666000	-2.035898000	1.294774000
C	-2.168786000	0.828126000	1.856768000
C	-2.770214000	0.530522000	3.103076000
H	-2.719500000	-0.490158000	3.497223000
C	-3.450109000	1.511893000	3.834659000
H	-3.905758000	1.257154000	4.798212000
C	-3.545546000	2.814376000	3.320851000
H	-4.078858000	3.592764000	3.878405000
C	-2.955028000	3.118795000	2.088852000
H	-3.028247000	4.133464000	1.682786000
C	-2.252201000	2.148295000	1.338788000
C	-1.652459000	2.595982000	0.040696000
C	-2.496744000	2.870024000	-1.067046000
C	-1.974418000	3.409153000	-2.260842000
H	-2.620630000	3.600223000	-3.120823000
C	-0.605657000	3.707699000	-2.328060000
H	-0.198021000	4.142823000	-3.248081000
C	0.254542000	3.463907000	-1.248649000
H	1.314980000	3.714104000	-1.326812000
C	-0.271244000	2.904522000	-0.063283000
C	0.370644000	-0.702899000	1.854472000
C	0.567014000	-0.898409000	3.232868000
H	-0.296875000	-0.968829000	3.904672000
C	1.866018000	-0.997142000	3.750779000
H	2.027640000	-1.154618000	4.822001000
C	2.963735000	-0.901265000	2.875270000
H	3.980782000	-0.986369000	3.275635000
C	2.798151000	-0.711487000	1.492692000
C	1.471442000	-0.582433000	0.946042000
C	-4.267418000	-1.693201000	-2.906297000
Ni	-0.521443000	-0.340330000	-1.107433000
O	1.289794000	-0.338388000	-0.333853000
P	-1.226763000	-0.511713000	0.998343000
H	-4.149466000	-1.098325000	1.115236000
C	-4.952225000	-3.044001000	-3.117931000
C	-2.901353000	-1.738088000	-2.198373000
C	-2.336365000	-0.362124000	-1.857558000
H	-3.016901000	-2.373504000	-1.297673000
H	-2.230214000	-2.321710000	-2.863188000
H	-2.254775000	0.260606000	-2.766607000
H	-3.024270000	0.163748000	-1.173268000
O	-4.792581000	-0.647304000	-3.282515000
O	-3.821551000	2.571309000	-0.872437000
C	-4.704408000	2.666833000	-1.999616000
H	-4.721481000	3.692395000	-2.416358000

H	-4.433124000	1.931896000	-2.779141000
H	-5.702097000	2.419903000	-1.606360000
O	0.467131000	2.656219000	1.061698000
C	1.884458000	2.851497000	0.979872000
H	2.319811000	2.236928000	0.170933000
H	2.134047000	3.919286000	0.823246000
H	2.287367000	2.514691000	1.945374000
C	3.969278000	-0.603180000	0.586026000
C	4.111717000	-1.426803000	-0.549930000
C	4.992845000	0.341253000	0.806393000
C	5.199967000	-1.312916000	-1.426225000
C	6.098911000	0.467545000	-0.046744000
C	6.201929000	-0.364367000	-1.171058000
F	4.916348000	1.191268000	1.858976000
F	7.052185000	1.390424000	0.196730000
F	7.257061000	-0.254040000	-2.000377000
F	5.301310000	-2.121229000	-2.501160000
F	3.197377000	-2.384572000	-0.816506000
H	-5.211223000	-3.490701000	-2.139088000
H	-5.868122000	-2.914376000	-3.715074000
H	-4.274073000	-3.758212000	-3.621201000
C	0.371348000	-1.106916000	-2.882601000
C	0.429568000	0.275515000	-2.902890000
H	-0.375165000	-1.650152000	-3.471618000
H	1.189455000	-1.689629000	-2.445556000
H	-0.279525000	0.877534000	-3.480507000
H	1.287943000	0.799984000	-2.471860000

TS_{Isom}

Zero-point correction= 0.592595 (Hartree/Particle)
Thermal correction to Energy= 0.641006
Thermal correction to Enthalpy= 0.641950
Thermal correction to Gibbs Free Energy= 0.506914
Sum of electronic and zero-point Energies= -2777.499526
Sum of electronic and thermal Energies= -2777.451115
Sum of electronic and thermal Enthalpies= -2777.450171
Sum of electronic and thermal Free Energies= -2777.585207
E solvent= -2777.36166725

C	2.234521000	1.935275000	1.310652000
C	1.512224000	2.745600000	2.212531000
H	0.577941000	2.372604000	2.645779000
C	1.992246000	4.019221000	2.568234000
H	1.423749000	4.632596000	3.277452000
C	3.196996000	4.499014000	2.030630000
H	3.569851000	5.490981000	2.309476000
C	3.924670000	3.697126000	1.132654000
H	4.867866000	4.062495000	0.709923000
C	3.446179000	2.429695000	0.770868000
C	2.914210000	-0.877910000	1.290535000
C	3.638103000	-0.594844000	2.471305000
H	3.443877000	0.339228000	3.010283000
C	4.614963000	-1.476328000	2.953259000
H	5.161476000	-1.236889000	3.872697000
C	4.887567000	-2.659318000	2.249344000
H	5.648915000	-3.358448000	2.613827000
C	4.186922000	-2.942929000	1.070276000
H	4.403913000	-3.860524000	0.512529000
C	3.195446000	-2.070303000	0.568670000
C	2.521342000	-2.447358000	-0.715752000
C	3.245551000	-2.402126000	-1.935380000
C	2.662025000	-2.824205000	-3.148084000
H	3.217922000	-2.781504000	-4.087512000
C	1.342727000	-3.300646000	-3.136659000
H	0.885271000	-3.631908000	-4.076014000
C	0.600086000	-3.371589000	-1.951069000
H	-0.421073000	-3.758613000	-1.968315000
C	1.195786000	-2.955812000	-0.739116000
C	0.126843000	-0.064985000	1.738084000
C	0.141336000	-0.345661000	3.116633000
H	1.093070000	-0.327905000	3.661308000
C	-1.042029000	-0.668565000	3.796925000
H	-1.026117000	-0.881220000	4.870924000
C	-2.252586000	-0.709651000	3.083811000
H	-3.184262000	-0.952588000	3.608021000
C	-2.305535000	-0.435628000	1.706506000
C	-1.096461000	-0.127145000	0.989038000
Ni	0.373500000	0.644309000	-1.322824000
O	-1.147828000	0.040042000	-0.322329000
P	1.601330000	0.300286000	0.710989000
H	4.019236000	1.814556000	0.066547000
C	-0.515826000	2.403112000	-1.303754000
H	-1.426650000	2.277254000	-1.909547000
C	0.375451000	3.547999000	-1.743557000
H	0.666773000	3.479376000	-2.803951000
H	-0.776254000	2.444713000	-0.235144000
C	-0.309560000	4.934397000	-1.581823000
O	-0.388142000	5.702134000	-2.533038000
H	1.295552000	3.571558000	-1.124718000
C	-0.856102000	5.283427000	-0.205512000
H	-0.132055000	5.023135000	0.588046000
H	-1.109432000	6.354046000	-0.162604000
H	-1.770739000	4.690218000	-0.013458000
C	0.442902000	0.675688000	-3.326173000
C	1.746172000	0.837399000	-2.803395000
H	0.083629000	-0.328380000	-3.594448000
H	-0.107298000	1.509430000	-3.777263000
H	2.409228000	-0.029366000	-2.707630000
H	2.240660000	1.815178000	-2.802147000
O	4.523378000	-1.899780000	-1.838063000
C	5.321372000	-1.888821000	-3.023205000
H	5.450674000	-2.907933000	-3.437196000
H	4.889273000	-1.231216000	-3.802894000
H	6.300845000	-1.492016000	-2.715410000
O	0.581895000	-3.033749000	0.479142000
C	-0.779440000	-3.482611000	0.513344000
H	-1.424410000	-2.843672000	-0.116554000
H	-0.859019000	-4.538423000	0.188297000
H	-1.093417000	-3.389054000	1.562005000
C	-3.591168000	-0.512057000	0.963571000
C	-4.371950000	-1.684923000	0.954132000
C	-4.088951000	0.579416000	0.223962000
C	-5.582243000	-1.778411000	0.251098000
C	-5.288920000	0.510423000	-0.496779000
C	-6.041626000	-0.673930000	-0.480776000
F	-3.950103000	-2.786894000	1.621081000
F	-6.296242000	-2.922701000	0.262064000
F	-7.200286000	-0.748253000	-1.162222000
F	-5.733682000	1.577314000	-1.191241000
F	-3.420891000	1.752308000	0.212105000

1-Coor-E-C

Zero-point correction= 0.593721 (Hartree/Particle)
Thermal correction to Energy= 0.642334
Thermal correction to Enthalpy= 0.643278
Thermal correction to Gibbs Free Energy= 0.509336
Sum of electronic and zero-point Energies= -2777.516172
Sum of electronic and thermal Energies= -2777.467560
Sum of electronic and thermal Enthalpies= -2777.466615
Sum of electronic and thermal Free Energies= -2777.600557

E solvent= -2777.37812055

C	-2.441961000	1.977758000	-1.712804000
C	-1.668427000	2.570277000	-2.734333000
H	-0.749383000	2.079499000	-3.071927000
C	-2.074283000	3.779083000	-3.323667000
H	-1.463948000	4.223488000	-4.118422000
C	-3.253413000	4.414315000	-2.903313000
H	-3.566986000	5.357327000	-3.365054000
C	-4.028430000	3.835288000	-1.884001000
H	-4.950879000	4.323231000	-1.548366000
C	-3.624556000	2.630774000	-1.288352000
C	-3.282879000	-0.689095000	-0.896352000
C	-4.247174000	-0.544331000	-1.919897000
H	-4.146908000	0.273649000	-2.640864000
C	-5.338172000	-1.419068000	-2.018106000
H	-6.071474000	-1.287802000	-2.822101000
C	-5.484002000	-2.454255000	-1.082688000
H	-6.335635000	-3.141485000	-1.143887000
C	-4.530946000	-2.610714000	-0.067581000
H	-4.635030000	-3.422556000	0.660789000
C	-3.419135000	-1.747910000	0.043442000
C	-2.431031000	-2.023781000	1.136713000
C	-2.733947000	-1.709945000	2.485780000
C	-1.854033000	-2.059698000	3.530327000
H	-2.079897000	-1.806167000	4.568786000
C	-0.671162000	-2.746665000	3.218802000
H	0.017336000	-3.018580000	4.027109000
C	-0.355010000	-3.104562000	1.901791000
H	0.566532000	-3.651155000	1.689196000
C	-1.245543000	-2.755938000	0.864085000
C	-0.511383000	-0.225657000	-1.817118000
C	-0.660097000	-0.788217000	-3.096424000
H	-1.654228000	-0.825714000	-3.558661000
C	0.451224000	-1.310405000	-3.773926000
H	0.343215000	-1.745158000	-4.772996000
C	1.713023000	-1.256723000	-3.155870000
H	2.590837000	-1.648968000	-3.683040000
C	1.892688000	-0.700568000	-1.876542000
C	0.756982000	-0.187612000	-1.154722000
Ni	-0.549580000	1.026115000	1.091751000
O	0.904377000	0.272677000	0.076249000
P	-1.846737000	0.482715000	-0.800404000
H	-4.243051000	2.187893000	-0.498170000
C	0.730747000	1.180547000	2.579609000
H	0.262474000	1.681659000	3.443679000
C	2.076717000	1.790283000	2.227222000
H	2.323930000	1.575072000	1.168902000
H	0.801211000	0.089510000	2.752871000
C	2.214380000	3.294116000	2.432928000
O	1.273868000	4.026266000	2.745315000
H	2.900032000	1.306758000	2.802209000
C	3.619503000	3.862033000	2.238764000
H	4.132191000	3.394173000	1.380171000
H	3.570536000	4.954527000	2.109423000
H	4.229800000	3.643127000	3.136849000
C	-1.474667000	2.743863000	1.695241000
C	-2.111879000	1.644340000	2.283027000
H	-0.720830000	3.333136000	2.231520000
H	-1.880178000	3.189707000	0.777547000
H	-1.870178000	1.344026000	3.309702000
H	-3.045385000	1.233137000	1.884411000
O	-3.933304000	-1.060759000	2.678489000
C	-4.340200000	-0.812347000	4.025871000
H	-4.409757000	-1.751694000	4.608148000
H	-3.651657000	-0.114222000	4.540803000
H	-5.336702000	-0.350554000	3.953407000
O	-1.076015000	-3.110626000	-0.445575000
C	0.067966000	-3.902275000	-0.784411000
H	1.013394000	-3.365155000	-0.589850000
H	0.063342000	-4.865595000	-0.237704000
H	-0.018643000	-4.087274000	-1.865058000
C	3.246848000	-0.662299000	-1.262662000
C	4.000926000	-1.833965000	-1.053408000
C	3.860436000	0.547971000	-0.884295000
C	5.285819000	-1.816401000	-0.490808000
C	5.138232000	0.595462000	-0.311244000
C	5.858812000	-0.592172000	-0.116721000
F	3.477966000	-3.043228000	-1.372105000
F	5.966896000	-2.964054000	-0.297944000
F	7.089953000	-0.557332000	0.426407000
F	5.687101000	1.779023000	0.041850000
F	3.218301000	1.722336000	-1.070913000

TS-1$_{Ins\text{-}E\text{-}C}$

Zero-point correction= 0.594420 (Hartree/Particle)
Thermal correction to Energy= 0.642283
Thermal correction to Enthalpy= 0.643227
Thermal correction to Gibbs Free Energy= 0.510198
Sum of electronic and zero-point Energies= -2777.500739
Sum of electronic and thermal Energies= -2777.452876
Sum of electronic and thermal Enthalpies= -2777.451932
Sum of electronic and thermal Free Energies= -2777.584962
E solvent= -2777.37077766

C	-2.342705000	1.991104000	-1.735883000
C	-1.533632000	2.752360000	-2.606720000
H	-0.542066000	2.381300000	-2.888062000
C	-1.997702000	3.974119000	-3.120975000
H	-1.359900000	4.551558000	-3.800245000
C	-3.271035000	4.452254000	-2.772653000
H	-3.631073000	5.405662000	-3.175678000
C	-4.082921000	3.700715000	-1.906424000
H	-5.080239000	4.064185000	-1.632721000
C	-3.623061000	2.480470000	-1.388345000
C	-3.066249000	-0.761208000	-1.126384000
C	-3.878627000	-0.686213000	-2.282436000
H	-3.713431000	0.121464000	-3.003718000
C	-4.904406000	-1.611917000	-2.513073000
H	-5.518237000	-1.533044000	-3.417552000
C	-5.138432000	-2.631299000	-1.577777000
H	-5.938980000	-3.361731000	-1.741759000
C	-4.342739000	-2.713498000	-0.428806000
H	-4.522148000	-3.507373000	0.304492000
C	-3.295859000	-1.796910000	-0.180382000
C	-2.505559000	-1.995146000	1.078318000
C	-3.095836000	-1.721765000	2.338246000
C	-2.421350000	-2.014930000	3.541216000
H	-2.876598000	-1.801846000	4.511427000
C	-1.146904000	-2.598064000	3.477957000
H	-0.619249000	-2.833068000	4.409388000
C	-0.536751000	-2.893882000	2.252467000
H	0.451691000	-3.358275000	2.232460000
C	-1.221231000	-2.599217000	1.052769000
C	-0.311589000	-0.118028000	-1.997918000
C	-0.381076000	-0.498338000	-3.349450000
H	-1.342836000	-0.470865000	-3.876148000
C	0.773657000	-0.923886000	-4.022854000
H	0.726481000	-1.222247000	-5.075154000
C	2.001505000	-0.943623000	-3.334742000
H	2.912631000	-1.250508000	-3.862147000
C	2.100568000	-0.563786000	-1.986026000

C	0.915963000	-0.178197000	-1.262832000
Ni	-0.641620000	0.762412000	0.944256000
O	0.965680000	0.083608000	0.026415000
P	-1.682208000	0.456975000	-0.943416000
H	-4.269899000	1.897536000	-0.722738000
C	0.663424000	0.899497000	2.639382000
H	0.589765000	0.920952000	3.735014000
C	1.753111000	1.860630000	2.172676000
H	1.830957000	1.894202000	1.071399000
H	0.876520000	-0.141411000	2.339152000
C	1.730768000	3.281209000	2.720454000
O	0.949598000	3.667327000	3.591577000
H	2.741402000	1.449999000	2.488584000
C	2.762149000	4.227406000	2.105333000
H	2.295244000	4.735746000	1.239005000
H	3.051697000	5.001335000	2.834186000
H	3.650572000	3.692211000	1.728174000
C	-2.201833000	1.642249000	1.710099000
C	-1.300330000	1.559721000	2.829829000
H	-2.333708000	2.612282000	1.209260000
H	-3.085259000	0.989496000	1.697819000
H	-0.887440000	2.486540000	3.244762000
H	-1.533488000	0.791495000	3.578050000
O	-4.346826000	-1.145911000	2.283712000
C	-5.006194000	-0.880093000	3.524057000
H	-5.158050000	-1.807159000	4.110440000
H	-4.444709000	-0.147692000	4.136101000
H	-5.983998000	-0.453904000	3.252856000
O	-0.742462000	-2.893481000	-0.193046000
C	0.584541000	-3.427303000	-0.285643000
H	1.318701000	-2.736730000	0.166931000
H	0.649637000	-4.422217000	0.196883000
H	0.790177000	-3.521287000	-1.361063000
C	3.406553000	-0.560244000	-1.277882000
C	4.198026000	-1.717574000	-1.145796000
C	3.913201000	0.615002000	-0.688189000
C	5.420335000	-1.717860000	-0.455898000
C	5.117312000	0.639567000	0.026576000
C	5.880580000	-0.532968000	0.137469000
F	3.774398000	-2.891394000	-1.671546000
F	6.145469000	-2.849463000	-0.344165000
F	7.047131000	-0.520854000	0.809676000
F	5.556850000	1.785810000	0.592234000
F	3.237525000	1.779984000	-0.813440000

1-β-T

Zero-point correction= 0.594810 (Hartree/Particle)
Thermal correction to Energy= 0.643292
Thermal correction to Enthalpy= 0.644236
Thermal correction to Gibbs Free Energy= 0.506894
Sum of electronic and zero-point Energies= -2777.534372
Sum of electronic and thermal Energies= -2777.485890
Sum of electronic and thermal Enthalpies= -2777.484946
Sum of electronic and thermal Free Energies= -2777.622287
E solvent= -2777.40531598

C	2.516045000	0.176853000	2.535092000
C	1.818253000	0.109810000	3.759747000
H	0.849166000	-0.398624000	3.804379000
C	2.364793000	0.686413000	4.917836000
H	1.814951000	0.623596000	5.863979000
C	3.607469000	1.337645000	4.866570000
H	4.031312000	1.787166000	5.771687000
C	4.305502000	1.412235000	3.648768000
H	5.276434000	1.918857000	3.601322000
C	3.764473000	0.838752000	2.488525000
C	3.107709000	-1.443684000	0.186458000
C	3.974995000	-2.181048000	1.026211000
H	3.865169000	-2.105662000	2.113574000
C	4.987003000	-2.989724000	0.493787000
H	5.645421000	-3.553952000	1.163964000
C	5.150923000	-3.064362000	-0.897869000
H	5.939686000	-3.690663000	-1.330161000
C	4.303005000	-2.331829000	-1.737634000
H	4.431444000	-2.383696000	-2.824270000
C	3.269093000	-1.516709000	-1.224015000
C	2.427668000	-0.769074000	-2.213974000
C	2.983877000	0.316970000	-2.936658000
C	2.249600000	0.981226000	-3.940292000
H	2.679080000	1.819435000	-4.494455000
C	0.946298000	0.546790000	-4.225090000
H	0.370973000	1.055844000	-5.007145000
C	0.366515000	-0.527933000	-3.539724000
H	-0.646468000	-0.851249000	-3.788359000
C	1.113005000	-1.194231000	-2.541472000
C	0.417227000	-1.609505000	1.461072000
C	0.543324000	-2.774607000	2.237619000
H	1.525999000	-3.070135000	2.625561000
C	-0.583527000	-3.562654000	2.513729000
H	-0.494372000	-4.467978000	3.122849000
C	-1.836337000	-3.168417000	2.009393000
H	-2.722027000	-3.774321000	2.232831000
C	-1.994510000	-2.008658000	1.230661000
C	-0.844306000	-1.204244000	0.908021000
Ni	0.603386000	0.935107000	-0.106759000
O	-0.952908000	-0.157028000	0.114458000
P	1.756668000	-0.464423000	0.981644000
H	4.319044000	0.896856000	1.544752000
C	-0.030853000	4.212258000	-0.155042000
H	-0.057912000	3.973818000	0.924189000
C	-1.438766000	4.600339000	-0.625395000
H	-2.153530000	3.765324000	-0.471861000
H	0.645823000	5.078996000	-0.261512000
C	-1.994211000	5.855714000	0.057843000
O	-1.292863000	6.570478000	0.768624000
H	-1.450019000	4.789255000	-1.720969000
C	-3.468486000	6.164373000	-0.189022000
C	1.805002000	2.391953000	-0.331984000
C	0.561444000	3.014214000	-0.923542000
H	2.139654000	2.817840000	0.628511000
H	2.638811000	2.208557000	-1.024915000
H	0.679151000	3.222205000	-2.004238000
H	-0.306275000	2.226292000	-0.962034000
O	4.261009000	0.674279000	-2.563244000
C	4.896220000	1.720169000	-3.300650000
H	4.976294000	1.469798000	-4.376557000
H	4.359828000	2.682914000	-3.189138000
H	5.904879000	1.814732000	-2.870286000
O	0.666960000	-2.290721000	-1.859171000
C	-0.678277000	-2.722644000	-2.105995000
H	-1.394905000	-1.909726000	-1.891544000
H	-0.797781000	-3.075003000	-3.149329000
H	-0.854374000	-3.554954000	-1.410277000
C	-3.325319000	-1.618143000	0.700221000
C	-4.112500000	-2.509524000	-0.057629000
C	-3.863769000	-0.331278000	0.911775000
C	-5.363968000	-2.153389000	-0.581142000
C	-5.105294000	0.054385000	0.386687000
C	-5.861839000	-0.861183000	-0.359940000
F	-3.655133000	-3.755933000	-0.329472000
F	-6.081170000	-3.035419000	-1.308138000
F	-7.060672000	-0.502445000	-0.858816000

F	-5.587560000	1.295326000	0.610876000
F	-3.199026000	0.570395000	1.660888000
H	-3.713038000	7.166240000	0.196008000
H	-3.720702000	6.099032000	-1.263693000
H	-4.096950000	5.415609000	0.330659000

TS-$1_{Ins-E-T}$

Zero-point correction= 0.593481 (Hartree/Particle)
Thermal correction to Energy= 0.641773
Thermal correction to Enthalpy= 0.642717
Thermal correction to Gibbs Free Energy= 0.507715
Sum of electronic and zero-point Energies= -2777.483437
Sum of electronic and thermal Energies= -2777.435145
Sum of electronic and thermal Enthalpies= -2777.434201
Sum of electronic and thermal Free Energies= -2777.569203
E solvent= -2777.35359147

C	2.411825000	1.124385000	2.054691000
C	1.778803000	2.070167000	2.891832000
H	0.697630000	2.006094000	3.057716000
C	2.525858000	3.081136000	3.517178000
H	2.020327000	3.800781000	4.171813000
C	3.913129000	3.169462000	3.311990000
H	4.494158000	3.959555000	3.801093000
C	4.552827000	2.232739000	2.482161000
H	5.636213000	2.287892000	2.323199000
C	3.810312000	1.217782000	1.857572000
C	2.392054000	-1.640935000	1.213855000
C	3.057274000	-1.925076000	2.429816000
H	3.024844000	-1.193402000	3.244310000
C	3.776288000	-3.113990000	2.606473000
H	4.279890000	-3.311741000	3.559681000
C	3.848182000	-4.041541000	1.555705000
H	4.409565000	-4.975029000	1.677130000
C	3.198830000	-3.769097000	0.345685000
H	3.253013000	-4.488561000	-0.478789000
C	2.461258000	-2.579231000	0.150075000
C	1.798260000	-2.403456000	-1.183814000
C	2.574554000	-2.105248000	-2.332097000
C	1.998968000	-2.092318000	-3.620032000
H	2.599013000	-1.869194000	-4.505764000
C	0.632900000	-2.385758000	-3.754104000
H	0.181167000	-2.385297000	-4.752734000
C	-0.165806000	-2.680852000	-2.641811000
H	-1.225028000	-2.911659000	-2.776496000
C	0.419684000	-2.693741000	-1.356484000
C	-0.143866000	-0.319923000	1.971682000
C	-0.287210000	-0.760391000	3.300253000
H	0.603524000	-0.979824000	3.901409000
C	-1.562186000	-0.927051000	3.857488000
H	-1.678549000	-1.264216000	4.892577000
C	-2.695183000	-0.646946000	3.071493000
H	-3.695515000	-0.766149000	3.503639000
C	-2.586717000	-0.201952000	1.743225000
C	-1.283402000	-0.050165000	1.148563000
Ni	0.516908000	0.716133000	-0.896167000
O	-1.180875000	0.302127000	-0.118823000
P	1.411937000	-0.073625000	1.062286000
H	4.323496000	0.484443000	1.224666000
C	2.305655000	1.692744000	-1.625223000
H	2.856564000	0.915092000	-1.077229000
C	2.144548000	3.014606000	-0.872738000
H	2.850087000	3.066324000	-0.018478000
H	2.880166000	1.871247000	-2.546051000
C	2.372720000	4.261250000	-1.735110000
O	2.729100000	4.194703000	-2.911234000
H	1.144327000	3.110830000	-0.395462000
C	2.115482000	5.601359000	-1.053910000
H	2.548295000	5.629190000	-0.037658000
H	2.522869000	6.420991000	-1.665577000
H	1.024075000	5.750989000	-0.940713000
C	0.817531000	1.408626000	-2.994837000
C	-0.496450000	1.126407000	-2.513603000
H	1.030209000	2.428286000	-3.337454000
H	1.317714000	0.628503000	-3.582068000
H	-1.194179000	1.944505000	-2.295135000
H	-0.949287000	0.147191000	-2.710184000
O	3.900523000	-1.830054000	-2.083208000
C	4.746723000	-1.588686000	-3.208822000
H	4.762116000	-2.454264000	-3.899629000
H	4.439080000	-0.682774000	-3.767151000
H	5.754335000	-1.433997000	-2.793621000
O	-0.255923000	-3.010628000	-0.211709000
C	-1.678544000	-3.164000000	-0.302851000
H	-2.141875000	-2.257182000	-0.731863000
H	-1.948869000	-4.053543000	-0.905023000
H	-2.029366000	-3.295325000	0.730393000
C	-3.798393000	0.078616000	0.931516000
C	-4.813273000	-0.885345000	0.761377000
C	-3.994511000	1.316443000	0.284068000
C	-5.961617000	-0.644189000	-0.006818000
C	-5.126625000	1.578218000	-0.500543000
C	-6.117455000	0.595274000	-0.644096000
F	-4.688946000	-2.109679000	1.329613000
F	-6.905543000	-1.597806000	-0.149641000
F	-7.213526000	0.841388000	-1.387145000
F	-5.279562000	2.775587000	-1.103157000
F	-3.094603000	2.310263000	0.426865000

Alternating pathway

TS-$1_{Coor-CO-T}$

Zero-point correction= 0.548730 (Hartree/Particle)
Thermal correction to Energy= 0.595992
Thermal correction to Enthalpy= 0.596936
Thermal correction to Gibbs Free Energy= 0.464800
Sum of electronic and zero-point Energies= -2812.298957
Sum of electronic and thermal Energies= -2812.251695
Sum of electronic and thermal Enthalpies= -2812.250751
Sum of electronic and thermal Free Energies= -2812.382888
E solvent= -2812.130573587

C	-1.524927000	2.766876000	0.842195000
C	-0.452980000	3.645129000	1.112823000
H	0.532078000	3.449468000	0.675086000
C	-0.645720000	4.768110000	1.932760000
H	0.195032000	5.442927000	2.130524000
C	-1.907111000	5.029896000	2.492452000
H	-2.055073000	5.908140000	3.131024000
C	-2.980029000	4.163596000	2.224191000
H	-3.970192000	4.364863000	2.649370000
C	-2.792083000	3.038022000	1.406968000
C	-2.682156000	1.147188000	-1.269741000
C	-3.113698000	2.398340000	-1.777402000
H	-2.655698000	3.317065000	-1.396528000
C	-4.122419000	2.494106000	-2.743836000
H	-4.427823000	3.477409000	-3.118763000
C	-4.729457000	1.323537000	-3.221006000
H	-5.516218000	1.374074000	-3.982355000
C	-4.328558000	0.083371000	-2.714416000

H	-4.808755000	-0.830928000	-3.077612000
C	-3.312725000	-0.041486000	-1.735342000
C	-3.014384000	-1.432781000	-1.268691000
C	-4.051555000	-2.220778000	-0.695245000
C	-3.842953000	-3.575730000	-0.364033000
H	-4.644475000	-4.178691000	0.069000000
C	-2.581637000	-4.145715000	-0.593155000
H	-2.412633000	-5.197295000	-0.334702000
C	-1.533837000	-3.401097000	-1.146830000
H	-0.560527000	-3.866749000	-1.312270000
C	-1.757496000	-2.052540000	-1.502125000
C	0.232868000	1.522394000	-1.158211000
C	0.259556000	2.258739000	-2.356124000
H	-0.661162000	2.714325000	-2.737721000
C	1.457480000	2.398227000	-3.070092000
H	1.485731000	2.978963000	-3.997835000
C	2.629257000	1.794052000	-2.577770000
H	3.569166000	1.907324000	-3.130044000
C	2.634013000	1.048019000	-1.387381000
C	1.403581000	0.866632000	-0.661121000
C	-1.250021000	-2.827147000	2.737412000
Ni	-0.196646000	0.007263000	1.527234000
O	1.368064000	0.073768000	0.390429000
P	-1.227159000	1.204107000	-0.109402000
H	-3.642475000	2.380558000	1.190674000
C	-1.792427000	-4.155154000	3.249964000
C	-2.274932000	-1.731173000	2.458091000
C	-1.793203000	-0.293030000	2.671368000
H	-2.578103000	-1.882548000	1.401492000
H	-3.191414000	-1.926850000	3.057060000
H	-2.564448000	0.430062000	2.361612000
H	-1.581234000	-0.105935000	3.738369000
O	-0.044952000	-2.666775000	2.532900000
O	-5.242346000	-1.566184000	-0.480953000
C	-6.321582000	-2.316179000	0.077788000
H	-6.610928000	-3.164984000	-0.572356000
H	-6.073345000	-2.699399000	1.087076000
H	-7.162863000	-1.610485000	0.153042000
O	-0.825415000	-1.266007000	-2.115928000
C	0.474570000	-1.829147000	-2.354828000
H	0.964756000	-2.101669000	-1.402948000
H	0.407106000	-2.710086000	-3.021433000
H	1.052816000	-1.034189000	-2.845798000
C	3.871558000	0.387917000	-0.899760000
C	4.355627000	0.587480000	0.409549000
C	4.617192000	-0.481415000	-1.721365000
C	5.510323000	-0.050772000	0.883893000
C	5.781398000	-1.123251000	-1.275700000
C	6.228487000	-0.907636000	0.035965000
F	4.201767000	-0.743857000	-2.984363000
F	6.463286000	-1.954227000	-2.091309000
F	7.344951000	-1.517181000	0.478113000
F	5.946758000	0.167269000	2.140555000
F	3.721229000	1.433456000	1.245415000
H	-2.624461000	-4.501478000	2.609042000
H	-0.993986000	-4.912376000	3.277322000
H	-2.206376000	-4.021000000	4.268031000
C	0.770126000	0.011255000	3.010682000
O	1.372922000	0.097482000	3.998905000

1-Coor-CO-T

Zero-point correction= 0.548921 (Hartree/Particle)
Thermal correction to Energy= 0.597138
Thermal correction to Enthalpy= 0.598082
Thermal correction to Gibbs Free Energy= 0.463213
Sum of electronic and zero-point Energies= -2812.308593
Sum of electronic and thermal Energies= -2812.260376
Sum of electronic and thermal Enthalpies= -2812.259432
Sum of electronic and thermal Free Energies= -2812.394301
E solvent= -2812.14776567

C	2.226088000	1.736011000	1.610995000
C	1.606624000	2.955037000	1.966245000
H	0.513894000	3.011331000	2.023221000
C	2.385372000	4.085559000	2.258845000
H	1.892984000	5.022856000	2.542186000
C	3.787549000	4.016102000	2.195780000
H	4.393168000	4.898901000	2.430104000
C	4.409959000	2.808490000	1.837611000
H	5.503213000	2.743982000	1.792093000
C	3.636554000	1.673670000	1.543945000
C	2.098712000	-1.171262000	1.738030000
C	2.627927000	-1.095341000	3.048501000
H	2.553032000	-0.156834000	3.608620000
C	3.268209000	-2.192916000	3.635933000
H	3.666987000	-2.113082000	4.653424000
C	3.397336000	-3.386812000	2.909012000
H	3.897802000	-4.254364000	3.354016000
C	2.888404000	-3.467468000	1.607427000
H	2.996328000	-4.394097000	1.033610000
C	2.229011000	-2.376253000	0.997096000
C	1.740147000	-2.557044000	-0.406518000
C	2.674279000	-2.602679000	-1.473575000
C	2.249407000	-2.835682000	-2.797790000
H	2.964984000	-2.852959000	-3.623127000
C	0.884156000	-3.035899000	-3.047166000
H	0.550032000	-3.220176000	-4.074730000
C	-0.064930000	-3.005923000	-2.016247000
H	-1.120817000	-3.173071000	-2.239813000
C	0.366396000	-2.771461000	-0.690938000
C	-0.395878000	0.432009000	1.968026000
C	-0.590693000	0.472930000	3.361200000
H	0.272550000	0.459289000	4.036973000
C	-1.887884000	0.526327000	3.886940000
H	-2.048511000	0.565200000	4.969069000
C	-2.985804000	0.536597000	3.006247000
H	-4.002938000	0.581226000	3.412469000
C	-2.822066000	0.504104000	1.611762000
C	-1.496817000	0.433550000	1.053835000
Ni	0.463748000	0.433183000	-1.016040000
O	-1.330178000	0.349272000	-0.251856000
P	1.193242000	0.301238000	1.092106000
H	4.132279000	0.733768000	1.277589000
C	2.318488000	0.640254000	-1.718199000
H	3.025093000	0.183206000	-1.006630000
C	2.663762000	2.105565000	-1.955937000
H	2.519628000	2.717524000	-1.042939000
H	2.377820000	0.051060000	-2.647999000
C	4.109334000	2.319507000	-2.442635000
O	4.838332000	1.390337000	-2.782657000
H	2.001468000	2.569018000	-2.717709000
C	4.596426000	3.766569000	-2.470184000
H	4.694018000	4.140098000	-1.432847000
H	5.574171000	3.828432000	-2.972606000
H	3.867486000	4.424882000	-2.978015000
O	3.980575000	-2.387864000	-1.110361000
C	4.947519000	-2.224381000	-2.157696000
H	5.010979000	-3.125394000	-2.797950000
H	4.724127000	-1.329711000	-2.767591000
H	5.909486000	-2.079574000	-1.642970000
O	-0.463593000	-2.764694000	0.397111000
C	-1.872012000	-2.887985000	0.152199000

H	-2.221833000	-2.083710000	-0.519379000
H	-2.116903000	-3.880999000	-0.272661000
H	-2.355026000	-2.776286000	1.133602000
C	-3.997003000	0.501171000	0.702436000
C	-5.007268000	-0.475839000	0.809377000
C	-4.154628000	1.462765000	-0.316778000
C	-6.118342000	-0.505827000	-0.045771000
C	-5.249907000	1.448277000	-1.192288000
C	-6.238406000	0.462018000	-1.053729000
F	-4.911142000	-1.450387000	1.745975000
F	-7.060047000	-1.463033000	0.084079000
F	-7.298975000	0.446042000	-1.883217000
F	-5.370126000	2.385397000	-2.154294000
F	-3.251691000	2.452344000	-0.462137000
C	-0.121442000	0.688173000	-2.674824000
O	-0.485476000	0.867579000	-3.762628000

TS-1$_{Ins\text{-}CO\text{-}T}$

Zero-point correction= 0.548033 (Hartree/Particle)
Thermal correction to Energy= 0.595867
Thermal correction to Enthalpy= 0.596811
Thermal correction to Gibbs Free Energy= 0.461133
Sum of electronic and zero-point Energies= -2812.292507
Sum of electronic and thermal Energies= -2812.244673
Sum of electronic and thermal Enthalpies= -2812.243729
Sum of electronic and thermal Free Energies= -2812.379407
E solvent= -2812.12447708

C	2.083248000	0.567228000	2.366769000
C	1.376645000	1.340587000	3.314222000
H	0.284833000	1.265814000	3.368637000
C	2.067325000	2.192806000	4.190714000
H	1.506506000	2.781624000	4.925799000
C	3.467947000	2.286182000	4.133978000
H	4.004346000	2.947879000	4.823474000
C	4.178161000	1.521523000	3.192237000
H	5.272241000	1.579040000	3.148395000
C	3.492638000	0.670367000	2.310323000
C	2.166885000	-1.969840000	0.940523000
C	2.686553000	-2.593764000	2.097804000
H	2.534966000	-2.126186000	3.077246000
C	3.415863000	-3.786249000	2.008895000
H	3.808627000	-4.256890000	2.917297000
C	3.642046000	-4.366427000	0.750331000
H	4.212282000	-5.298626000	0.666215000
C	3.145005000	-3.747419000	-0.403372000
H	3.331454000	-4.191399000	-1.387161000
C	2.402229000	-2.547679000	-0.336017000
C	1.932629000	-1.932626000	-1.618969000
C	2.870275000	-1.335536000	-2.502286000
C	2.463945000	-0.813457000	-3.748838000
H	3.184892000	-0.350256000	-4.426303000
C	1.110171000	-0.885599000	-4.106952000
H	0.789770000	-0.479476000	-5.073242000
C	0.155047000	-1.459867000	-3.257217000
H	-0.892539000	-1.503644000	-3.562465000
C	0.570370000	-1.995471000	-2.016830000
C	-0.445094000	-0.849790000	1.817368000
C	-0.669469000	-1.642634000	2.958957000
H	0.182803000	-2.047845000	3.517038000
C	-1.974215000	-1.929182000	3.379688000
H	-2.150496000	-2.544216000	4.268078000
C	-3.058860000	-1.408057000	2.651348000
H	-4.083158000	-1.619880000	2.978989000
C	-2.871364000	-0.605546000	1.513915000
C	-1.538044000	-0.323753000	1.054441000
Ni	0.350551000	0.881887000	-0.619595000
O	-1.375442000	0.379317000	-0.057814000
P	1.174442000	-0.423037000	1.099665000
H	4.056588000	0.071494000	1.585562000
C	1.915307000	2.005875000	-1.551600000
H	2.392548000	1.039070000	-1.304711000
C	2.497274000	3.146153000	-0.721069000
H	2.365000000	2.955390000	0.361973000
H	2.072700000	2.129791000	-2.633524000
C	3.989305000	3.386450000	-0.992627000
O	4.605040000	2.776224000	-1.863965000
H	1.960765000	4.096594000	-0.921824000
C	4.667994000	4.422060000	-0.103621000
H	4.693466000	4.052207000	0.939340000
H	5.696069000	4.603688000	-0.452414000
H	4.100331000	5.370768000	-0.091204000
O	4.161984000	-1.290320000	-2.036976000
C	5.132820000	-0.604199000	-2.839811000
H	5.243707000	-1.077050000	-3.834994000
H	4.875304000	0.465387000	-2.950543000
H	6.081105000	-0.695557000	-2.288575000
O	-0.265481000	-2.624174000	-1.135916000
C	-1.664088000	-2.658607000	-1.457744000
H	-2.061918000	-1.635377000	-1.581010000
H	-1.846877000	-3.256997000	-2.371386000
H	-2.152753000	-3.137193000	-0.597159000
C	-4.032580000	-0.069033000	0.757457000
C	-5.028961000	-0.918238000	0.236244000
C	-4.189547000	1.311639000	0.517476000
C	-6.126550000	-0.432002000	-0.488852000
C	-5.271354000	1.822371000	-0.213839000
C	-6.245986000	0.946775000	-0.716451000
F	-4.933813000	-2.259168000	0.406110000
F	-7.056687000	-1.278224000	-0.978026000
F	-7.294094000	1.428554000	-1.411106000
F	-5.392999000	3.148605000	-0.421569000
F	-3.299492000	2.191533000	1.014996000
C	0.117668000	2.252308000	-1.653249000
O	-0.337135000	3.190050000	-2.220523000

1-cycle6-C

Zero-point correction= 0.550486 (Hartree/Particle)
Thermal correction to Energy= 0.597943
Thermal correction to Enthalpy= 0.598888
Thermal correction to Gibbs Free Energy= 0.464638
Sum of electronic and zero-point Energies= -2812.308190
Sum of electronic and thermal Energies= -2812.260732
Sum of electronic and thermal Enthalpies= -2812.259788
Sum of electronic and thermal Free Energies= -2812.394037
E solvent= -2812.13721421

C	-1.990291000	2.173607000	1.633907000
C	-1.100297000	2.941865000	2.415896000
H	-0.052913000	3.036773000	2.108717000
C	-1.551932000	3.591130000	3.576238000
H	-0.849516000	4.189017000	4.168785000
C	-2.894542000	3.483170000	3.973474000
H	-3.245367000	3.994199000	4.877393000
C	-3.786307000	2.717182000	3.203163000
H	-4.837923000	2.631861000	3.502415000
C	-3.338553000	2.062060000	2.046028000
C	-2.659732000	1.472677000	-1.098219000
C	-3.243957000	2.755168000	-1.220113000
H	-2.963669000	3.540884000	-0.509879000

C	-4.192752000	3.032765000	-2.212893000
H	-4.627309000	4.036118000	-2.289128000
C	-4.580608000	2.017877000	-3.101453000
H	-5.320817000	2.219281000	-3.884402000
C	-4.022138000	0.739681000	-2.981211000
H	-4.329019000	-0.057610000	-3.666886000
C	-3.061134000	0.440238000	-1.988747000
C	-2.541444000	-0.964623000	-1.934525000
C	-3.402464000	-2.028555000	-1.562966000
C	-2.969324000	-3.370365000	-1.607116000
H	-3.638324000	-4.189313000	-1.331628000
C	-1.661316000	-3.647436000	-2.033521000
H	-1.324249000	-4.689462000	-2.087861000
C	-0.779824000	-2.623053000	-2.402437000
H	0.231404000	-2.865585000	-2.736194000
C	-1.224481000	-1.282309000	-2.359361000
C	0.155283000	1.993636000	-0.357341000
C	0.257820000	3.267789000	-0.946456000
H	-0.647587000	3.864948000	-1.107141000
C	1.502787000	3.771739000	-1.345762000
H	1.581010000	4.762337000	-1.805785000
C	2.656516000	2.994410000	-1.135180000
H	3.637212000	3.387424000	-1.427820000
C	2.591481000	1.723074000	-0.542644000
C	1.314792000	1.176298000	-0.167388000
C	0.726509000	-2.306061000	1.516487000
Ni	-0.309220000	-0.822029000	0.967158000
O	1.271568000	-0.059349000	0.311064000
P	-1.375278000	1.175432000	0.204140000
H	-4.044235000	1.473843000	1.447730000
C	-0.081845000	-3.625748000	1.469008000
C	-1.149473000	-3.645731000	2.576544000
H	-0.663595000	-3.602108000	3.575933000
H	0.618060000	-4.469911000	1.603723000
C	-2.123043000	-2.490106000	2.494161000
O	-1.871983000	-1.454993000	1.831239000
O	1.885043000	-2.284778000	1.880649000
H	-1.717881000	-4.595914000	2.567251000
C	-3.426612000	-2.571790000	3.248716000
H	-3.242310000	-2.808396000	4.314366000
H	-3.974019000	-1.620567000	3.170793000
H	-4.052378000	-3.392718000	2.847807000
O	-4.664454000	-1.649404000	-1.161988000
C	-5.608401000	-2.682416000	-0.888564000
H	-5.761747000	-3.341163000	-1.766027000
H	-5.302421000	-3.303304000	-0.022342000
H	-6.551404000	-2.167414000	-0.648606000
O	-0.466581000	-0.216145000	-2.750950000
C	0.905592000	-0.463577000	-3.092804000
H	1.436908000	-0.943919000	-2.251687000
H	0.982374000	-1.087159000	-4.004802000
H	1.345470000	0.525898000	-3.280589000
C	3.818275000	0.915249000	-0.316131000
C	4.677919000	0.558451000	-1.372689000
C	4.170450000	0.458988000	0.970932000
C	5.831154000	-0.213931000	-1.172769000
C	5.313270000	-0.320950000	1.196405000
C	6.147740000	-0.656997000	0.119497000
F	4.390676000	0.942201000	-2.640839000
F	6.628465000	-0.542808000	-2.211971000
F	7.253515000	-1.399147000	0.326397000
F	5.627937000	-0.736034000	2.439543000
F	3.418194000	0.794093000	2.034799000
H	-0.570954000	-3.710562000	0.480862000

TS-2$_{Coor\text{-}E\text{-}C}$

Zero-point correction= 0.602301 (Hartree/Particle)
Thermal correction to Energy= 0.652828
Thermal correction to Enthalpy= 0.653772
Thermal correction to Gibbs Free Energy= 0.516204
Sum of electronic and zero-point Energies= -2890.832598
Sum of electronic and thermal Energies= -2890.782072
Sum of electronic and thermal Enthalpies= -2890.781127
Sum of electronic and thermal Free Energies= -2890.918696
E solvent= -2890.68785059

C	-2.002455000	2.710643000	0.862945000
C	-1.276292000	3.912934000	0.999630000
H	-0.316046000	4.025399000	0.483608000
C	-1.782561000	4.968292000	1.779470000
H	-1.206012000	5.896171000	1.872462000
C	-3.018702000	4.839543000	2.430832000
H	-3.411524000	5.663404000	3.037529000
C	-3.750253000	3.645385000	2.301642000
H	-4.717950000	3.536712000	2.805811000
C	-3.245083000	2.588782000	1.530075000
C	-2.648165000	1.104006000	-1.408772000
C	-3.229681000	2.304534000	-1.884450000
H	-2.953939000	3.254236000	-1.414031000
C	-4.164892000	2.311575000	-2.926729000
H	-4.590168000	3.260851000	-3.272390000
C	-4.549482000	1.098402000	-3.516047000
H	-5.278158000	1.081802000	-4.334730000
C	-3.998876000	-0.098422000	-3.045684000
H	-4.301395000	-1.050073000	-3.496101000
C	-3.050174000	-0.127590000	-1.995611000
C	-2.560720000	-1.485292000	-1.590125000
C	-3.467902000	-2.430646000	-1.046402000
C	-3.079361000	-3.764211000	-0.796547000
H	-3.788060000	-4.492655000	-0.393860000
C	-1.767364000	-4.156063000	-1.102984000
H	-1.462133000	-5.194788000	-0.928684000
C	-0.842479000	-3.251912000	-1.642016000
H	0.167530000	-3.586241000	-1.890476000
C	-1.244068000	-1.919992000	-1.895144000
C	0.166072000	1.796529000	-0.899826000
C	0.235600000	2.800480000	-1.886634000
H	-0.681577000	3.314472000	-2.198697000
C	1.454056000	3.137150000	-2.489622000
H	1.500403000	3.918767000	-3.255028000
C	2.621848000	2.457285000	-2.094878000
H	3.583638000	2.717747000	-2.552743000
C	2.592372000	1.451637000	-1.117931000
C	1.339951000	1.065413000	-0.511845000
C	0.813107000	-1.731059000	2.146803000
Ni	-0.264574000	-0.395451000	1.371524000
O	1.330251000	0.052669000	0.326994000
P	-1.357040000	1.235412000	-0.062934000
H	-3.824815000	1.662576000	1.433300000
C	0.236028000	-3.152651000	1.989967000
H	-0.455516000	-0.013993000	4.286871000
C	-1.032249000	-3.344018000	2.836406000
H	-0.801058000	-3.231449000	3.918008000
H	1.010148000	-3.875061000	2.304801000
C	-2.142876000	-2.369726000	2.497997000
O	-1.924089000	-1.303424000	1.881685000
O	1.873404000	-1.526018000	2.704696000
H	-1.418900000	-4.374882000	2.723318000
C	-3.550103000	-2.702930000	2.931858000
H	-3.575574000	-2.969526000	4.005845000
H	-4.224853000	-1.854849000	2.741295000
H	-3.911183000	-3.588850000	2.374540000

O	-4.733384000	-1.951775000	-0.788258000
C	-5.748187000	-2.900325000	-0.465658000
H	-5.838178000	-3.684000000	-1.243314000
H	-5.568572000	-3.386244000	0.514678000
H	-6.685726000	-2.325486000	-0.412694000
O	-0.445853000	-0.985215000	-2.484360000
C	0.913717000	-1.346621000	-2.767078000
H	1.444734000	-1.623856000	-1.838631000
H	0.960841000	-2.173087000	-3.502672000
H	1.376909000	-0.444649000	-3.189472000
C	3.832172000	0.730204000	-0.727276000
C	4.596175000	0.009651000	-1.665414000
C	4.296761000	0.722885000	0.603190000
C	5.762338000	-0.684662000	-1.311348000
C	5.453853000	0.031080000	0.986857000
C	6.191394000	-0.674283000	0.023521000
F	4.198815000	-0.052702000	-2.960566000
F	6.464278000	-1.370412000	-2.238864000
F	7.310480000	-1.335754000	0.378809000
F	5.875173000	0.052388000	2.267332000
F	3.642515000	1.423964000	1.551092000
C	0.536201000	1.325371000	2.942075000
C	-0.546609000	0.835068000	3.598833000
H	1.529119000	0.879881000	3.048524000
H	0.460663000	2.227656000	2.325785000
H	-0.008148000	-3.318730000	0.925112000
H	-1.533464000	1.304285000	3.512619000

2-Coor-E-C

Zero-point correction= 0.603493 (Hartree/Particle)
Thermal correction to Energy= 0.654451
Thermal correction to Enthalpy= 0.655395
Thermal correction to Gibbs Free Energy= 0.515163
Sum of electronic and zero-point Energies= -2890.856095
Sum of electronic and thermal Energies= -2890.805137
Sum of electronic and thermal Enthalpies= -2890.804193
Sum of electronic and thermal Free Energies= -2890.944425
E solvent= -2890.7053359

C	2.392169000	0.965375000	2.440770000
C	1.627139000	0.849249000	3.621055000
H	0.819140000	0.111224000	3.670429000
C	1.902547000	1.665256000	4.731249000
H	1.300436000	1.560219000	5.641346000
C	2.944066000	2.605261000	4.680860000
H	3.157182000	3.240072000	5.548314000
C	3.712972000	2.727791000	3.510408000
H	4.530803000	3.456313000	3.462686000
C	3.436554000	1.920123000	2.396853000
C	3.520861000	-0.852355000	0.461557000
C	4.523454000	-1.060497000	1.436160000
H	4.369712000	-0.687669000	2.454127000
C	5.716313000	-1.725330000	1.118025000
H	6.478303000	-1.877210000	1.891217000
C	5.925317000	-2.190100000	-0.188937000
H	6.855512000	-2.706883000	-0.451719000
C	4.935864000	-1.993061000	-1.161784000
H	5.090106000	-2.357437000	-2.183526000
C	3.725518000	-1.333163000	-0.861239000
C	2.712144000	-1.183182000	-1.955691000
C	2.857233000	-0.175883000	-2.941222000
C	1.946521000	-0.065866000	-4.010550000
H	2.045205000	0.722187000	-4.760687000
C	0.887862000	-0.981642000	-4.095351000
H	0.168060000	-0.891233000	-4.916555000
C	0.731204000	-2.010437000	-3.158372000
H	-0.097476000	-2.715330000	-3.256086000
C	1.655402000	-2.119787000	-2.097575000
C	0.782504000	-1.268321000	1.444900000
C	1.133851000	-2.435221000	2.146330000
H	2.181487000	-2.601786000	2.424512000
C	0.159836000	-3.390212000	2.470428000
H	0.428609000	-4.295654000	3.024547000
C	-1.169661000	-3.171786000	2.069603000
H	-1.938142000	-3.916136000	2.309652000
C	-1.552007000	-2.015483000	1.366794000
C	-0.566105000	-1.020988000	1.033225000
C	-1.221836000	2.249575000	-0.937098000
Ni	0.319843000	1.397460000	-0.226919000
O	-0.924013000	0.056446000	0.352951000
P	1.948042000	0.026631000	0.906555000
H	4.048813000	2.020153000	1.492079000
C	-2.220429000	2.753930000	0.103007000
H	-2.615306000	1.857855000	0.611576000
C	-3.334162000	3.609605000	-0.509158000
H	-4.110863000	3.823061000	0.253664000
H	-1.658017000	3.318638000	0.866849000
C	-2.831825000	4.953222000	-1.046857000
O	-1.694537000	5.362562000	-0.819198000
C	-3.818300000	5.761743000	-1.880290000
H	-3.436284000	6.782257000	-2.036190000
H	-3.947266000	5.272153000	-2.864790000
H	-4.816828000	5.796353000	-1.407134000
C	1.153960000	3.303584000	-0.160573000
C	1.641123000	2.597300000	-1.264043000
H	0.373736000	4.066902000	-0.277518000
H	1.713499000	3.328119000	0.783613000
H	1.183969000	2.738426000	-2.250938000
H	2.610197000	2.088747000	-1.246920000
O	-1.390241000	2.302940000	-2.139734000
H	-3.852087000	3.068041000	-1.322951000
O	3.936183000	0.665331000	-2.762279000
C	4.210029000	1.610996000	-3.799140000
H	4.357394000	1.109088000	-4.775041000
H	3.402358000	2.362643000	-3.894044000
H	5.141543000	2.115746000	-3.500363000
O	1.637499000	-3.120708000	-1.164513000
C	0.591715000	-4.093938000	-1.251516000
H	-0.405914000	-3.635674000	-1.126410000
H	0.634538000	-4.644100000	-2.212138000
H	0.771934000	-4.788324000	-0.417983000
C	-2.960171000	-1.839018000	0.923539000
C	-3.737230000	-0.733066000	1.316766000
C	-3.592907000	-2.777898000	0.084592000
C	-5.055931000	-0.545861000	0.881325000
C	-4.916385000	-2.626491000	-0.354738000
C	-5.652589000	-1.502274000	0.046259000
F	-2.913058000	-3.865021000	-0.353994000
F	-5.480063000	-3.544936000	-1.165325000
F	-6.923254000	-1.340728000	-0.368973000
F	-5.760380000	0.536376000	1.274632000
F	-3.218738000	0.197797000	2.149943000

TS-$2_{Ins-E-C}$

Zero-point correction= 0.603386 (Hartree/Particle)
Thermal correction to Energy= 0.653366
Thermal correction to Enthalpy= 0.654310
Thermal correction to Gibbs Free Energy= 0.514562
Sum of electronic and zero-point Energies= -2890.845945
Sum of electronic and thermal Energies= -2890.795965

Sum of electronic and thermal Enthalpies= -2890.795020
Sum of electronic and thermal Free Energies= -2890.934768
E solvent= -2890.70354431

C	2.336470000	0.300255000	2.654742000
C	1.562744000	0.089220000	3.815538000
H	0.677353000	-0.553924000	3.765238000
C	1.929262000	0.692234000	5.030311000
H	1.321667000	0.516938000	5.925710000
C	3.068641000	1.509288000	5.100625000
H	3.353244000	1.977197000	6.049914000
C	3.844722000	1.723419000	3.948111000
H	4.737953000	2.357081000	3.995996000
C	3.480928000	1.127135000	2.731835000
C	3.313850000	-1.202723000	0.361787000
C	4.216495000	-1.779584000	1.285931000
H	4.017004000	-1.691659000	2.359351000
C	5.373320000	-2.441932000	0.855147000
H	6.055738000	-2.883375000	1.590301000
C	5.649778000	-2.526782000	-0.517771000
H	6.553504000	-3.036487000	-0.871056000
C	4.762974000	-1.958995000	-1.440954000
H	4.972963000	-2.026924000	-2.513842000
C	3.582453000	-1.298828000	-1.031730000
C	2.689392000	-0.757701000	-2.106214000
C	3.103886000	0.338550000	-2.904196000
C	2.320597000	0.792921000	-3.984408000
H	2.636190000	1.643376000	-4.593187000
C	1.115177000	0.134710000	-4.272210000
H	0.497677000	0.488806000	-5.105093000
C	0.682511000	-0.962640000	-3.518952000
H	-0.260084000	-1.455529000	-3.766009000
C	1.475478000	-1.413782000	-2.442521000
C	0.544160000	-1.653885000	1.372050000
C	0.751600000	-2.887755000	2.012725000
H	1.757449000	-3.162878000	2.353606000
C	-0.320756000	-3.772229000	2.201101000
H	-0.168207000	-4.734482000	2.700602000
C	-1.602718000	-3.401299000	1.751837000
H	-2.447782000	-4.081813000	1.909780000
C	-1.839176000	-2.174872000	1.108936000
C	-0.743373000	-1.270152000	0.867658000
C	-0.880377000	2.391713000	-0.749953000
Ni	0.589829000	1.034799000	-0.093937000
O	-0.928421000	-0.159255000	0.188602000
P	1.796726000	-0.380601000	1.023668000
H	4.096936000	1.292629000	1.840327000
C	-1.466403000	2.914618000	0.570228000
H	-1.853708000	2.021818000	1.093257000
C	-2.622546000	3.903304000	0.340859000
H	-3.219905000	4.031440000	1.265115000
H	-0.675954000	3.361856000	1.197432000
C	-2.178922000	5.279969000	-0.150525000
O	-1.003459000	5.537176000	-0.414105000
C	-3.271284000	6.332370000	-0.302619000
H	-2.844789000	7.263312000	-0.706084000
H	-4.070588000	5.965938000	-0.973555000
H	-3.747443000	6.537864000	0.674762000
C	0.793063000	2.868661000	-1.108365000
C	1.929585000	2.384853000	-0.299199000
H	0.868734000	2.670202000	-2.186648000
H	0.482909000	3.909488000	-0.883619000
H	2.824807000	2.050640000	-0.839104000
H	2.141536000	2.893802000	0.653961000
O	-1.570240000	2.164858000	-1.733767000
H	-3.320198000	3.480049000	-0.408925000
O	4.297481000	0.920011000	-2.527659000
C	4.790739000	1.991232000	-3.335847000
H	4.950290000	1.669523000	-4.383392000
H	4.106817000	2.862178000	-3.321782000
H	5.754986000	2.278176000	-2.889257000
O	1.178198000	-2.507755000	-1.678588000
C	-0.052687000	-3.193196000	-1.941704000
H	-0.920256000	-2.518964000	-1.830369000
H	-0.050047000	-3.640293000	-2.955084000
H	-0.112391000	-3.986376000	-1.182924000
C	-3.195069000	-1.804180000	0.629144000
C	-3.818694000	-0.606614000	1.031550000
C	-3.919072000	-2.615699000	-0.266480000
C	-5.078446000	-0.219301000	0.557130000
C	-5.189058000	-2.261557000	-0.745517000
C	-5.770711000	-1.054106000	-0.332926000
F	-3.379686000	-3.772722000	-0.720076000
F	-5.845618000	-3.063424000	-1.609035000
F	-6.987954000	-0.698640000	-0.786690000
F	-5.639475000	0.938853000	0.965216000
F	-3.211955000	0.206481000	1.926477000

2-cycle5-T

Zero-point correction= 0.605164 (Hartree/Particle)
Thermal correction to Energy= 0.655251
Thermal correction to Enthalpy= 0.656195
Thermal correction to Gibbs Free Energy= 0.515316
Sum of electronic and zero-point Energies= -2890.876108
Sum of electronic and thermal Energies= -2890.826021
Sum of electronic and thermal Enthalpies= -2890.825077
Sum of electronic and thermal Free Energies= -2890.965956
E solvent= -2890.73161977

C	2.268375000	-0.891410000	2.610988000
C	1.471932000	-1.294554000	3.704246000
H	0.499113000	-1.763680000	3.520840000
C	1.925394000	-1.105261000	5.019500000
H	1.299340000	-1.428810000	5.859159000
C	3.174629000	-0.510357000	5.259697000
H	3.527510000	-0.365664000	6.287232000
C	3.972245000	-0.104448000	4.176242000
H	4.950710000	0.356512000	4.355414000
C	3.523465000	-0.290707000	2.859863000
C	3.052288000	-1.710863000	-0.059708000
C	3.830067000	-2.705881000	0.579729000
H	3.612940000	-2.970829000	1.620205000
C	4.886990000	-3.343414000	-0.081429000
H	5.469891000	-4.114504000	0.434955000
C	5.191757000	-2.981371000	-1.402385000
H	6.015455000	-3.469677000	-1.935753000
C	4.441071000	-1.986631000	-2.039336000
H	4.682199000	-1.695805000	-3.067425000
C	3.363120000	-1.335399000	-1.395203000
C	2.644493000	-0.279450000	-2.177904000
C	3.321519000	0.913712000	-2.538876000
C	2.713470000	1.876867000	-3.371771000
H	3.242359000	2.787886000	-3.663127000
C	1.412018000	1.641131000	-3.842650000
H	0.935168000	2.383049000	-4.493695000
C	0.712580000	0.476561000	-3.505325000
H	-0.298409000	0.316932000	-3.885866000
C	1.334729000	-0.489224000	-2.682822000
C	0.293817000	-2.274095000	0.913859000
C	0.414150000	-3.640127000	1.222652000
H	1.394770000	-4.063662000	1.471365000
C	-0.719005000	-4.466333000	1.203217000

H	-0.635806000	-5.530716000	1.445822000
C	-1.970837000	-3.909097000	0.880908000
H	-2.860377000	-4.549631000	0.878677000
C	-2.123033000	-2.546891000	0.570395000
C	-0.964108000	-1.689424000	0.546875000
C	0.012947000	3.335535000	-0.227290000
Ni	0.492255000	0.715144000	0.321024000
O	-1.058153000	-0.432251000	0.178358000
P	1.618485000	-1.020929000	0.885837000
H	4.157679000	0.019378000	2.021631000
C	-0.737154000	4.624701000	-0.461953000
H	-0.149553000	5.313436000	-1.094841000
C	-1.037692000	5.322675000	0.883563000
H	-0.111086000	5.447120000	1.482969000
H	-1.686000000	4.403662000	-0.979684000
C	-1.646424000	6.721215000	0.693366000
O	-1.654126000	7.276875000	-0.399921000
C	-2.236937000	7.367957000	1.940893000
H	-3.178033000	6.851988000	2.212378000
H	-1.558396000	7.269483000	2.808054000
H	-2.454488000	8.429480000	1.747934000
C	1.509490000	3.267081000	-0.146301000
C	1.921881000	1.989611000	0.615012000
H	1.850915000	3.197589000	-1.203817000
H	1.941013000	4.214375000	0.245526000
H	2.939222000	1.671595000	0.331138000
H	1.897396000	2.155552000	1.711376000
O	-0.624987000	2.260795000	-0.086819000
H	-1.710996000	4.702952000	1.506472000
O	4.585741000	1.053641000	-2.009694000
C	5.333596000	2.210908000	-2.382738000
H	5.491670000	2.260636000	-3.478033000
H	4.841040000	3.143853000	-2.044499000
H	6.306266000	2.110425000	-1.877244000
O	0.767269000	-1.685911000	-2.349545000
C	-0.580689000	-1.926392000	-2.780069000
H	-1.261203000	-1.153714000	-2.380455000
H	-0.643128000	-1.965400000	-3.885108000
H	-0.854220000	-2.903427000	-2.357293000
C	-3.450752000	-1.979389000	0.226134000
C	-3.966454000	-0.841583000	0.882736000
C	-4.259148000	-2.540939000	-0.783497000
C	-5.205535000	-0.281090000	0.541478000
C	-5.508637000	-2.008649000	-1.132658000
C	-5.982626000	-0.868625000	-0.467822000
F	-3.826039000	-3.621894000	-1.476693000
F	-6.247684000	-2.573246000	-2.111214000
F	-7.180573000	-0.343760000	-0.793086000
F	-5.668001000	0.806888000	1.193013000
F	-3.284511000	-0.271740000	1.894727000

TS-2_{Isom}

Zero-point correction= 0.547766 (Hartree/Particle)
Thermal correction to Energy= 0.595533
Thermal correction to Enthalpy= 0.596477
Thermal correction to Gibbs Free Energy= 0.462806
Sum of electronic and zero-point Energies= -2812.285710
Sum of electronic and thermal Energies= -2812.237943
Sum of electronic and thermal Enthalpies= -2812.236999
Sum of electronic and thermal Free Energies= -2812.370670
E solvent= -2812.11320208

C	-2.859313000	2.006830000	-1.054439000
C	-2.331971000	3.265176000	-1.421376000
H	-1.256840000	3.377130000	-1.596980000
C	-3.181444000	4.374466000	-1.567257000
H	-2.757223000	5.343717000	-1.852118000
C	-4.560709000	4.243976000	-1.344912000
H	-5.221267000	5.110840000	-1.459375000
C	-5.091022000	2.998305000	-0.965595000
H	-6.166780000	2.890600000	-0.784540000
C	-4.248442000	1.888073000	-0.814251000
C	-2.683848000	-0.865546000	-1.414927000
C	-3.374486000	-0.716739000	-2.639567000
H	-3.390145000	0.259731000	-3.134985000
C	-4.051775000	-1.796081000	-3.219205000
H	-4.583142000	-1.662000000	-4.168195000
C	-4.044977000	-3.044341000	-2.576065000
H	-4.572078000	-3.897622000	-3.017912000
C	-3.359696000	-3.199153000	-1.365135000
H	-3.350537000	-4.172616000	-0.862437000
C	-2.667058000	-2.121899000	-0.765636000
C	-1.947324000	-2.409069000	0.520695000
C	-2.698301000	-2.595269000	1.720127000
C	-2.111861000	-3.170270000	2.862931000
H	-2.682185000	-3.303333000	3.784842000
C	-0.762504000	-3.556103000	2.812890000
H	-0.300658000	-3.990506000	3.706724000
C	0.010289000	-3.392262000	1.658348000
H	1.054362000	-3.712031000	1.648626000
C	-0.591241000	-2.854426000	0.496182000
C	-0.265306000	0.782652000	-1.754233000
C	-0.242801000	1.219465000	-3.091826000
H	-1.177567000	1.511172000	-3.586444000
C	0.967067000	1.286994000	-3.795879000
H	0.987184000	1.625828000	-4.836711000
C	2.160191000	0.924260000	-3.144263000
H	3.111781000	0.988223000	-3.685257000
C	2.173129000	0.498118000	-1.806709000
C	0.937234000	0.382970000	-1.068319000
Ni	-0.791104000	0.140232000	1.282561000
O	0.923663000	-0.065758000	0.162617000
P	-1.752797000	0.574116000	-0.723847000
H	-4.670055000	0.921306000	-0.517024000
C	-1.006886000	2.059596000	1.818010000
H	-1.241064000	2.627440000	0.906990000
C	0.347893000	2.454963000	2.379808000
H	1.160130000	1.906757000	1.859163000
H	-1.845103000	2.146312000	2.526533000
C	0.693878000	3.949688000	2.187460000
O	0.038034000	4.701699000	1.475509000
H	0.456629000	2.193103000	3.449806000
C	1.956480000	4.413021000	2.910760000
H	2.812603000	3.773184000	2.626989000
H	2.171470000	5.461572000	2.653300000
H	1.832632000	4.319400000	4.006105000
O	-4.007598000	-2.197184000	1.644838000
C	-4.813028000	-2.348483000	2.817380000
H	-4.892978000	-3.410495000	3.120737000
H	-4.414150000	-1.753882000	3.661399000
H	-5.809121000	-1.971052000	2.541233000
O	0.016992000	-2.787992000	-0.718266000
C	1.417100000	-3.098653000	-0.778324000
H	1.983007000	-2.449640000	-0.088110000
H	1.597823000	-4.166843000	-0.549305000
H	1.722862000	-2.884417000	-1.812213000
C	3.451325000	0.146594000	-1.134589000
C	4.309703000	-0.851536000	-1.637039000
C	3.874353000	0.797385000	0.041413000
C	5.515203000	-1.196983000	-1.007542000
C	5.066218000	0.465891000	0.699166000
C	5.894787000	-0.534848000	0.169156000

F	3.966455000	-1.540878000	-2.751830000
F	6.302060000	-2.167647000	-1.515954000
F	7.048292000	-0.855906000	0.785384000
F	5.433691000	1.117178000	1.822341000
F	3.139360000	1.808404000	0.559289000
C	-0.811432000	-0.242539000	3.013082000
O	-0.913842000	-0.382739000	4.169074000

1-Coor-CO-C

Zero-point correction= 0.548958 (Hartree/Particle)
Thermal correction to Energy= 0.597192
Thermal correction to Enthalpy= 0.598136
Thermal correction to Gibbs Free Energy= 0.462517
Sum of electronic and zero-point Energies= -2812.317787
Sum of electronic and thermal Energies= -2812.269553
Sum of electronic and thermal Enthalpies= -2812.268609
Sum of electronic and thermal Free Energies= -2812.404228
E solvent= -2812.14917993

C	2.464534000	-0.537855000	2.558236000
C	1.716479000	-1.059911000	3.634628000
H	0.775774000	-1.585240000	3.437878000
C	2.178091000	-0.914258000	4.953247000
H	1.589744000	-1.328015000	5.780315000
C	3.385922000	-0.247399000	5.211632000
H	3.743970000	-0.135659000	6.241298000
C	4.134082000	0.278506000	4.143922000
H	5.077836000	0.801131000	4.337589000
C	3.676345000	0.139848000	2.825776000
C	3.229228000	-1.253607000	-0.162575000
C	4.116844000	-2.184392000	0.424861000
H	3.976807000	-2.479305000	1.470802000
C	5.187197000	-2.717925000	-0.304786000
H	5.863569000	-3.440049000	0.166307000
C	5.387583000	-2.315081000	-1.634332000
H	6.224518000	-2.720267000	-2.214502000
C	4.515031000	-1.390281000	-2.222391000
H	4.670387000	-1.072814000	-3.259155000
C	3.422007000	-0.848175000	-1.511077000
C	2.532556000	0.126022000	-2.221776000
C	2.993229000	1.436694000	-2.511825000
C	2.202742000	2.335382000	-3.257397000
H	2.550780000	3.348292000	-3.472022000
C	0.946942000	1.914600000	-3.718349000
H	0.328471000	2.611753000	-4.294701000
C	0.463953000	0.625465000	-3.459622000
H	-0.515598000	0.323354000	-3.836622000
C	1.261734000	-0.269758000	-2.715179000
C	0.472329000	-1.825246000	0.812494000
C	0.600278000	-3.209644000	1.022185000
H	1.588081000	-3.640806000	1.225152000
C	-0.528685000	-4.038552000	0.956904000
H	-0.437811000	-5.117171000	1.120672000
C	-1.784953000	-3.465961000	0.687136000
H	-2.673187000	-4.107172000	0.644790000
C	-1.942503000	-2.085163000	0.474770000
C	-0.790639000	-1.226131000	0.508034000
Ni	0.589799000	1.229197000	0.279873000
O	-0.917323000	0.063192000	0.231918000
P	1.820142000	-0.601800000	0.831232000
H	4.268692000	0.552239000	2.000545000
C	-0.679493000	2.726045000	-0.115779000
H	-0.236980000	3.652332000	-0.510814000
C	-1.528763000	2.965978000	1.120014000
H	-0.922316000	3.368399000	1.960205000
H	-1.219904000	2.198252000	-0.918788000
C	-2.670280000	3.979873000	0.894984000
O	-2.737986000	4.688793000	-0.104236000
H	-1.964275000	2.020790000	1.493575000
C	-3.724819000	4.035476000	1.998259000
H	-3.258306000	4.078548000	2.999883000
H	-4.382339000	4.906008000	1.848665000
H	-4.332591000	3.111631000	1.968903000
O	4.234491000	1.742790000	-2.006918000
C	4.753556000	3.048151000	-2.276935000
H	4.862884000	3.224547000	-3.364837000
H	4.114745000	3.836846000	-1.836900000
H	5.745306000	3.074452000	-1.800476000
O	0.909834000	-1.562995000	-2.438249000
C	-0.379932000	-2.010169000	-2.872764000
H	-1.185781000	-1.387878000	-2.444470000
H	-0.451629000	-2.009430000	-3.978022000
H	-0.477390000	-3.037268000	-2.493433000
C	-3.280292000	-1.508855000	0.176637000
C	-4.046924000	-1.944708000	-0.922283000
C	-3.851865000	-0.501895000	0.979530000
C	-5.312397000	-1.415466000	-1.216367000
C	-5.110380000	0.048986000	0.704906000
C	-5.846833000	-0.409975000	-0.397516000
F	-3.556094000	-2.891971000	-1.756813000
F	-6.009422000	-1.855277000	-2.282909000
F	-7.058854000	0.108466000	-0.665117000
F	-5.629112000	1.009172000	1.502617000
F	-3.194060000	-0.047301000	2.068967000
C	1.763492000	2.479695000	0.405967000
O	2.552930000	3.329757000	0.540426000

Catalyst 3

1-cycle5-T

Zero-point correction= 0.555759 (Hartree/Particle)
Thermal correction to Energy= 0.600372
Thermal correction to Enthalpy= 0.601317
Thermal correction to Gibbs Free Energy= 0.475510
Sum of electronic and zero-point Energies= -2700.969318
Sum of electronic and thermal Energies= -2700.924704
Sum of electronic and thermal Enthalpies= -2700.923760
Sum of electronic and thermal Free Energies= -2701.049567
E solvent= -2700.77455572

C	-2.801464000	1.872774000	0.476753000
C	-2.217808000	3.056107000	1.019625000
C	-3.077915000	4.048094000	1.546208000
H	-2.623464000	4.960136000	1.950160000
C	-4.466639000	3.874174000	1.602131000
H	-5.100608000	4.660185000	2.028010000
C	-5.030170000	2.678390000	1.134505000
H	-6.111488000	2.510005000	1.192568000
C	-4.202074000	1.695304000	0.575428000
C	-2.958884000	-0.234799000	-1.536914000
C	-3.706517000	0.669753000	-2.330337000
H	-3.614806000	1.745269000	-2.146053000
C	-4.590176000	0.228498000	-3.322342000
H	-5.149271000	0.957185000	-3.919927000
C	-4.753866000	-1.148902000	-3.535093000
H	-5.441290000	-1.515387000	-4.305891000
C	-4.017718000	-2.057056000	-2.767704000
H	-4.115703000	-3.131924000	-2.956863000
C	-3.101427000	-1.635482000	-1.770643000
C	-2.339454000	-2.708517000	-1.068314000

C	-3.023198000	-3.838683000	-0.560893000
C	-2.320269000	-4.909711000	0.013277000
H	-2.870692000	-5.778139000	0.394343000
C	-0.915454000	-4.871587000	0.089309000
H	-0.364111000	-5.714965000	0.521477000
C	-0.223498000	-3.755059000	-0.405132000
H	0.869026000	-3.706402000	-0.364739000
C	-0.927931000	-2.677781000	-0.973208000
C	-0.389856000	1.133221000	-1.236417000
C	-0.424610000	2.037250000	-2.312901000
H	-1.377286000	2.475335000	-2.633878000
C	0.760218000	2.384667000	-2.974232000
H	0.743887000	3.093030000	-3.808929000
C	1.974951000	1.802430000	-2.563705000
H	2.901368000	2.063909000	-3.088206000
C	2.037607000	0.876609000	-1.510928000
C	0.835877000	0.529944000	-0.794557000
C	-0.540936000	-2.439200000	3.256464000
Ni	-0.782415000	-0.749168000	1.133247000
O	0.869497000	-0.297813000	0.225123000
P	-1.802598000	0.496664000	-0.274046000
H	-4.658432000	0.780211000	0.184872000
C	0.055624000	-3.242839000	4.383103000
C	-2.014664000	-2.433434000	2.964042000
C	-2.365635000	-1.151543000	2.177776000
H	-2.191110000	-3.323477000	2.320964000
H	-2.613434000	-2.601183000	3.885433000
H	-3.307667000	-1.281449000	1.618008000
H	-2.484328000	-0.286238000	2.860399000
O	0.201956000	-1.751890000	2.513784000
H	-0.294038000	-4.291005000	4.336782000
H	1.155088000	-3.209793000	4.347152000
H	-0.292967000	-2.837545000	5.352706000
H	-4.118063000	-3.870228000	-0.613586000
H	-0.364865000	-1.854509000	-1.421181000
C	-0.748106000	3.309111000	1.122797000
C	0.079010000	2.453338000	1.881318000
C	-0.180211000	4.466797000	0.545721000
H	-0.351306000	1.558170000	2.347752000
H	-0.815377000	5.138322000	-0.044195000
C	1.445167000	2.732759000	2.034743000
C	1.186950000	4.740502000	0.693171000
H	2.073207000	2.046971000	2.613746000
H	1.617123000	5.630098000	0.218854000
C	2.005038000	3.871840000	1.435136000
H	3.075929000	4.078463000	1.537558000
C	3.325395000	0.268984000	-1.091535000
C	3.493140000	-1.128075000	-1.002791000
C	4.440624000	1.056437000	-0.739410000
C	4.688700000	-1.714317000	-0.566294000
C	5.653485000	0.495033000	-0.313509000
C	5.776793000	-0.898713000	-0.222181000
F	2.496958000	-1.959441000	-1.380754000
F	4.808917000	-3.057222000	-0.496904000
F	6.935634000	-1.451795000	0.186999000
F	6.694984000	1.285300000	0.022946000
F	4.361178000	2.406648000	-0.782687000

TS_{Isom}

Zero-point correction= 0.607660 (Hartree/Particle)
Thermal correction to Energy= 0.655546
Thermal correction to Enthalpy= 0.656490
Thermal correction to Gibbs Free Energy= 0.522771
Sum of electronic and zero-point Energies= -2779.478131
Sum of electronic and thermal Energies= -2779.430245
Sum of electronic and thermal Enthalpies= -2779.429301
Sum of electronic and thermal Free Energies= -2779.563021
E solvent= -2779.29483093

C	2.719495000	-1.327161000	0.306715000
C	2.543807000	-2.716541000	0.037369000
C	3.478136000	-3.628793000	0.596825000
H	3.331325000	-4.696168000	0.395421000
C	4.542596000	-3.216030000	1.403867000
H	5.234998000	-3.956697000	1.819743000
C	4.700476000	-1.849698000	1.686685000
H	5.519083000	-1.500163000	2.325833000
C	3.794012000	-0.931529000	1.146694000
C	3.014393000	0.995059000	-1.370059000
C	3.938818000	0.192863000	-2.078230000
H	3.903022000	-0.895443000	-1.958120000
C	4.915314000	0.756116000	-2.910488000
H	5.613192000	0.106560000	-3.450802000
C	4.990570000	2.151026000	-3.041622000
H	5.745344000	2.608862000	-3.690944000
C	4.088581000	2.960626000	-2.341765000
H	4.132547000	4.049417000	-2.459109000
C	3.086869000	2.415032000	-1.502035000
C	2.183381000	3.368366000	-0.795829000
C	2.732328000	4.496896000	-0.141020000
C	1.906046000	5.429311000	0.504706000
H	2.354506000	6.291748000	1.011238000
C	0.510944000	5.255834000	0.507808000
H	-0.134229000	5.986874000	1.008099000
C	-0.050344000	4.144209000	-0.140682000
H	-1.134305000	3.996301000	-0.156188000
C	0.776882000	3.207599000	-0.786306000
C	0.380404000	-0.213623000	-1.404730000
C	0.502031000	-0.656415000	-2.735903000
H	1.498398000	-0.833718000	-3.157103000
C	-0.635222000	-0.877782000	-3.520925000
H	-0.538768000	-1.230642000	-4.552579000
C	-1.907104000	-0.640109000	-2.969273000
H	-2.803027000	-0.811505000	-3.577428000
C	-2.066717000	-0.161786000	-1.659200000
C	-0.909097000	0.068913000	-0.836066000
Ni	0.413541000	1.025907000	1.505272000
O	-1.072882000	0.532640000	0.391285000
P	1.760633000	0.170560000	-0.269535000
H	3.925622000	0.134774000	1.369318000
C	-0.239576000	-0.377582000	2.735308000
H	-1.166032000	0.002379000	3.193785000
C	0.805984000	-0.827963000	3.742082000
H	1.026960000	-0.039085000	4.480524000
H	-0.484464000	-1.148376000	1.988703000
C	0.261464000	-2.041927000	4.542798000
O	-0.280780000	-1.885358000	5.629969000
H	1.740052000	-1.119458000	3.226059000
C	0.437542000	-3.411875000	3.901833000
H	1.513485000	-3.669212000	3.866736000
H	-0.103687000	-4.176118000	4.480245000
H	0.083927000	-3.407864000	2.854392000
C	0.330097000	2.334078000	3.038069000
C	1.676037000	2.093310000	2.681176000
H	-0.204990000	3.191003000	2.608873000
H	-0.104338000	1.917380000	3.953306000
H	2.199314000	2.787251000	2.013170000
H	2.317279000	1.458222000	3.303308000
H	0.320169000	2.385516000	-1.346447000
H	3.820579000	4.628509000	-0.122506000
C	1.451615000	-3.330997000	-0.779181000
C	0.088991000	-3.161677000	-0.454004000

C	1.783842000	-4.196805000	-1.845801000
H	-0.187523000	-2.507895000	0.379592000
H	2.838621000	-4.339142000	-2.110370000
C	-0.913175000	-3.820433000	-1.180667000
C	0.783574000	-4.860037000	-2.572819000
H	-1.964831000	-3.650092000	-0.929103000
H	1.062565000	-5.520952000	-3.401646000
C	-0.567955000	-4.672465000	-2.242589000
H	-1.351150000	-5.186019000	-2.811631000
C	-3.420464000	0.091873000	-1.098456000
C	-4.390426000	-0.926400000	-1.015310000
C	-3.799771000	1.358977000	-0.613338000
C	-5.664825000	-0.709800000	-0.470927000
C	-5.061333000	1.601980000	-0.053459000
C	-6.000875000	0.562138000	0.015016000
F	-2.946621000	2.401941000	-0.705556000
F	-5.385248000	2.830081000	0.399924000
F	-7.219891000	0.785288000	0.540602000
F	-6.561474000	-1.714595000	-0.401910000
F	-4.103106000	-2.178155000	-1.449348000

TS-2$_{Coor\text{-}E\text{-}C}$

Zero-point correction= 0.617237 (Hartree/Particle)
Thermal correction to Energy= 0.667437
Thermal correction to Enthalpy= 0.668382
Thermal correction to Gibbs Free Energy= 0.530968
Sum of electronic and zero-point Energies= -2892.811463
Sum of electronic and thermal Energies= -2892.761262
Sum of electronic and thermal Enthalpies= -2892.760318
Sum of electronic and thermal Free Energies= -2892.897731
E solvent= -2892.62224065

C	2.888728000	1.427015000	-0.779686000
C	2.832795000	2.849092000	-0.867908000
C	3.846247000	3.512349000	-1.609465000
H	3.792510000	4.604744000	-1.682407000
C	4.873026000	2.823405000	-2.262604000
H	5.629743000	3.376701000	-2.830292000
C	4.907164000	1.421077000	-2.201612000
H	5.692260000	0.857195000	-2.718253000
C	3.921342000	0.744530000	-1.475218000
C	2.950679000	-0.426440000	1.432309000
C	4.100413000	0.329621000	1.759779000
H	4.266820000	1.288842000	1.259946000
C	5.045329000	-0.119805000	2.692991000
H	5.919323000	0.500138000	2.923288000
C	4.863367000	-1.360061000	3.319164000
H	5.593684000	-1.732512000	4.046523000
C	3.723227000	-2.116343000	3.020945000
H	3.554778000	-3.070069000	3.534424000
C	2.745322000	-1.674481000	2.097598000
C	1.550054000	-2.561873000	1.938878000
C	1.731154000	-3.936206000	1.652959000
H	2.743109000	-4.308518000	1.451759000
C	0.641416000	-4.820628000	1.637806000
H	0.806488000	-5.884855000	1.429255000
C	-0.655396000	-4.348668000	1.911748000
H	-1.505294000	-5.041215000	1.915293000
C	-0.849651000	-2.987372000	2.199521000
H	-1.849630000	-2.600345000	2.425041000
C	0.242661000	-2.104842000	2.216695000
H	0.084450000	-1.056534000	2.482907000
C	0.449862000	1.005529000	1.072533000
C	0.604759000	1.700537000	2.286979000
H	1.612690000	1.885965000	2.676886000
C	-0.515372000	2.141977000	3.001901000
H	-0.395944000	2.682869000	3.946089000
C	-1.801560000	1.873806000	2.494520000
H	-2.685373000	2.205034000	3.053346000
C	-1.989267000	1.184916000	1.287217000
C	-0.851739000	0.718681000	0.539028000
C	-1.032292000	-1.961598000	-2.184631000
Ni	0.382102000	-1.023680000	-1.377973000
O	-1.047743000	0.048168000	-0.577063000
P	1.783520000	0.216496000	0.118333000
H	3.955857000	-0.351393000	-1.424464000
C	-1.104169000	-3.432878000	-1.729712000
H	-1.074088000	-3.465400000	-0.625587000
C	0.060414000	-4.259546000	-2.302101000
H	-0.012051000	-4.314921000	-3.410216000
H	-2.064455000	-3.855710000	-2.076289000
C	1.438728000	-3.721089000	-1.967974000
O	1.624396000	-2.538772000	-1.604923000
O	-1.831445000	-1.479471000	-2.963237000
H	-0.003088000	-5.307182000	-1.950799000
C	2.620422000	-4.653569000	-2.092686000
H	2.595745000	-5.194932000	-3.057302000
H	3.565558000	-4.097439000	-1.999403000
H	2.573453000	-5.420043000	-1.294908000
C	2.170332000	4.807696000	0.562222000
C	1.217656000	5.705696000	1.067507000
C	1.783620000	3.730578000	-0.266878000
H	1.537902000	6.528900000	1.716849000
C	-0.139377000	5.546513000	0.744486000
C	0.416385000	3.586925000	-0.587472000
H	-0.885416000	6.246514000	1.138053000
H	0.089897000	2.767721000	-1.235834000
C	-0.536417000	4.484114000	-0.083689000
H	-1.591250000	4.338395000	-0.337925000
H	3.229236000	4.930448000	0.820595000
C	1.304785000	-0.423974000	-3.765402000
C	0.540869000	0.590849000	-3.291354000
H	0.859452000	-1.266304000	-4.307255000
H	2.395900000	-0.416476000	-3.664075000
H	-0.545518000	0.601698000	-3.422699000
H	1.004590000	1.465168000	-2.822332000
C	-3.351879000	0.875262000	0.778220000
C	-3.817277000	1.384433000	-0.449163000
C	-4.227721000	0.029843000	1.484417000
C	-5.087882000	1.075037000	-0.953593000
C	-5.506653000	-0.292719000	1.007580000
C	-5.937756000	0.234498000	-0.218359000
F	-3.832325000	-0.532658000	2.653123000
F	-6.316998000	-1.113331000	1.709356000
F	-7.164166000	-0.065471000	-0.688575000
F	-5.504884000	1.585806000	-2.129399000
F	-3.037489000	2.213253000	-1.177299000

Catalyst 4

1-cycle5-T

Zero-point correction= 0.576220 (Hartree/Particle)
Thermal correction to Energy= 0.622416
Thermal correction to Enthalpy= 0.623360
Thermal correction to Gibbs Free Energy= 0.495138
Sum of electronic and zero-point Energies= -2702.165275
Sum of electronic and thermal Energies= -2702.119079
Sum of electronic and thermal Enthalpies= -2702.118134
Sum of electronic and thermal Free Energies= -2702.246356
E solvent= -2701.98349491

C	-2.025122000	2.654185000	-0.106123000
C	-1.120327000	3.665830000	-0.491667000
H	-0.207141000	3.398533000	-1.033775000
C	-1.387174000	5.011191000	-0.187372000
H	-0.675994000	5.785390000	-0.497938000
C	-2.556204000	5.364122000	0.504561000
H	-2.762921000	6.414584000	0.739084000
C	-3.462344000	4.361978000	0.893042000
H	-4.380384000	4.627939000	1.430192000
C	-3.199667000	3.017499000	0.593496000
C	-0.242598000	0.861568000	-1.585827000
C	-0.311513000	1.129915000	-2.962906000
H	-1.284999000	1.295348000	-3.441855000
C	0.867410000	1.182012000	-3.722397000
H	0.829562000	1.388836000	-4.796916000
C	2.106733000	0.991599000	-3.081260000
H	3.029570000	1.059673000	-3.668605000
C	2.206697000	0.730026000	-1.703452000
C	1.003029000	0.608713000	-0.916204000
C	-0.219614000	-0.272744000	4.053738000
Ni	-0.588940000	0.205029000	1.391364000
O	1.042914000	0.276715000	0.351125000
P	-1.605808000	0.865519000	-0.381804000
C	0.461616000	-0.403821000	5.391392000
C	-1.707929000	-0.411400000	3.887912000
C	-2.134728000	0.269742000	2.568719000
H	-1.904896000	-1.505472000	3.843264000
H	-2.247712000	-0.058241000	4.793137000
H	-3.066829000	-0.173719000	2.177367000
H	-2.301560000	1.352142000	2.735094000
O	0.459848000	-0.048345000	3.021933000
H	0.118723000	-1.314210000	5.917263000
H	1.555463000	-0.423666000	5.271909000
H	0.181848000	0.454579000	6.032696000
C	-1.649643000	-2.379535000	-0.168146000
C	-0.974890000	-2.351633000	-1.408346000
C	-0.945993000	-2.823688000	0.976404000
H	-1.518401000	-2.039364000	-2.307700000
H	-1.469002000	-2.864894000	1.939890000
C	0.363985000	-2.771137000	-1.521193000
C	0.397773000	-3.230621000	0.895220000
C	1.033743000	-3.201955000	-0.360985000
H	2.081284000	-3.522342000	-0.436504000
C	3.524719000	0.540750000	-1.047727000
C	4.485836000	-0.365200000	-1.541304000
C	3.883256000	1.257724000	0.114641000
C	5.724007000	-0.570846000	-0.916691000
C	5.109157000	1.061138000	0.766283000
C	6.036315000	0.145593000	0.247387000
F	4.223752000	-1.102659000	-2.650471000
F	6.608468000	-1.458196000	-1.419449000
F	7.222090000	-0.040829000	0.860341000
F	5.414759000	1.765062000	1.876335000
F	3.055387000	2.190661000	0.622031000
H	-7.011001000	-2.389480000	-0.768542000
C	-5.939083000	-2.317279000	-0.547885000
C	-5.301240000	-3.284814000	0.208558000
C	-3.897014000	-3.222786000	0.371710000
C	-3.126625000	-2.169901000	-0.136995000
C	-3.795866000	-1.055751000	-0.771859000
C	-5.212337000	-1.204356000	-1.063609000
C	-5.883004000	-0.237055000	-1.868983000
C	-5.216666000	0.877920000	-2.342654000
C	-3.885591000	1.117679000	-1.920205000
C	-3.179212000	0.206145000	-1.126331000
H	-5.860700000	-4.133654000	0.617317000
H	-3.374732000	-4.065926000	0.837997000
H	-5.726300000	1.610396000	-2.978104000
H	-6.943567000	-0.396424000	-2.099896000
C	1.057191000	-2.789798000	-2.868425000
H	0.551686000	-2.124009000	-3.588350000
H	2.105220000	-2.456674000	-2.784638000
H	1.065334000	-3.809782000	-3.300559000
C	1.161842000	-3.645680000	2.135660000
H	1.897102000	-4.439623000	1.913443000
H	1.722128000	-2.786595000	2.551775000
H	0.485826000	-4.019690000	2.925752000
H	-3.920011000	2.248452000	0.893662000
H	-3.425927000	2.078999000	-2.172226000

TS_{Isom}

Zero-point correction= 0.628854 (Hartree/Particle)
Thermal correction to Energy= 0.677774
Thermal correction to Enthalpy= 0.678719
Thermal correction to Gibbs Free Energy= 0.545467
Sum of electronic and zero-point Energies= -2780.676367
Sum of electronic and thermal Energies= -2780.627446
Sum of electronic and thermal Enthalpies= -2780.626502
Sum of electronic and thermal Free Energies= -2780.759754
E solvent= -2780.51311482

C	-2.143269000	2.007167000	-1.161938000
C	-1.386035000	2.679306000	-2.144377000
H	-0.552124000	2.163932000	-2.632939000
C	-1.704661000	4.000464000	-2.511398000
H	-1.111877000	4.503589000	-3.284572000
C	-2.778972000	4.667402000	-1.902647000
H	-3.026429000	5.695210000	-2.191146000
C	-3.538171000	4.006371000	-0.919165000
H	-4.379242000	4.518422000	-0.437366000
C	-3.219689000	2.692654000	-0.548150000
C	-0.341503000	-0.235101000	-1.626672000
C	-0.448941000	-0.604551000	-2.979386000
H	-1.436742000	-0.624844000	-3.457019000
C	0.691084000	-0.955558000	-3.718314000
H	0.606101000	-1.243383000	-4.771368000
C	1.950816000	-0.906403000	-3.096169000
H	2.849521000	-1.148554000	-3.675013000
C	2.097643000	-0.534034000	-1.748619000
C	0.931540000	-0.229539000	-0.957661000
Ni	-0.410056000	0.624770000	1.417238000
O	1.065147000	0.020165000	0.331530000
P	-1.717693000	0.303293000	-0.550852000
C	0.475149000	2.393999000	1.296617000
H	1.420531000	2.241232000	1.840560000
C	-0.336109000	3.582008000	1.772947000
H	-0.611621000	3.525547000	2.838570000
H	0.663900000	2.412012000	0.213526000
C	0.420541000	4.931352000	1.608843000
O	0.477270000	5.725562000	2.539918000
H	-1.267306000	3.667982000	1.176682000
C	1.059530000	5.211312000	0.257736000
H	0.348039000	5.006330000	-0.563294000
H	1.408452000	6.254613000	0.214895000
H	1.920569000	4.531788000	0.110221000
C	-0.418822000	0.793977000	3.425567000
C	-1.733677000	0.917854000	2.918879000
H	-0.046978000	-0.174825000	3.782267000
H	0.136424000	1.664729000	3.790505000
H	-2.416301000	0.059020000	2.910047000
H	-2.221631000	1.897325000	2.862933000

C	-1.740305000	-2.495327000	0.995922000
C	-1.122101000	-3.049291000	-0.148334000
C	-0.993934000	-2.427034000	2.198710000
H	-1.700209000	-3.143114000	-1.074814000
H	-1.475408000	-2.041427000	3.106083000
C	0.193408000	-3.544984000	-0.102931000
C	0.331812000	-2.896866000	2.265490000
C	0.907011000	-3.453349000	1.107378000
H	1.935857000	-3.834389000	1.150870000
C	3.443315000	-0.464969000	-1.120724000
C	3.897013000	0.703053000	-0.473116000
C	4.344213000	-1.548279000	-1.148325000
C	5.152259000	0.782432000	0.144554000
C	5.611247000	-1.494139000	-0.548987000
C	6.016573000	-0.322050000	0.104971000
F	3.997491000	-2.711059000	-1.754595000
F	6.435426000	-2.560896000	-0.590613000
F	7.229343000	-0.254812000	0.685552000
F	5.544274000	1.919427000	0.754885000
F	3.128747000	1.814252000	-0.458599000
H	-3.816915000	2.188197000	0.221171000
H	-7.072193000	-1.537346000	-1.512422000
C	-6.005407000	-1.307797000	-1.400029000
C	-5.345226000	-0.536405000	-2.339086000
C	-4.001685000	-0.152584000	-2.101170000
C	-3.279399000	-0.615989000	-0.995603000
C	-3.898101000	-1.564568000	-0.093335000
C	-5.319489000	-1.812616000	-0.255743000
C	-6.034835000	-2.551629000	0.732086000
C	-5.382063000	-3.061891000	1.840634000
C	-3.974145000	-2.947310000	1.931699000
C	-3.215690000	-2.262123000	0.974785000
H	-5.868446000	-0.162713000	-3.226191000
H	-3.445530000	-3.485575000	2.726468000
H	-5.933860000	-3.615160000	2.608586000
H	-7.111839000	-2.705137000	0.592746000
C	0.813917000	-4.202881000	-1.318126000
C	1.133296000	-2.796793000	3.547491000
H	0.480376000	-2.678539000	4.430538000
H	1.756725000	-3.695246000	3.705211000
H	1.819785000	-1.928442000	3.518663000
H	-3.544946000	0.583398000	-2.771459000
H	0.777353000	-5.306750000	-1.235524000
H	0.285480000	-3.917004000	-2.243129000
H	1.871752000	-3.913592000	-1.431263000

TS-$1_{Ins\text{-}CO\text{-}T}$

Zero-point correction= 0.583590 (Hartree/Particle)
Thermal correction to Energy= 0.632200
Thermal correction to Enthalpy= 0.633144
Thermal correction to Gibbs Free Energy= 0.498040
Sum of electronic and zero-point Energies= -2815.469296
Sum of electronic and thermal Energies= -2815.420686
Sum of electronic and thermal Enthalpies= -2815.419742
Sum of electronic and thermal Free Energies= -2815.554846
E solvent= -2815.27208693

C	2.026930000	1.571275000	1.778606000
C	1.222625000	2.327592000	2.657129000
H	0.226680000	1.963088000	2.930000000
C	1.697339000	3.538487000	3.189672000
H	1.062817000	4.111416000	3.875829000
C	2.976008000	4.009205000	2.853065000
H	3.345374000	4.951294000	3.273981000
C	3.781342000	3.264182000	1.973383000
H	4.783581000	3.620699000	1.707918000
C	3.311368000	2.056729000	1.434790000
C	-0.144653000	-0.388402000	1.846957000
C	-0.242916000	-0.888365000	3.158314000
H	0.669143000	-1.126655000	3.719575000
C	-1.499876000	-1.087637000	3.745597000
H	-1.583506000	-1.481632000	4.763650000
C	-2.656496000	-0.745910000	3.020894000
H	-3.640471000	-0.865920000	3.488128000
C	-2.593896000	-0.234624000	1.713001000
C	-1.312733000	-0.093723000	1.068015000
C	0.037688000	1.592834000	-2.376194000
Ni	0.425744000	0.529181000	-1.069129000
O	-1.255974000	0.281980000	-0.194873000
P	1.384172000	0.036960000	0.959229000
C	1.897154000	1.455406000	-2.350309000
H	2.501281000	0.694881000	-1.823148000
C	2.388327000	2.860781000	-2.016378000
H	2.093267000	3.135279000	-0.981827000
H	1.967651000	1.215964000	-3.423931000
C	3.919068000	2.992014000	-2.062087000
O	4.658963000	2.014440000	-1.983671000
O	-0.488345000	2.330512000	-3.140765000
H	1.920467000	3.618462000	-2.672957000
C	4.461959000	4.411385000	-2.177595000
H	3.985283000	5.076932000	-1.433976000
H	5.554281000	4.412577000	-2.041183000
H	4.218903000	4.824175000	-3.175455000
C	0.568977000	-1.721004000	-2.369215000
C	-0.795727000	-1.918550000	-2.690658000
C	1.123761000	-2.239335000	-1.167930000
C	-1.603214000	-2.617547000	-1.779955000
C	0.266139000	-2.919249000	-0.267948000
H	-2.661357000	-2.781513000	-2.022326000
H	0.688103000	-3.347057000	0.648633000
C	-1.092107000	-3.119329000	-0.563320000
H	1.237688000	-1.271108000	-3.114451000
C	-3.828032000	0.144793000	0.979037000
C	-3.974533000	1.416071000	0.383020000
C	-4.921774000	-0.734738000	0.849142000
C	-5.127217000	1.780511000	-0.327329000
C	-6.090039000	-0.391803000	0.154026000
C	-6.192540000	0.874683000	-0.439253000
F	-4.861688000	-1.981137000	1.383804000
F	-7.107534000	-1.271275000	0.045160000
F	-7.308911000	1.220231000	-1.108647000
F	-5.229490000	3.004868000	-0.882295000
F	-3.003351000	2.341091000	0.506014000
H	3.951152000	1.488955000	0.749037000
C	2.602505000	-2.349343000	-0.972360000
C	3.322914000	-2.984420000	-1.990379000
C	4.661838000	-3.410409000	-1.817287000
C	5.269065000	-3.263935000	-0.582339000
C	4.597737000	-2.591675000	0.480743000
C	3.273604000	-2.035786000	0.269932000
C	2.739740000	-1.178270000	1.304348000
C	3.411067000	-1.066071000	2.526368000
C	4.631463000	-1.742222000	2.774373000
C	5.234033000	-2.450399000	1.749426000
H	2.800886000	-3.229676000	-2.922129000
H	5.184056000	-3.910066000	-2.640577000
H	6.276525000	-3.656914000	-0.400171000
H	6.222191000	-2.905423000	1.890095000
H	5.119874000	-1.644511000	3.750091000
C	-1.986360000	-3.892433000	0.383676000
H	-2.382194000	-4.806709000	-0.098287000
H	-1.442072000	-4.196599000	1.293531000

H	-2.855199000	-3.288271000	0.699077000
C	-1.373755000	-1.363439000	-3.974937000
H	-1.723265000	-0.323074000	-3.830909000
H	-0.625428000	-1.348625000	-4.787774000
H	-2.239478000	-1.957232000	-4.316574000
H	3.021285000	-0.387613000	3.293194000

Catalyst 2’
1-cycle5-T

Zero-point correction= 0.612607 (Hartree/Particle)
Thermal correction to Energy= 0.653576
Thermal correction to Enthalpy= 0.654520
Thermal correction to Gibbs Free Energy= 0.538771
Sum of electronic and zero-point Energies= -2128.970256
Sum of electronic and thermal Energies= -2128.929287
Sum of electronic and thermal Enthalpies= -2128.928343
Sum of electronic and thermal Free Energies= -2129.044092
E solvent= -2128.84822573

C	0.057619000	2.604204000	1.234417000
C	1.294932000	3.200930000	1.555860000
H	2.207853000	2.847957000	1.063977000
C	1.355974000	4.246248000	2.492271000
H	2.323730000	4.703297000	2.729670000
C	0.186387000	4.708193000	3.116537000
H	0.236218000	5.525329000	3.845399000
C	-1.050412000	4.120255000	2.799233000
H	-1.969339000	4.479093000	3.277749000
C	-1.116212000	3.074283000	1.867076000
C	-1.356905000	1.620665000	-1.094151000
C	-1.452594000	2.989500000	-1.442085000
H	-0.779033000	3.711273000	-0.967842000
C	-2.402607000	3.445755000	-2.364810000
H	-2.449801000	4.510697000	-2.619199000
C	-3.291232000	2.530817000	-2.948736000
H	-4.044897000	2.870076000	-3.668750000
C	-3.211124000	1.175168000	-2.609200000
H	-3.901745000	0.457238000	-3.064555000
C	-2.250257000	0.689400000	-1.692609000
C	-2.244658000	-0.785310000	-1.433713000
C	-3.348967000	-1.409974000	-0.801215000
C	-3.404247000	-2.811472000	-0.644826000
H	-4.262075000	-3.293306000	-0.169037000
C	-2.342939000	-3.591424000	-1.131147000
H	-2.383585000	-4.681151000	-1.018415000
C	-1.239871000	-3.009981000	-1.767233000
H	-0.430462000	-3.639104000	-2.144135000
C	-1.198558000	-1.607364000	-1.926947000
C	1.571500000	1.091591000	-0.790781000
C	2.018776000	2.000586000	-1.768264000
H	1.365773000	2.814108000	-2.107403000
C	3.298236000	1.842893000	-2.306956000
H	3.670888000	2.532821000	-3.072068000
C	4.120597000	0.792527000	-1.839458000
H	5.128326000	0.711361000	-2.260524000
C	3.714593000	-0.139679000	-0.871402000
C	2.368237000	-0.017748000	-0.352550000
C	-0.680668000	-2.640675000	2.930812000
Ni	0.109173000	-0.653217000	1.248119000
O	1.868508000	-0.898933000	0.501123000
P	-0.016699000	1.145991000	0.095321000
H	-2.086740000	2.629130000	1.620152000
C	-0.633981000	-3.847062000	3.833619000
C	-1.941870000	-1.864056000	2.685413000
C	-1.572146000	-0.449656000	2.187400000
H	-2.474119000	-2.413445000	1.876599000
H	-2.616619000	-1.896650000	3.569042000
H	-2.411677000	0.000988000	1.632634000
H	-1.313412000	0.211145000	3.040245000
O	0.359125000	-2.259824000	2.339083000
O	-4.331453000	-0.553663000	-0.354105000
C	-5.495634000	-1.132761000	0.234361000
H	-6.017576000	-1.809197000	-0.470842000
H	-5.253808000	-1.690952000	1.160416000
H	-6.153638000	-0.286176000	0.483479000
O	-0.203844000	-0.941218000	-2.583014000
C	0.941813000	-1.690947000	-3.006067000
H	1.409553000	-2.212050000	-2.152102000
H	0.671468000	-2.418751000	-3.796498000
H	1.644892000	-0.945936000	-3.405323000
H	-1.438065000	-4.559405000	3.570521000
H	0.346015000	-4.343844000	3.769102000
H	-0.818297000	-3.535354000	4.879937000
C	4.656345000	-1.250226000	-0.354959000
C	4.055267000	-2.649713000	-0.658799000
H	4.712648000	-3.443157000	-0.253782000
H	3.058492000	-2.748753000	-0.199907000
H	3.967142000	-2.810079000	-1.750056000
C	4.836498000	-1.091999000	1.180327000
H	3.861236000	-1.140904000	1.690298000
H	5.484534000	-1.898538000	1.574195000
H	5.313427000	-0.123366000	1.419684000
C	6.056427000	-1.183826000	-1.008282000
H	6.691394000	-1.986793000	-0.590968000
H	6.012692000	-1.331429000	-2.103453000
H	6.563651000	-0.221704000	-0.808910000

TS_{Isom}

Zero-point correction= 0.664585 (Hartree/Particle)
Thermal correction to Energy= 0.708682
Thermal correction to Enthalpy= 0.709627
Thermal correction to Gibbs Free Energy= 0.587264
Sum of electronic and zero-point Energies= -2207.479264
Sum of electronic and thermal Energies= -2207.435166
Sum of electronic and thermal Enthalpies= -2207.434222
Sum of electronic and thermal Free Energies= -2207.556585
E solvent= -2207.37480935

C	-1.122173000	-1.939245000	-1.434443000
C	-2.111926000	-1.513708000	-2.345522000
C	-3.069151000	-2.421103000	-2.834190000
H	-3.828008000	-2.075006000	-3.545913000
C	-3.050614000	-3.762869000	-2.422655000
H	-3.794111000	-4.469571000	-2.808684000
C	-2.068441000	-4.196682000	-1.513149000
H	-2.044601000	-5.243261000	-1.187286000
C	-1.117036000	-3.292581000	-1.018519000
C	1.723399000	-1.404277000	-1.381829000
C	1.736826000	-2.225831000	-2.531461000
H	0.791861000	-2.475516000	-3.025180000
C	2.937011000	-2.738828000	-3.043859000
H	2.921019000	-3.371948000	-3.938571000
C	4.149206000	-2.440472000	-2.404606000
H	5.093788000	-2.840638000	-2.790628000
C	4.149326000	-1.621447000	-1.267264000
H	5.093580000	-1.377140000	-0.767804000
C	2.953796000	-1.087607000	-0.741245000
C	3.053015000	-0.188387000	0.454768000
C	3.282010000	-0.715259000	1.750214000
C	3.451497000	0.133079000	2.864077000

H	3.613998000	-0.274601000	3.864728000
C	3.408905000	1.522225000	2.671038000
H	3.536443000	2.186131000	3.533790000
C	3.216668000	2.080144000	1.400921000
H	3.198434000	3.165205000	1.277294000
C	3.055550000	1.222310000	0.291608000
C	-0.191175000	0.845164000	-1.477866000
C	0.046195000	1.124567000	-2.837619000
H	0.454098000	0.342173000	-3.489509000
C	-0.232135000	2.396833000	-3.344435000
H	-0.062623000	2.626319000	-4.402424000
C	-0.745538000	3.385259000	-2.479495000
H	-0.965877000	4.370130000	-2.903211000
C	-0.987205000	3.165852000	-1.112506000
C	-0.689277000	1.851691000	-0.583225000
Ni	-0.641659000	-0.132705000	1.475351000
O	-0.862835000	1.601315000	0.713674000
P	0.117919000	-0.776152000	-0.689707000
H	-0.356277000	-3.640731000	-0.309202000
C	-2.615617000	-0.185206000	1.542194000
H	-2.919265000	0.578665000	2.275346000
C	-3.180899000	-1.567494000	1.831037000
H	-2.936245000	-1.906677000	2.851179000
H	-2.834149000	0.160768000	0.519396000
C	-4.729449000	-1.514979000	1.749865000
O	-5.407360000	-1.373304000	2.761093000
H	-2.797025000	-2.300335000	1.097051000
C	-5.342903000	-1.640163000	0.362454000
H	-5.153151000	-2.654082000	-0.038355000
H	-6.427404000	-1.456727000	0.406976000
H	-4.864289000	-0.936444000	-0.342744000
C	-0.558713000	-0.501122000	3.444445000
C	-0.083090000	-1.624482000	2.729784000
H	0.149327000	0.260827000	3.801774000
H	-1.534984000	-0.502744000	3.941550000
H	0.987413000	-1.768001000	2.548588000
H	-0.701076000	-2.523222000	2.620067000
C	-1.552335000	4.280029000	-0.200558000
C	-0.547183000	4.591934000	0.942246000
H	-0.309365000	3.680917000	1.513760000
H	0.392288000	5.010024000	0.533304000
H	-0.975587000	5.343331000	1.632908000
C	-1.809641000	5.595757000	-0.973126000
H	-2.211302000	6.352953000	-0.275410000
H	-0.883561000	6.009997000	-1.413263000
H	-2.550608000	5.465857000	-1.783493000
C	-2.904730000	3.821785000	0.411817000
H	-3.316825000	4.617161000	1.061657000
H	-3.644553000	3.614420000	-0.383465000
H	-2.768300000	2.910187000	1.013371000
O	2.931245000	1.651753000	-0.999703000
C	2.877445000	3.061950000	-1.235175000
H	2.010124000	3.520616000	-0.725634000
H	3.813429000	3.559975000	-0.913811000
H	2.750510000	3.169711000	-2.321967000
O	3.310409000	-2.091973000	1.826781000
C	3.703354000	-2.674050000	3.072971000
H	4.697058000	-2.307003000	3.395025000
H	2.962464000	-2.473307000	3.871598000
H	3.752264000	-3.758223000	2.889467000
H	-2.122754000	-0.471217000	-2.681403000

TS-2$_{Coor\text{-}E\text{-}C}$

Zero-point correction= 0.673735 (Hartree/Particle)
Thermal correction to Energy= 0.720136
Thermal correction to Enthalpy= 0.721080
Thermal correction to Gibbs Free Energy= 0.594255
Sum of electronic and zero-point Energies= -2320.811955
Sum of electronic and thermal Energies= -2320.765554
Sum of electronic and thermal Enthalpies= -2320.764609
Sum of electronic and thermal Free Energies= -2320.891434
E solvent= -2320.69987572

C	0.189175000	2.740746000	1.171293000
C	1.398493000	3.451319000	1.325268000
H	2.269111000	3.167860000	0.723074000
C	1.486153000	4.525709000	2.228797000
H	2.432921000	5.069009000	2.331940000
C	0.370226000	4.905657000	2.989989000
H	0.440253000	5.743933000	3.692656000
C	-0.840012000	4.204047000	2.844866000
H	-1.718371000	4.495651000	3.433030000
C	-0.928224000	3.128139000	1.949237000
C	-1.261175000	1.873319000	-1.126024000
C	-1.285991000	3.262619000	-1.401364000
H	-0.580308000	3.920254000	-0.883633000
C	-2.199019000	3.824781000	-2.302341000
H	-2.182845000	4.904032000	-2.493351000
C	-3.127748000	2.997140000	-2.949288000
H	-3.851691000	3.416463000	-3.657593000
C	-3.123242000	1.623660000	-2.684229000
H	-3.843781000	0.970687000	-3.188634000
C	-2.203838000	1.034540000	-1.783722000
C	-2.304323000	-0.451942000	-1.624311000
C	-3.489140000	-1.043382000	-1.117828000
C	-3.666973000	-2.443479000	-1.111426000
H	-4.588816000	-2.892136000	-0.731792000
C	-2.647879000	-3.260488000	-1.624263000
H	-2.785817000	-4.348248000	-1.636072000
C	-1.464009000	-2.713737000	-2.137348000
H	-0.692843000	-3.367531000	-2.551554000
C	-1.301178000	-1.309874000	-2.148238000
C	1.600487000	1.155622000	-0.852608000
C	2.039113000	2.125278000	-1.778904000
H	1.399236000	2.985216000	-2.011503000
C	3.276810000	1.980204000	-2.406373000
H	3.628374000	2.723957000	-3.130042000
C	4.083007000	0.865797000	-2.085295000
H	5.061005000	0.788285000	-2.571209000
C	3.694593000	-0.134465000	-1.182169000
C	2.382673000	-0.015854000	-0.569395000
C	0.699219000	-2.569878000	1.848259000
Ni	0.303290000	-0.816825000	1.280977000
O	1.941097000	-0.986579000	0.215717000
P	0.032381000	1.248591000	0.072665000
H	-1.878434000	2.590956000	1.837844000
C	-0.405759000	-3.581105000	1.477666000
H	0.130015000	-0.728301000	4.204210000
C	-1.666199000	-3.369164000	2.331505000
H	-1.445624000	-3.549610000	3.406003000
H	-0.010621000	-4.599973000	1.640207000
C	-2.260960000	-1.979268000	2.205299000
O	-1.610505000	-1.021440000	1.733496000
O	1.715323000	-2.912313000	2.423829000
H	-2.441752000	-4.110825000	2.060889000
C	-3.669312000	-1.746274000	2.699808000
H	-3.723870000	-1.921438000	3.792686000
H	-3.983866000	-0.714914000	2.480290000
H	-4.370191000	-2.460466000	2.229072000
O	-4.435266000	-0.161873000	-0.638971000
C	-5.749548000	-0.667282000	-0.408434000
H	-6.152392000	-1.178652000	-1.304462000

H	-5.782318000	-1.367981000	0.449921000
H	-6.369643000	0.212300000	-0.175504000
O	-0.229348000	-0.672237000	-2.697308000
C	0.846833000	-1.470245000	-3.204128000
H	1.263755000	-2.119260000	-2.413698000
H	0.516447000	-2.080877000	-4.067620000
H	1.615541000	-0.751274000	-3.521248000
C	1.678493000	0.166313000	3.021741000
C	0.452573000	0.135084000	3.610125000
H	2.373343000	-0.674350000	3.105090000
H	2.039124000	1.067692000	2.514925000
H	-0.661866000	-3.454939000	0.409938000
H	-0.222743000	0.997300000	3.573572000
C	4.628041000	-1.321239000	-0.841869000
C	3.971378000	-2.668212000	-1.250015000
H	4.630829000	-3.512474000	-0.971295000
H	3.004039000	-2.795362000	-0.739073000
H	3.811438000	-2.712791000	-2.344062000
C	4.916035000	-1.337676000	0.684657000
H	3.982690000	-1.468324000	1.253428000
H	5.598620000	-2.173119000	0.932849000
H	5.401566000	-0.395560000	1.000472000
C	5.986869000	-1.225057000	-1.575571000
H	6.615221000	-2.088130000	-1.288961000
H	5.871039000	-1.250670000	-2.675199000
H	6.541138000	-0.306717000	-1.307200000

Catalyst 3’

1-cycle5-T

Zero-point correction= 0.627469 (Hartree/Particle)
Thermal correction to Energy= 0.667861
Thermal correction to Enthalpy= 0.668805
Thermal correction to Gibbs Free Energy= 0.554510
Sum of electronic and zero-point Energies= -2130.950231
Sum of electronic and thermal Energies= -2130.909840
Sum of electronic and thermal Enthalpies= -2130.908896
Sum of electronic and thermal Free Energies= -2131.023191
E solvent= -2130.7864077

C	0.335909000	2.591824000	0.521305000
C	1.631472000	2.813904000	1.078313000
C	1.906737000	4.081787000	1.646079000
H	2.903819000	4.248381000	2.069494000
C	0.940914000	5.092277000	1.721917000
H	1.191275000	6.056188000	2.179512000
C	-0.350220000	4.846897000	1.233797000
H	-1.130623000	5.613001000	1.304577000
C	-0.639257000	3.611263000	0.639803000
C	-1.459979000	1.577689000	-1.529739000
C	-1.107543000	2.700110000	-2.318884000
H	-0.154581000	3.205859000	-2.132189000
C	-1.956672000	3.206439000	-3.309311000
H	-1.647603000	4.073968000	-3.903158000
C	-3.201645000	2.596110000	-3.526354000
H	-3.881270000	2.975647000	-4.297802000
C	-3.566548000	1.485359000	-2.759400000
H	-4.523842000	0.986646000	-2.948343000
C	-2.716475000	0.943542000	-1.761188000
C	-3.218267000	-0.269914000	-1.053025000
C	-4.534114000	-0.279076000	-0.532887000
C	-5.076775000	-1.439665000	0.040933000
H	-6.100695000	-1.424546000	0.432814000
C	-4.311567000	-2.618942000	0.101175000
H	-4.740069000	-3.532480000	0.530127000
C	-3.002570000	-2.622869000	-0.405500000
H	-2.396640000	-3.534644000	-0.372530000
C	-2.452827000	-1.457226000	-0.970205000
C	1.120854000	0.228144000	-1.263771000
C	1.867424000	0.839253000	-2.290056000
H	1.736647000	1.904043000	-2.515601000
C	2.781636000	0.073743000	-3.013282000
H	3.381555000	0.526689000	-3.810195000
C	2.922949000	-1.300069000	-2.715137000
H	3.636792000	-1.877635000	-3.311550000
C	2.201374000	-1.953201000	-1.705567000
C	1.272633000	-1.159533000	-0.929701000
C	-1.904584000	-1.830809000	3.160153000
Ni	-0.668383000	-0.591640000	1.067664000
O	0.567931000	-1.688060000	0.062224000
P	-0.201572000	1.000161000	-0.278783000
H	-1.642253000	3.442425000	0.236002000
C	-2.235249000	-2.835434000	4.234300000
C	-2.686703000	-0.560343000	2.980818000
C	-1.814322000	0.465382000	2.225641000
H	-3.561583000	-0.840071000	2.353608000
H	-3.108260000	-0.204497000	3.946172000
H	-2.442078000	1.232581000	1.740322000
H	-1.118730000	0.979098000	2.919639000
O	-0.955992000	-2.048255000	2.367598000
H	-3.312670000	-3.083828000	4.211922000
H	-1.634107000	-3.749403000	4.113346000
H	-2.034820000	-2.393406000	5.229454000
H	-5.132098000	0.638996000	-0.577025000
H	-1.455747000	-1.498010000	-1.417457000
C	2.722074000	1.796352000	1.166199000
C	2.506411000	0.548212000	1.788303000
C	4.028737000	2.120222000	0.735238000
H	1.497414000	0.280731000	2.129913000
H	4.206779000	3.082373000	0.240257000
C	3.566573000	-0.350641000	1.974772000
C	5.086309000	1.216889000	0.912288000
H	3.375434000	-1.316574000	2.454431000
H	6.089751000	1.478910000	0.557220000
C	4.859544000	-0.020123000	1.538168000
H	5.686553000	-0.725347000	1.679938000
C	2.376306000	-3.462620000	-1.422630000
C	1.015656000	-4.190196000	-1.603762000
H	1.129063000	-5.270714000	-1.390989000
H	0.263078000	-3.772127000	-0.916218000
H	0.648879000	-4.083968000	-2.641676000
C	2.881660000	-3.671305000	0.031048000
H	2.174778000	-3.230600000	0.751626000
H	2.984933000	-4.752346000	0.247041000
H	3.870760000	-3.197808000	0.171438000
C	3.395876000	-4.123321000	-2.379723000
H	3.477838000	-5.199050000	-2.138412000
H	3.085514000	-4.042186000	-3.437983000
H	4.405214000	-3.682927000	-2.279926000

TS_{Isom}

Zero-point correction= 0.679344 (Hartree/Particle)
Thermal correction to Energy= 0.722987
Thermal correction to Enthalpy= 0.723931
Thermal correction to Gibbs Free Energy= 0.601983
Sum of electronic and zero-point Energies= -2209.457447
Sum of electronic and thermal Energies= -2209.413804
Sum of electronic and thermal Enthalpies= -2209.412860
Sum of electronic and thermal Free Energies= -2209.534808
E solvent= -2209.30976651

C	-0.819541000	-2.156459000	0.094036000
C	-2.183546000	-2.295001000	-0.296374000
C	-2.887135000	-3.454387000	0.125377000
H	-3.937861000	-3.549710000	-0.171988000
C	-2.296716000	-4.445914000	0.915230000
H	-2.879596000	-5.321060000	1.224137000
C	-0.959633000	-4.297374000	1.317754000
H	-0.475965000	-5.054374000	1.945448000
C	-0.245518000	-3.165053000	0.912255000
C	1.598532000	-1.782827000	-1.414878000
C	1.080441000	-2.816649000	-2.228616000
H	0.008396000	-3.038880000	-2.190895000
C	1.906065000	-3.581242000	-3.063745000
H	1.471252000	-4.371567000	-3.686097000
C	3.285707000	-3.327927000	-3.092011000
H	3.946414000	-3.914223000	-3.740613000
C	3.817386000	-2.312573000	-2.288686000
H	4.891727000	-2.098284000	-2.324357000
C	3.002191000	-1.520676000	-1.443809000
C	3.674340000	-0.472064000	-0.623884000
C	4.854828000	-0.790623000	0.089389000
C	5.528128000	0.182597000	0.843480000
H	6.436338000	-0.089951000	1.393357000
C	5.037549000	1.499013000	0.899058000
H	5.566234000	2.260407000	1.483744000
C	3.870331000	1.831879000	0.193216000
H	3.481081000	2.855487000	0.214638000
C	3.193085000	0.857518000	-0.561637000
C	-0.177877000	0.517477000	-1.360713000
C	-0.492520000	0.388434000	-2.728898000
H	-0.439856000	-0.594629000	-3.210534000
C	-0.873018000	1.511493000	-3.460960000
H	-1.127970000	1.425348000	-4.522656000
C	-0.925491000	2.766848000	-2.820475000
H	-1.223516000	3.632065000	-3.420844000
C	-0.604260000	2.959103000	-1.467558000
C	-0.205668000	1.796784000	-0.703853000
Ni	0.883011000	0.493423000	1.609430000
O	0.158346000	1.936150000	0.571367000
P	0.442633000	-0.835471000	-0.304556000
H	0.800085000	-3.057146000	1.226300000
C	-0.715906000	0.708273000	2.749498000
H	-0.597438000	1.664396000	3.283209000
C	-0.982515000	-0.476221000	3.666115000
H	-0.216142000	-0.561678000	4.455026000
H	-1.468676000	0.815816000	1.953102000
C	-2.339205000	-0.268255000	4.389322000
O	-2.388659000	0.246070000	5.500414000
H	-1.016088000	-1.415493000	3.083082000
C	-3.584134000	-0.729090000	3.642617000
H	-3.580533000	-1.832966000	3.561683000
H	-4.491044000	-0.403521000	4.174812000
H	-3.591759000	-0.341532000	2.607287000
C	2.033885000	0.716267000	3.249454000
C	2.125234000	-0.609596000	2.766287000
H	2.781000000	1.460479000	2.943487000
H	1.469725000	0.960170000	4.156291000
H	2.960429000	-0.900195000	2.118152000
H	1.608364000	-1.430909000	3.275243000
C	-0.644631000	4.362352000	-0.818486000
C	0.771268000	4.744586000	-0.304686000
H	1.126708000	4.008239000	0.433650000
H	1.491267000	4.785700000	-1.143344000
H	0.748076000	5.742750000	0.172810000
C	-1.090778000	5.457013000	-1.816819000
H	-1.108286000	6.433837000	-1.300001000
H	-0.398545000	5.548662000	-2.674028000
H	-2.107512000	5.272584000	-2.210312000
C	-1.646932000	4.370403000	0.367816000
H	-1.656576000	5.365361000	0.852298000
H	-2.672952000	4.156968000	0.013901000
H	-1.367184000	3.615853000	1.119081000
H	2.323630000	1.147383000	-1.160256000
H	5.232874000	-1.819568000	0.066504000
C	-2.974679000	-1.323167000	-1.114064000
C	-3.208309000	-0.000352000	-0.682027000
C	-3.609094000	-1.765807000	-2.296793000
H	-2.739683000	0.355437000	0.241436000
H	-3.439829000	-2.793110000	-2.641547000
C	-4.039171000	0.858241000	-1.415604000
C	-4.436700000	-0.905984000	-3.034925000
H	-4.202334000	1.882715000	-1.063943000
H	-4.911260000	-1.265915000	-3.955168000
C	-4.654678000	0.409192000	-2.595279000
H	-5.302181000	1.082137000	-3.168783000

TS-2$_{Coor\text{-}E\text{-}C}$

Zero-point correction= 0.688446 (Hartree/Particle)
Thermal correction to Energy= 0.734623
Thermal correction to Enthalpy= 0.735567
Thermal correction to Gibbs Free Energy= 0.608269
Sum of electronic and zero-point Energies= -2322.790512
Sum of electronic and thermal Energies= -2322.744335
Sum of electronic and thermal Enthalpies= -2322.743391
Sum of electronic and thermal Free Energies= -2322.870689
E solvent= -2322.63518357

C	1.354890000	-2.051929000	-0.901697000
C	2.762771000	-2.061593000	-1.124621000
C	3.307308000	-3.103991000	-1.921201000
H	4.389256000	-3.099203000	-2.096858000
C	2.515831000	-4.104008000	-2.494777000
H	2.978882000	-4.886524000	-3.106673000
C	1.126823000	-4.080973000	-2.292311000
H	0.483262000	-4.847012000	-2.740163000
C	0.566876000	-3.062211000	-1.513993000
C	-0.189972000	-2.065790000	1.513236000
C	0.690636000	-3.133370000	1.811068000
H	1.604110000	-3.248802000	1.219853000
C	0.425328000	-4.062017000	2.826489000
H	1.140009000	-4.868360000	3.027195000
C	-0.754295000	-3.948260000	3.573917000
H	-0.983563000	-4.663130000	4.372425000
C	-1.635319000	-2.895344000	3.302738000
H	-2.541373000	-2.779275000	3.908460000
C	-1.382038000	-1.935495000	2.291224000
C	-2.396895000	-0.845076000	2.162500000
C	-3.773898000	-1.175625000	2.133323000
H	-4.067279000	-2.232091000	2.105152000
C	-4.759994000	-0.177615000	2.146344000
H	-5.820077000	-0.459230000	2.136802000
C	-4.389442000	1.178220000	2.195580000
H	-5.157163000	1.960366000	2.226705000
C	-3.027157000	1.520660000	2.218277000
H	-2.721677000	2.572113000	2.260938000
C	-2.039847000	0.521337000	2.197225000
H	-0.983871000	0.802523000	2.253291000
C	1.150935000	0.453167000	0.914651000
C	2.041376000	0.306918000	1.999076000
H	2.274073000	-0.694388000	2.379889000
C	2.619326000	1.436536000	2.573140000
H	3.316151000	1.343421000	3.413003000

C	2.294863000	2.711803000	2.059412000
H	2.764332000	3.582680000	2.528499000
C	1.395825000	2.917026000	1.002594000
C	0.777118000	1.746450000	0.410190000
C	1.044793000	4.336815000	0.500907000
C	1.838991000	5.436090000	1.245088000
H	1.624784000	5.445340000	2.329909000
H	1.553090000	6.425256000	0.842894000
H	2.930669000	5.324295000	1.108663000
C	1.365605000	4.465810000	-1.013140000
H	1.114136000	5.483413000	-1.368914000
H	0.785568000	3.735635000	-1.597737000
H	2.443306000	4.298948000	-1.199024000
C	-0.467677000	4.608567000	0.732661000
H	-0.702985000	4.598617000	1.813900000
H	-1.075885000	3.843016000	0.225742000
H	-0.744362000	5.603014000	0.332878000
C	-2.265992000	1.739918000	-2.004953000
Ni	-1.171929000	0.392822000	-1.264543000
O	-0.109159000	1.891136000	-0.565423000
P	0.266488000	-0.912189000	0.110262000
H	-0.519138000	-3.047340000	-1.354792000
C	-3.725761000	1.695472000	-1.506862000
H	-3.716208000	1.700102000	-0.401890000
C	-4.478206000	0.449443000	-2.002690000
H	-4.552380000	0.456614000	-3.112300000
H	-4.238427000	2.606048000	-1.866015000
C	-3.839567000	-0.866232000	-1.602554000
O	-2.630781000	-0.950903000	-1.295137000
O	-1.876718000	2.597803000	-2.775721000
H	-5.523031000	0.462336000	-1.638233000
C	-4.702268000	-2.105794000	-1.584641000
H	-5.333897000	-2.163490000	-2.490885000
H	-4.082045000	-3.010966000	-1.498769000
H	-5.388362000	-2.060525000	-0.716312000
C	4.850839000	-1.529894000	0.163932000
C	5.855436000	-0.646954000	0.588634000
C	3.753623000	-1.069463000	-0.598775000
H	6.692535000	-1.023687000	1.187987000
C	5.785934000	0.712292000	0.245933000
C	3.698103000	0.299411000	-0.937530000
H	6.569615000	1.404829000	0.573897000
H	2.865065000	0.677957000	-1.537936000
C	4.704951000	1.180745000	-0.518586000
H	4.638865000	2.240275000	-0.788499000
H	4.906888000	-2.592067000	0.431590000
C	-0.741698000	-0.494718000	-3.622852000
C	0.262935000	0.337560000	-3.242956000
H	-1.641137000	-0.110190000	-4.117469000
H	-0.659735000	-1.581031000	-3.505818000
H	0.203164000	1.418669000	-3.400503000
H	1.198250000	-0.065985000	-2.841350000

8 References

(1) Göttker-Schnetmann, I.; Mecking, S. *Organometallics* **2020**, *39*, 3433-3440.

(2) Rezai, N.; Meybodi, F.A.; Salehi, P. *Synth. Commun.* **2000**, *30*, 1799-1805.

(3) Zhang, Y.; Mu, H.; Pan, L.; Wang, X.; Li, Y. *ACS Catal.* **2018**, *8*, 5963-5976.

(4) Neuwald, B.; Ölscher, F.; Schnetmann, I.G.; Mecking, S. *Organometallics* **2012**, *31*, 3128-3137.

(5) Zhang, Y.; Mu, H.; Wang, X.; Pan, L.; Li, Y. *ChemCatChem* **2019**, *11*, 2329–234.

(6) Baur, M.; Lin, F.; Morgen, T.O.; Odenwald, L.; Mecking, S. *Science* **2021**, *374*, 604–607.

(7) (a) Perdew, J. P. *Phys. Rev. B* **1986**, *33*, 8822. (b) Perdew, J. P.; *Phys. Rev. B* **1986**, *34*, 7406; (c) Becke, A. D.; *Phys. Rev. A* **1988**, *38*, 3098.

(8) Frisch, M. J.; Trucks, G. W.; Schlegel, H. B.; Scuseria, G. E.; Robb, M.A.; Cheeseman, J. R.; Scalmani, G.; Barone, V.; Mennucci, B.; Petersson, G. A.; Nakatsuji, H.; Caricato, M.; Li, X.; Hratchian, H. P.; Izmaylov, A. F.; Bloino, J.; Zheng, G.; Sonnenberg, J. L.; Hada, M.; Ehara, M.; Toyota, K.; Fukuda, R.; Hasegawa, J.; Ishida, M.; Nakajima, T.; Honda, Y.; Kitao, O.; Nakai, H.; Vreven, T.; Montgomery, J.A.; Peralta, J. E.; Ogliaro, F.; Bearpark, M.; Heyd, J.J.; Brothers, E.; Kudin, K. N.; Staroverov, V.N.; Kobayashi, R.; Normand, J.; Raghavachari, K.; Rendell, A.; Burant, J.C.; Iyengar, S.S.; Tomasi, J.; Cossi, M.; Rega, N.; Millam, J.M.; Klene, M.; Knox, J. E.; Cross, J.B.; Bakken, V.; Adamo, C.; Jaramillo, J.; Gomperts, R.; Stratmann, R.E.; Yazyev, O.; Austin, A.J.; Cammi, R.; Pomelli, C.; Ochterski, J.W.R.; Martin, L.; Morokuma, K.; Zakrzewski, V.G.; Voth, G.A.; Salvador, P.; Dannenberg, J.J.; Dapprich, S.; Daniels, A.D.; Farkas, Ö.; Foresman, J.B.; Ortiz, J.V.; Cioslowski, J.; Fox D. J. Gaussian 09 Revision A.1, Gaussian, Inc., Wallingford, CT **2009**.

(9) Weigend F.; Ahlrichs R. *PCCP*, **2005**, *7*, 3297-3305.

(10) (a) Fukui K. *J. Phys. Chem.* **1970**, *74*, 4161-4163; (b) Fukui K. *Acc. Chem. Res.* **1981**, *14*, 363-368.

(11) (a) Häussermann U.; Dolg M.; Stoll H.; Preuss H.; Schwerdtfeger P.; Pitzer R.M. *Mol. Phys.* **1993**, *78*, 1211. (b) Küchle W.; Dolg M.; Stoll H.; Preuss H. *J. Chem. Phys.* **1994**, *100*, 7535. (c) Leininger T.; Nicklass A.; Stoll H.; Dolg M.; Schwerdtfeger P. *J. Chem. Phys.* **1996**, *105*, 1052.

(12) (a) Tomasi J.; Persico M. *Chem. Rev.* **1994**, *94*, 2027. (b) Barone V.; Cossi M. *J. Phys. Chem. A* **1998**, *102*, 1995.

(13) Falivene L.; Cao Z.; Petta A.; Serra L.; Poater A.; Oliva R.; Scarano V.; Cavallo L. *Nat. Chem.* **2019**, *11*, 872–879.

(14) Adamo C.; Barone V. *J. Chem. Phys.* **1999**, *110*, 6158-6170.

(15) Grimme S.; Antony J.; Ehrlich S.; Krieg H. *J. Chem. Phys.* **2010**, *132*, 154104.

(16) Perdew J.P.; Wang Y. *Phys. Rev. B* **1992**, *45*, 13244.

(17) Marenich A. V.; Cramer C. J.; Truhlar D. G. *J. Phys. Chem. B* **2009**, *113*, 6378-96.

(18) Haras A.; Michalak A.; Rieger B.; Ziegler T. *J.Am.Chem. Soc.* **2005**, *127*, 8765-8774.

(19) Jáuregui-Haza U. J.; Pardillo-Fontdevila E. J.; Wilhelm A. M.; Delmas H. *Lat. Am. appl. res.* **2004**, *34*, 71-74.

(20) Schuster N.; Rünzi T.; Mecking S. *Macromolecules* **2016**, *49*, 1172−1179.

(21) Shultz C.S.; Ledford J.; DeSimone J.M.; Brookhart M. *J. Am. Chem. Soc.* **2000**, *122*, 6351-6356.
